# Inhibition of TRPV4 remodels single cell polarity and suppresses the metastasis of hepatocellular carcinoma

**DOI:** 10.1038/s41419-023-05903-z

**Published:** 2023-06-28

**Authors:** Jian Liu, Yongjian Guo, Ruitian Zhang, Ye Xu, Chengju Luo, Rui Wang, Shu Xu, Libin Wei

**Affiliations:** 1 State Key Laboratory of Natural Medicines, Jiangsu Key Laboratory of Carcinogenesis and Intervention, School of Basic Medical Sciences and Clinical Pharmacy, China Pharmaceutical University, #24 Tongjiaxiang, Nanjing, The People’s Republic of China; 2 School of Biopharmacy, China Pharmaceutical University, #639 Longmian Dadao, Nanjing, The People’s Republic of China

**Keywords:** Metastasis, Oncogenes, Drug development

## Abstract

Hepatocellular carcinoma (HCC) is a malignant tumor, frequently causing both intrahepatic and extrahepatic metastases. The overall prognosis of patients with metastatic HCC is poor. Recently, single-cell (sc) polarity is proved to be an innate feature of some tumor cells in liquid phase, and directly involved in the cell adhesion to blood vessel and tumor metastasis. Here, we characterize the maintained sc polarity of HCC cells in a suspension culture, and investigate its roles and regulatory mechanisms during metastasis. We demonstrate that transient receptor potential vanilloid 4 (TRPV4) is a promoting regulator of sc polarity via activating Ca^2+^-dependent AMPK/MLC/ERM pathway. This attenuates the adhesion of metastatic HCC cells to vascular endothelial cells. The reduction of cancer metastases can result from TRPV4 inhibition, which not only impacts the migration and invasion of tumor cells, but also prevents the adhesion to vascular endothelial cells. Additionally, we discover a brand-new TRPV4 inhibitor called GL-V9 that modifies the degree of sc polarization and significantly decreases the metastatic capacity of HCC cells. Taken together, our data shows that TRPV4 and calcium signal are significant sc polarity regulators in metastatic HCC, and that the pharmacological intervention that results in HCC cells becoming depolarized suggests a promising treatment for cancer metastasis.

## Introduction

Hepatocellular carcinoma (HCC) is the leading cause of cancer-related death worldwide [1, 2]. Currently, despite significant advances in HCC treatment, metastasis persists as a major barrier to successful therapy [3]. Metastasis is a complicated, multistep biological process, implicating multiple genes and biomolecules [4, 5]. Metastatic tumor cells in the circulatory system possess capabilities to evade the immune surveillance, adapt the blood flow shear force [6], and maintain anchorage-independent growth [7]. Understanding the molecular regulations of metastasizing cancer cells would thus facilitate the new discovery of effective drug targets in HCC therapy.

Single-cell (Sc) polarity is a type of cell polarity exhibited by a single cell in the liquid phase. Sc polarity is characterized by the accumulation of ezrin, radixin and moesin (ERM, E for ezrin, R for radixin and M for moesin) proteins and F-actin proteins in the cellular cortex to form a dot or cap structure [8]. The scaffold protein ERM is in charge of joining the F-actin to plasma membrane [9]. The formation of sc polarity depends on the phosphorylation of ERM protein that opens the N-terminal 4.1-ezrin-radixin-moesin (FERM) domain [10] and exposes the F-actin binding site, coordinating the cytoskeleton with the membrane structure to form a special cellular cortex [11]. Some types of tumor cells, including melanoma, glioblastoma and gastric cancer, are reported to maintain sc polarity and facilitate tumor metastasis via impacting adhesion to blood vessel and transmigration [8, 12]. The polar accumulation of F-actin co-localizes with the β1-Integrin, melanoma cell adhesion molecule (MCAM) and intercellular adhesion molecule-1, which may contribute to the improved adhesion of tumor cells.

Myosin light chain (MLC) phosphorylation is crucial for many cellular processes, including focal adhesion, movement and cellular morphogenesis [13, 14]. It has been demonstrated that phosphorylation of MLC is essential for MCAM’s (melanoma cell adhesion molecule) ability to induce sc polarity [8, 15]. The significance for metastasis and the control of tumor sc polarization, however, are still mainly unknown.

Oncogenic calcium signaling is essential for driving tumor invasion-metastasis cascade and its translational potential [16, 17]. A member of the TRP family, transient receptor potential vanilloid 4 (TRPV4) is a calcium permeable non-selective cation channel that contributes to the growth and structural integrity of a variety of organs, including liver, kidney, bladder and others [18–20]. Warm temperatures, mechanical forces, and lipid mediators like arachidonic acid are just a few of the stimuli to activate TRPV4 [21]. According to research, TRPV4 level is higher in cancer cells from stomach, lung, and colon but lower in those from esophagus and prostate [22]. Abnormal expression of TRPV4 is involved in the occurrence, growth and metastasis in types of cancer [21–24]. Inhibition of TRPV4 suppresses cell proliferation and attenuates the epithelial–mesenchymal transition (EMT) of HCC via modulating ERK pathway [23]. Activation of TRPV4 affects calcium ion influx and induces a series of Ca^2+^-dependent regulations that are involved in tumorigenesis. Adenosine 5‘-monophosphate (AMP)-activated protein kinase (AMPK) is phosphorylated and activated by Ca^2+^-dependent protein kinase kinase beta (CaMKKβ), and it is crucial for the development and energy metabolism of cancer cells [24]. TRPV4 may be an important AMPK regulator.

Here, we demonstrate that HCC cells in liquid phase maintain sc polarity and investigate how it is regulated by TRPV4 during metastasis. Our hypothesis is that TRPV4 regulates sc polarity by modifying calcium flux and the Ca^2+^-dependent signal pathway. Additionally, it has been discovered that the novel TRPV4 inhibitor GL-V9 effectively inhibits the metastasis of HCC by rewriting the sc pole. GL-V9 is a synthetic derivative of bioactive flavonoid wogonin originated from Scutellaria baicalensis, and exhibits superior antitumor effects to wogonin in several types of tumors by inducing cell apoptosis, cycle arrest and autophagy [25–28]. However, it is unclear how GL-V9 works to prevent metastasis. This research demonstrates a novel mechanism by which GL-V9 inhibits tumor metastasis by reducing TRPV4-regulated sc polarity, and provides a promising candidate for HCC therapy.

## Materials and methods

### Cell culture

Human hepatocellular carcinoma cells MHCC-97H and HCC-LM3, and human umbilical vein endothelial cells (HUVEC) were purchased from Cell Bank, Chinese Academy of Sciences (Shanghai, China). HCC-LM3 cells were cultured in DMEM, MHCC-97H and HUVEC cells were cultured in RPMI-1640, supplemented with 10% Fetal Bovine Serum (FBS, Wisent, Inc., CAN, Quebec, QC, Canada) and 100 U/ml of streptomycin and penicillin (GIBCO, Thermo Fisher Scientific Inc., Chino, CA, USA) at 37 °C with 5% CO_2_.

The anchorage-independent growth of cells was performed in six-well plate coated with poly-2-hydroxyethylmethacrylate (poly-HEMA, Sigma-Aldrich, St. Louis, MO, USA). Poly-HEMA powder was dissolved to 12 mg/mL in 95% ethanol.

### Reagents

GL-V9 (C_24_H_27_NO_5_, (5-hydroxy-8-methoxy-2-phenyl-7-(4-(pyrro-lidin-1-yl) butoxy)4H-chromen-4-one), MW 409.47, purity >99%) was kindly provided by Prof. Zhiyu Li (China Pharmaceutical University, China) and dissolved in dimethyl sulfoxide (DMSO, Sigma-Aldrich, St. Louis, MO, USA) to 0.01 M as the parent solution stored at −80 °C. TRPV4 specific agonist GSK1016790A (# HY-19608) and inhibitor HC067047 (# HY-100208) and the myosin II inhibitor blebbistatin (#HY-13441) were purchased from MedChem Express and dissolved in DMSO to 5 mM stored at −80 °C.

### Intracellular calcium ion measurement

Treated cells were collected and incubated with Fluo-4 AM for 30 min, then washed with PBS three times, and resuspended in HBSS buffer. Using flow cytometry, the cells’ basal calcium ion levels were first determined. Next, the cells were activated with GSK1016790A, and the stimulation values were determined. The data was processed with FlowJo-V10, and the experiment was repeated three times.

### Observation of sc polarity

The fixed cells were stained with rhodamine-labeled phalloidin for 30 min. After incubation and washing, the coverslips were added 15–20 μL of anti-quenching mounting tablets containing DAPI, and placed in the dark for 5 min. Using a confocal camera to take pictures of the cells in the field of view, sc polarity was assessed using F-actin or Ezrin punctate, or cap-like structures. In every experiment, at least 5 pictures were produced, and each photograph had more than 30 cells. Three times the trial was conducted.

### In vitro adhesion to HUVEC assay

HUVEC were seeded in a six-well plate containing glass coverslips, and the next day they grew into a dense cell monolayer. MHCC-97H cells were cultured in suspension and treated, then stained with Dil (Beyotime, Shanghai, China) for 30 min, washed twice with serum-free medium and collected. The collected MHCC-97H cells at a density of 100,000 cells per well were inoculated in the six-well plate containing coverslips with HUVEC monolayer. MHCC-97H cells and HUVEC were co-incubated for 1 h in the serum-free medium, gently washed twice with PBS, then placed in the dark at room temperature for 10 min. Adherent MHCC-97H cells were photographed using a fluorescence microscope; each set could have up to 7 images. The experiment was repeated three times and the data was processed with Imagine J to obtain the average fluorescence intensity.

### In vitro transendothelial cell migration assay

This experiment was used for evaluate the extravasation migration of suspension-grown HCC cells in vitro. Matrigel (6 mg/mL) was spread in the upper chamber of the transwell chamber. 5×10^4^ HUVEC were added after solidification and grew into a dense cell monolayer in about 24 h. MHCC-97H cells with Dil-stained were planted in the upper chamber at a density of 30,000 per well upon treatment for 24 h, and the lower chamber was 15% FBS medium. After that, the extravasation migration of MHCC-97H cells through HUVEC monolayer was captured using a fluorescence microscope with three fields of view in each chamber. The experiment was repeated three times and the number of migrated cells was quantified.

### Docking study

The crystal structure of Xenopus tropicalis TRPV4 (PDB code: 6BBJ and resolution 3.80 Å) was used for a docking investigation, and the structure was protonated using GOLD 5.1 [29]. GL-V9 structure was drawn by ChemBioDraw Ultra 14.0 software and then saved in MOL format. For computational docking, GOLD 5.1 software was used in combination with Goldscoring function. The active site was defined as being any volume within 8 Å of the scaffold of native ligand in its crystal pose in 6BBJ. Each GOLD run was saved and the strongest scoring binding pose of GL-V9 was compared to that of the reference ligand position observed in the crystal structure. The best output poses of the ligands generated were analyzed on the basis of Goldscore, feasibility of hydride transfer process, and H-bonding to the enzyme. The best poses were visualized with MOE 2020.0901.

### Microscale thermophoresis

The equilibrium dissociation constant (Kd) values were measured by using the Monolith NT.115 instrument (NanoTemper Technologies). The TRPV4 proteins were fluorescently labeled according to the manufacturer’s protocol. GL-V9 was diluted to the indicated concentration (from 7.6 × 10^−4 ^μM to 25 μM) and incubated with 200 nM of purified labeled TRPV4 protein in running buffer (50 mmol/L Tris-HCl, 100 mmol/L NaCl, pH 7.5). The samples were loaded into the NanoTemper glass capillaries and microthermophoresis was carried out using 80% light emitting diode power and 80% microscale thermophoresis (MST). The Kd values were calculated using the mass action equation via the MO. Affinity Analysis v2.3 software from duplicate reads of measurements.

### Animal studies

All animals received humane care according to the criteria outlined in the Guide for the Care and Use of Laboratory Animals prepared by the National Academy of Sciences and published by the National Institutes of Health. Female BALB/c nude mice (6–8 weeks old) weighing 18–22 g were purchased from the Academy of Military Medical Sciences of the Chinese People’s Liberation Army (Certificate No. SCXK(ZHE)2019-0004).

For in vivo adhesion assay of metastatic tumor cells to blood vessel, the nude mice were injected with 5 × 10^6^ GFP- labeled MHCC-97H cells with or without treatment into tail vein. and then sacrificed 2 h later. The liver and lungs of nude mice were collected and fixed in 4% paraformaldehyde and then sent for paraffin sections. CD31 (Bioworld, BS1574, 1:200) was used as a blood vessel marker. the tumor cells adhered to the blood vessel in the liver and lungs were photographed by the confocal microscope.

For metastasis models via intravenous tail vein injections, the nude mice were grouped by weight (*n* = 5), and 7 × 10^6^ MHCC-97H cells per mouse were injected into the tail vein. The administration was started on the second day, and divided into the following 5 groups: control group (normal saline, i.p., daily), 0.1 mg/kg GSK-1016790A group (i.p. daily), 300 mg/kg GL-V9 (micronized and dissolved in CMCNa, p.o. every other day), 10 mg/kg HC-067047 (i.p. every other day), and a combined group in which mice were administrated with 300 mg/kg GL-V9 (p.o.) combing with 0.1 mg/kg GSK-1016790A (i.p.) alternately. All the mice were sacrificed and dissected in the 7th week of the experiment. The lungs were fixed in Bouin’s solution for 12 h and washed with 70% ethanol to determine lung metastasis, and the livers were formalin-fixed.

### Western blotting assay

Cells were collected and lysed with a lysis buffer containing protease inhibitors and phosphatase inhibitors to extract total protein. The target protein separated by SDS-polyacrylamide gel, and transferred to NC membrane. Then the Amersham Imager 600 (GE Healthcare, Little Chalfont, UK) were used to detect specific protein bands. Primary antibodies included antibodies against β-Actin (ABclonal, AC026, 1:200,000), TRPV4 (Proteintech, 20987-1-AP; 1:2000), P-ERM (abcam, ab76247; 1:1000), P-MLC (Cell Signaling Technology, 3671 T; 1:1000), P-AMPK (ABclonal, AP1002; 1:2000), ERM (abbkine, ABP55285; 1:2000), MLC (Cell Signaling Technology, 3672; 1:2000), AMPK (ABclonal, A1229; 1:2000), ROCK1 (Cell Signaling Technology, 4035 T; 1:1000), E-Cadherin (Cell Signaling Technology, 3195 T; 1:1000), N-Cadherin (Cell Signaling Technology, 13116 T; 1:1000), Vimentin (Cell Signaling Technology, 5741 T; 1:1000), MMP2 (ABclonal, A19080, 1:800), MMP9 (ABclonal, A0289, 1:600). Western blotting assay for each protein was performed at least three times.

All the full and uncropped western blots was shown in Supplemental Material of Original Western Blots Data.

### Statistical

Each experiment was performed at least three times, and the values of three independent experiments are expressed as the mean ± standard deviation (SD). T-test was used to compare two groups, and one way analysis of variance (ANOVA) was used to compare multiple groups. *Bar*, SD. ^*^P < 0.05, ^**^P < 0.01, ^***^P < 0.001 or ^****^P < 0.0001 compared with control.

### Bioinformatics analysis

TRPV family protein expression in HCC patients were analyzed by using the data from The Human Protein Atlas (https://www.proteinatlas.org/). The RNA expression of TRPV channel family in HCC cases, and clinical traits of hepatocellular carcinoma patients who express *TRPV2* or *TRPV4* was shown in Supplementary Tables 1, 2 and Supplementary Fig. S1B, respectively. Survival curves of clinical liver cancer patients expressing TRPV2 or TRPV4 were analyzed using the Kaplan-Meier Plotter (http://kmplot.com/analysis/index.php?p=background).

We further analyze the *TRPV2* or *TRPV4* gene-related survival curves in patients with non-stage I liver cancer. The detailed patients’ information of non-stage I HCC cases with *TRPV2* or *TRPV4* RNA low and high expression was listed in Supplementary Tables 3.

## Results

### TRPV4 is highly expressed in HCC patients and promotes the migration and invasion of HCC cells by influencing cytoskeleton-related proteins

**Fig. 1:**
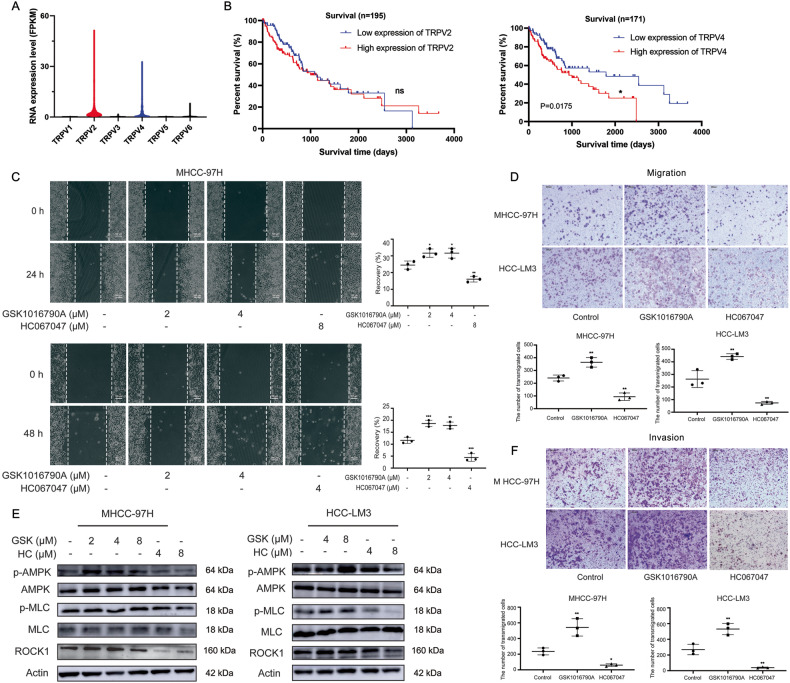
TRPV4 regulates the migration and invasive of HCC cells. **A** TRPV family protein RNA expression levels in HCC patients. **B** Kaplan-Meier overall survival curves of non-stage I HCC cases with low and high *TRPV2* or *TRPV4* RNA expression. The patient survival proportion is plotted versus time since diagnosis in months. In analyses of Kaplan-Meier curves with a log-rank test, *P* value < 0.05 was considered significant. *ns* means no significance. **C** GSK1016790A and HC-06747 were applied to MHCC-97H cells or HCC-LM3 cells for 24 h or 48 h, respectively. Wound healing assay was done and scratch healing rate was quantified. Scale bar: 100 μm. **D** MHCC-97H cells were treated with 4 μM GSK1016790A or 8 μM HC067047 for 24 h, while HCC-LM3 cells were treated with 4 μM GSK1016790A or 4 μM HC067047 for 24 h. Transwell migration assay without Matrigel matrix was detected and the number of transmigrated cells were quantified. Scale bar: 200 μm. **E** The expression of cytoskeleton-related proteins was measured by western blot. **F** MHCC-97H cells or HCC-LM3 cells received the same treatment as described in **D**. Transwell invasion assay with Matrigel matrix was detected and the number of transmigrated cells were quantified. Scale bar: 200 μm. *Bar*, SD. ^*^P < 0.05, ^**^P < 0.01, ^***^P < 0.001 or ^****^P < 0.0001 versus the untreated control.

Of the TRPV channel family, TRPV2 and TRPV4 have been mostly studied. We investigated RNA expression of TRPV family protein in HCC through TCGA database and found that *TRPV2* and *TRPV4* were highly expressed in clinical HCC patients (Fig. 1A). Kaplan-Meier survival curve analysis found that *TRPV2* and *TRPV4* were significantly associated with HCC patients at stage ii&iii and stage iii&iv, but not at stage all and stage i and ii (Supplementary Fig. S1A). In order to make a more precise assessment of the prognostic relationship between metastatic HCC and *TRPV2* or *TRPV4*, we excluded the data of HCC patients in stage I, which had no nodal spread and extrahepatic metastases. Analysis of Kaplan-Meier overall survival curves showed that there was a significantly positive correlation between the high expression of *TRPV4* RNA and the poor overall survival in non-stage I HCC patients, but *TRPV2* not (Fig. 1B). Therefore, we hypothesized that highly expressed *TRPV4* was crucial to the development of metastatic HCC.

Then, the role of TRPV4 in the migration and invasion of HCC cells were investigated using TRPV4 specific agonist GSK1016790A and inhibitor HC067047. Applying concentrations without significant growth inhibition (Supplementary Fig. S2A), GSK increased the wound healing capacity of MHCC-97H and HCC-LM3 cells in a scratch assay, whereas HC067047 reduced it (Fig. 1C). Similar results were showed in transwell migration assay or transwell invasion assay, respectively (Fig. 1D, F). Thus, TRPV4 regulated the migration and invasion of HCC cells. Mechanically, either activation or inhibition of TRPV4 had little effects in the protein expression of epithelial–mesenchymal transition markers E-cadherin, N-cadherin, vimentin, as well as matrix metalloproteinase-2 (MMP-2) and MMP-9 (Supplementary Fig. S2B). Recent research have demonstrated that TRPV4 activation induces Ca^2+^-dependent ATP release, which in turn modulates the mechanosensation and wound healing in esophageal epithelial cells [30]. In MHCC-97H and HCC-LM3 cells, the regulation of TRPV4 by GSK1016790A or HC067047 had no effect on ATP production or glycolysis (Supplementary Fig. S2C), but it substantially altered the level of cytoskeleton-related protein MLC and ROCK1. AMPK can phosphorylate and activate MLC [31]. GSK1016790A increased the phosphorylation of AMPK and MLC as well as ROCK1 protein expression, while HC067047 had the opposite impacts (Fig. 1E). As a result, TRPV4 interferes with AMPK/MLC and ROCK1 to affect cytoskeleton and promote the migration and invasion of HCC cells.

### TRPV4 alters single cell polarity that HCC cells maintain in the liquid phase by regulating Ca^2+^-dependent AMPK/MLC/ERM axis

**Fig. 2:**
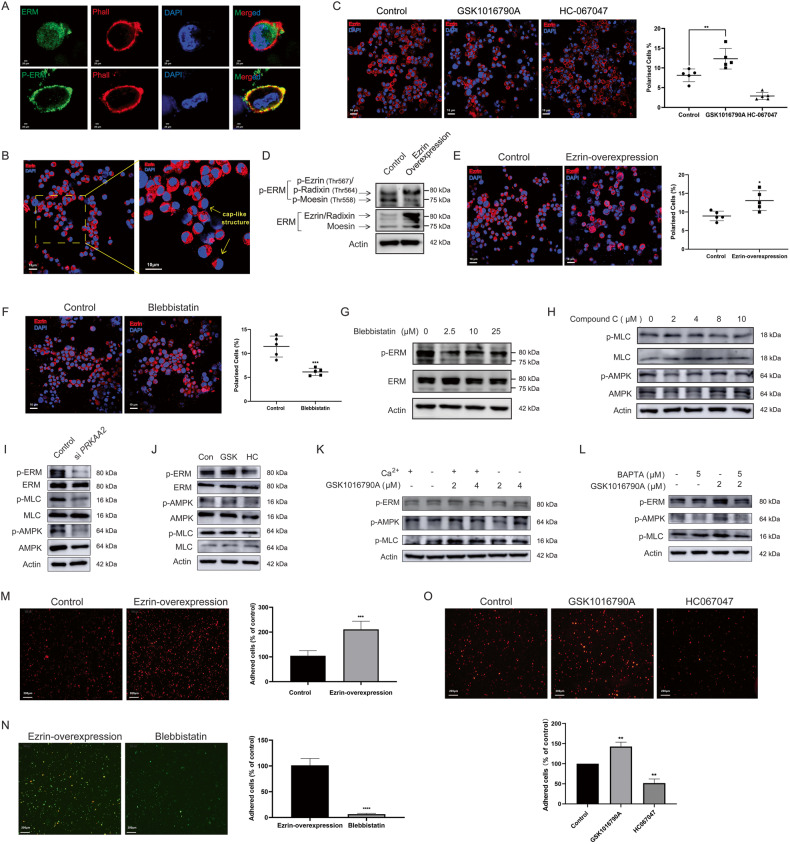
TRPV4 alters sc polarity of HCC cells by regulating Ca^2+^-dependent AMPK/MLC/ERM axis and promotes the adhesion to HUVEC. MHCC-97H cells were cultured in poly-HEMA-coated six-well plate and maintained in suspension. **A** The cap-like structure of suspension-grown MHCC-97H cells stained with phalloidin, ERM or p-ERM antibodies (×100, scale bars: 20 µm). **B** Morphology of cap-like pole (×40, Red: p-ERM, Blue: DAPI). Scale bars: 10 µm. **C** MHCC-97H cells were treated with 8 μM HC067047 or 4 μM GSK1016790A for 12 h. Sc polarity structure was photographed by the confocal microscope (×40, scale bars: 10 µm). The percentage of cells with cap-like pole were quantified. **D**, **E** MHCC-97H cells were stably overexpressed with ezrin-GFP. **D** Protein expression of ERM and p-ERM. **E** Sc polarity structure was photographed by the confocal microscope (×40, scale bars: 10 µm). The percentage of cells with cap-like pole were quantified. **F**, **G** MHCC-97H cells were treated with blebbistatin for 24 h. **F** Sc polarity structure was photographed (2.5 µM blebbistatin, ×40, scale bars: 10 µm). **G** Protein expression of ERM and p-ERM. **H**–**L** Protein expression of ERM, p-ERM, MLC, p-MLC, AMPK and p-AMPK in suspension-grown MHCC-97H cells. **H** Compound C treatment for 24 h. **I** *PRKAA2* knockdown. **J** Treatment of 8 μM HC067047 or 4 μM GSK1016790A for 24 h. **K** Treatment of GSK1016790A for 12 h in either a calcium-containing or calcium-free medium. **L** BAPTA calcium chelator pretreatment lasting 30 min was followed by GSK016790A treatment lasting another 12 h. **M** In vitro adhesion to HUVEC assay. After being labeled with Dil, MHCC-97H cells were incubated for 1 h with HUVEC monolayers. The adhered MHCC-97H cells was photographed by a fluorescence microscope (×4, Red fluorescence: Dil, scale bar: 200 µm). **N** Ezrin-GFP-overexpressed MHCC-97H cell were treated with 2.5 µM blebbistatin for 24 h. The number of adhered ezrin-GFP-overexpressed MHCC-97H cells was photographed and quantified (×4, scale bar: 200 µm). **O** MHCC-97H cells were treated with 4 μM GSK1016790A or 8 μM HC067047 for 24 h. Dil-labeled MHCC-97H cells that were adhered to HUVEC was photographed and quantified (×4, Red fluorescence: Dil, scale bar: 200 µm). Bar, SD. ^*^P < 0.05, ^**^P < 0.01, ^***^P < 0.001 or ^****^P < 0.0001 versus the untreated control.

Single cell (Sc) polarity is a special cytoskeleton structure of cells in liquid phase. We maintained HCC cells in suspension by using a non-adhesive substrate poly-HEMA-coated plate. There was no discernible difference in the survival rate of suspension-grown MHCC-97H cells compared to adherent cells (Supplementary Fig. S3A). Scaffold protein ERM is essential for the formation of sc polarity [11]. As shown in Fig. 2A, both ERM and activated p-ERM protein could co-localize with cytoskeletal protein F-actin tagged by phalloidin probe, and formed a cap-like structure at one pole of single MHCC-97H cells grown in suspension. As a result, this cap-like shape of F-actin or Ezrin (ERM stands for Ezrin-Radixin-Moesin) could be used to indicate sc polarity (Fig. 2B, Supplementary Fig. S3B). In addition to MHCC-97H cells, other suspension-grown HCC cell lines, including HCC-LM3, SMMC-7721, and HepG2 also showed sc polarity (Supplementary Fig. S3C). Next, we investigated the effect of TRPV4 in sc polarity of HCC cells. TRPV4 agonist GSK1016790A increased the fraction of sc polarized cells while TRPV4 inhibitor HC067047 greatly decreased it (Fig. 2C and Supplementary Fig. S3D). When ezrin was persistently overexpressed in MHCC-97H cells, the ratio of cells with sc polarity significantly increased (Fig. 2D, Supplementary Fig. S3E), further demonstrating that ERM was an important regulator of sc polarity.

In the investigations mentioned above, we show that TRPV4 intervenes the AMPK/MLC signal of HCC cells. Myosin activity is regulated by MLC, which affects cortical contractility, and MLC phosphorylation is a major regulatory mechanism for sc polarity [8]. By inhibiting ATPase activity, the myosin motor inhibitor blebbistatin stops the MLC-driven actin–myosin contraction [32]. We discovered that blebbistatin reduced the degree of sc polarization and inhibited the phosphorylation of ERM (Fig. 2F, G and Supplementary Fig. S3F). Moreover, the influence of AMPK on sc polarity was investigated. As shown in Fig. 2H-I, the Compound C-induced activity inhibition of AMPK as well as the siRNA-targeted *PRKAA2* inactivated MLC/ERM signal. Activation of AMPK can be regulated by intracellular Ca^2+^ signal [24]. As predicted, the TRPV4 inhibitor HC067047 markedly reduced the phosphorylation of ERM, MLC, and AMPK in suspension MHCC-97H cells, but the TRPV4 agonist GSK1016790A activated AMPK/MLC/ERM signaling axis (Fig. 2J, Supplementary Fig. S3G). The activation of AMPK/MLC/ERM axis by GSK1016790A was diminished when Ca^2+^ was depleted by utilizing calcium-free culture medium or by administering BAPTA, a cell-permeant chelator highly selective for Ca^2+^ (Fig. 2K, L). TRPV4 therefore regulates sc polarity by influencing Ca^2+^-dependent AMPK/MLC/ERM axis. Previous report demonstrated that sc polarity directly increased the ability of tumor cells to adhere to and exude from vascular endothelial cells [8, 12]. In our study, ezrin overexpression greatly enhanced the number of Dil-stained MHCC-97H cells adhering to HUVEC, but blebbistatin significantly decreased this number (Fig. 2M, N). Undoubtedly, activating or inhibiting TRPV4 increased or prevented HCC cells from adhering to HUVEC, respectively (Fig. 2O).

### The new TRPV4 inhibitor GL-V9 prevents Ca^2+^ influx of HCC cells

We have demonstrated that TRPV4 is a powerful target for metastatic HCC by influencing the migration, invasion, sc polarity and adhesion to vascular endothelial cells. The development of TRPV4 inhibitor will be of great importance in overcoming treatment failure caused by cancer metastasis. It is reported that baicalin and baicalein inhibit calcium influx induced by TRPV4 activation [33, 34]. GL-V9 is a newly synthesized flavonoid compound and derived from wogonin, which has the same nuclear structure as baicalin. So, we investigated the effects of GL-V9 on TRPV4.

**Fig. 3:**
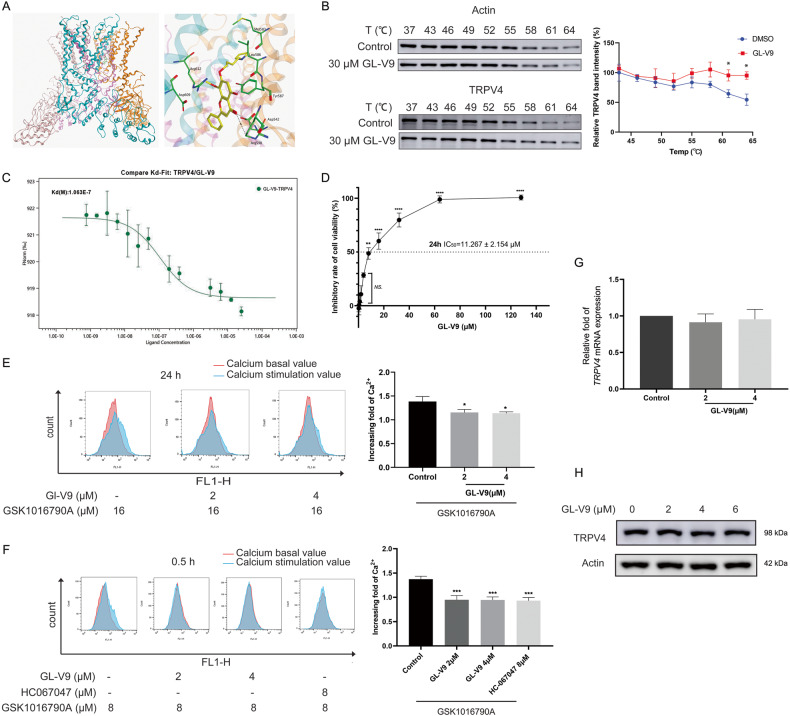
GL-V9 inhibits TRPV4 and prevents Ca^2+^ influx. **A** Computer docking simulation of the crystal structure of human TRPV4 protein in complex with GL-V9. **B** Adherent MHCC-97H cells were treated with 30 μM GL-V9 for 1 h. The temperatures were increased from 37.0 to 64.0 °C during the CETSA assay directed towards TRPV4. TRPV4 protein level was measured by western blot. **C** An MST assay was carried out to analyze the interactions between TRPV4 and GL-V9. **D** GL-V9 was applied to adherent MHCC-97H cells in a range of concentrations (0.25 μM to 128 μM) for 24 h. Cell viability was determined using the MTT test. **E** Adherent MHCC-97H cells were in the pre-treated with GL-V9 for 24 h, incubated with Fluo-4 AM for 0.5 h, and then injected into flow cytometry for 2 min to obtain the baseline value of intracellular calcium ions. The cells were then stimulated with 16 μM GSK1016790A for a brief period of time, and the calcium ion fluorescence intensity was acquired by flow cytometry analysis. **F** Adherent MHCC-97H cells were in the pre-treated with GL-V9 or HC067047 for 0.5 h. The intracellular calcium was then determined using Fluo-4 AM as described above. **G**, **H** The mRNA and protein expressions of TRPV4 were assessed upon GL-V9 treatment for 24 h. Bar, SD. ^*^P < 0.05, ^**^P < 0.01, ^***^P < 0.001 or ^****^P < 0.0001 versus the untreated control.

Firstly, a molecular docking study was used to determine the binding between GL-V9 and TRPV4. At the lowest energy conformation, GL-V9 occupied the active pocket composed by Asp542, Met583, Leu586, Tyr587, Arg590, and Arg612 amino acid residues of TRPV4 (Fig. 3A). Among them, Asp542, Tyr587, and Arg590 were the three most important amino acid residues. The carbonyl group in the flavonoid skeleton formed hydrogen bonds with Arg590 and Asp542, the 5-hydroxyl formed hydrogen bonding interactions with Leu586, the pyrrole ring introduced at the 7-position could penetrate deep into the solvent region. Then we applied CETSA assay to test the interaction between GL-V9 and TRPV4 protein. As shown in Fig. 3B, in GL-V9-treated group, TRPV4 protein started to be degraded at 64 °C instead of 58 °C in control group, indicating that GL-V9 interacts with TRPV4 protein and enhanced its thermal stabilization. Next, an MST assay was carried out to investigate whether GL-V9 and TRPV4 directly interact. The result showed that GL-V9 bound to TRPV4 protein with a dissociation constant (Kd) value of 0.106 μM (Fig. 3C).

The IC50 value for 24 h GL-V9 treatment in adherent MHCC-97H cells was 11.267 ± 2.154 μM, and GL-V9 treatment lower than 4 μM had no discernible effect on cell growth (Fig. 3D). To examine the calcium ion flow in the cytoplasm, we used the calcium ion probe Fluo-4 AM. In adherent MHCC-97H cells, TRPV4 agonist GSK1016790A triggered Ca^2+^ influx and elevated intracellular Ca^2+^ level, and the pre-treatment with GL-V9 for 24 h blocked the calcium ion influx induced by GSK1016790A (Fig. 3E). Then we employed the pre-treatment of GL-V9 for 0.5 h, which produces short-term pharmaceutical impact and little influences on transcription, to further investigate whether the blocking of calcium influx by GL-V9 was caused by the direct impact on transient alteration of TRPV4 channel. TRPV4 inhibitor HC067047 was the positive control. Pre-incubation of GL-V9 or HC067047 for 0.5 h both substantially reduced the calcium influx brought on by GSK1016790A (Fig. 3F). Additionally, the mRNA and protein expressions of TRPV4 were unaffected by the low concentration of GL-V9 (Fig. 3G, H). All the above results indicate that GL-V9 inhibits TRPV4, prevents Ca^2+^ influx, and reduces intracellular Ca^2+^ signaling in MHCC-97H cells.

### GL-V9 suppresses the migration, invasion, and sc polarity of HCC cells by inhibiting TRPV4

**Fig. 4:**
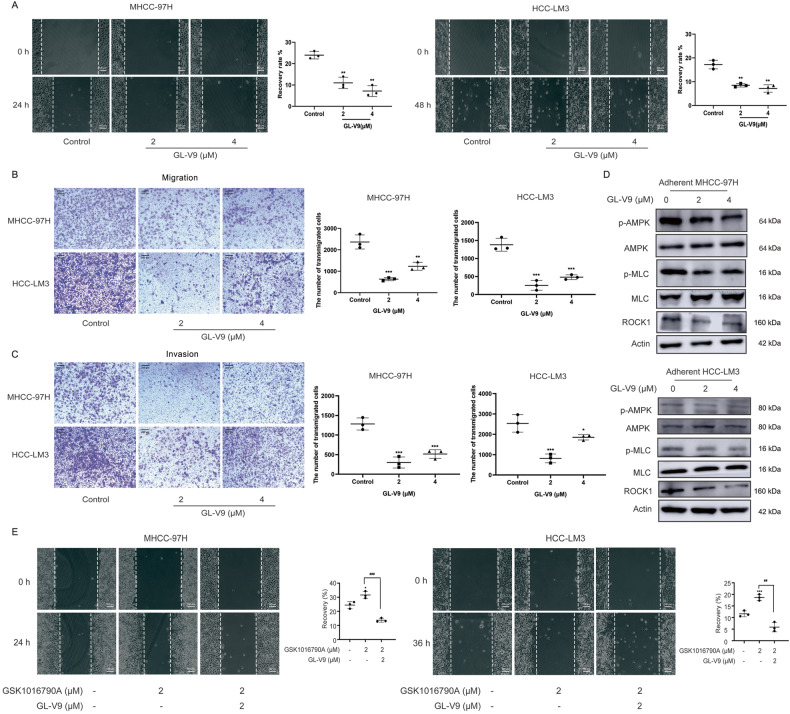
GL-V9 prevents the migration and invasion of HCC cells by inhibiting TRPV4 and regulating cytoskeleton-associated proteins. **A** MHCC-97H and HCC-LM3 cells were treated with GL-V9 for 24 h or 48 h, respectively. Wound healing assay was done and scratch healing rate was quantified. Scale bar:100 μm. **B**–**D** MHCC-97H or HCC-LM3 cells were treated with GL-V9 for 24 h. **B** Transwell migration assay and **C** transwell invasion assay were detected and the number of transmigrated cells were quantified. Scale bar: 200 μm. **D** Cytoskeleton-related proteins were tested by western blot. **E** The MHCC-97H or HCC-LM3 cells were pretreated with GL-V9 for 12 h, respectively, then co-stimulated with GSK1016790A for another 12 h or 36 h, respectively. Wound healing assay was done and scratch healing rate was quantified. Scale bar: 100 μm. This experiment was performed in parallel with that of Fig. 1C. In Fig. 1C and 4E, the pictures for the control group and the GSK1016790A alone group were identical. *Bar*, SD. ^*^P < 0.05, ^**^P < 0.01, ^***^P < 0.001 or ^****^P < 0.0001 versus the untreated control. ^*##*^*P* < 0.01, ^*###*^*P* < 0.001 compared between groups.

Previous studies show that GL-V9 suppresses invasion and migration of human colorectal cancer cells by inhibiting PI3K/AKT and MMP-2/9 signaling [35], and inhibits the anchorage-independent growth of breast cancer cells [36]. Treatment with GL-V9 in the absence of proliferation inhibitory concentrations in MHCC-97H and HCC-LM3 cells (Fig. 3D and Supplementary Fig. S4A, B), significantly reduced the migration and invasion (Fig. 4A–C), and the phosphorylation of AMPK, MLC and ROCK1 protein (Fig. 4D). GSK1016790A promoted the migration of HCC cells, but GL-V9 reversed this effect (Fig. 4E).

**Fig. 5:**
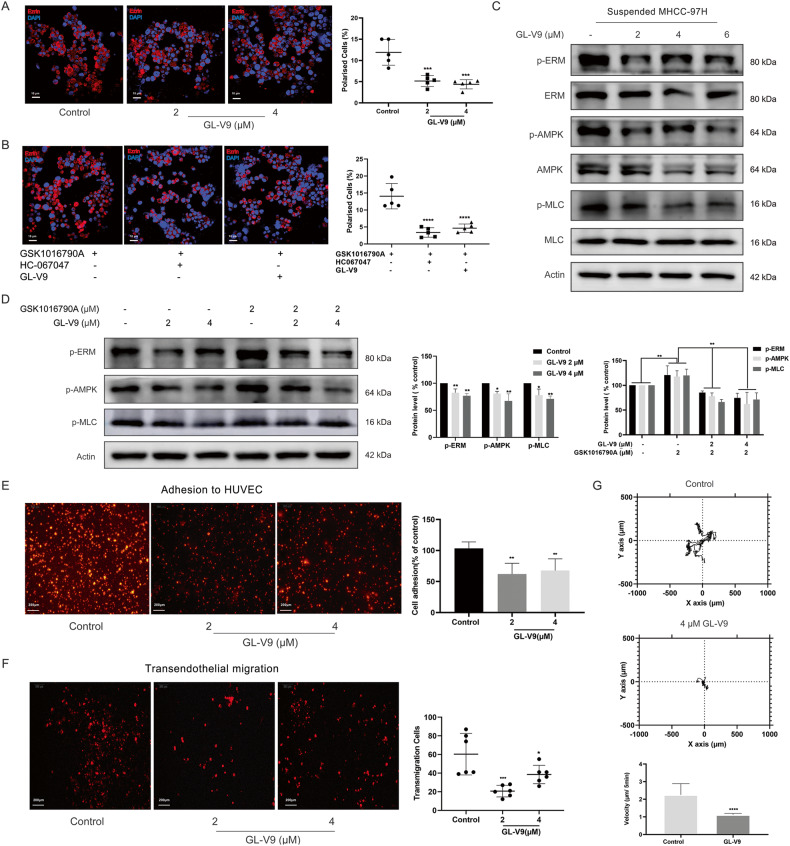
GL-V9 remodels the sc polarity by inhibiting TRPV4-regulated AMPK/MLC/ERM axis. **A**, **B** Morphology of suspension-grown MHCC-97H cells with cap-like pole (×40, Red: p-ERM, Blue: DAPI). Scale bars: 10 µm. The percentage of cells with cap-like pole were quantified. **A** GL-V9 treatment for 24 h. **B** Pretreatment of 2 μM GL-V9 or 8 μM HC067047 for 12 h, followed by 4 μM GSK1016790A treatment for another 12 h. **C**, **D** Protein expressions involved in AMPK/MLC/ERM axis. **C** GL-V9 treatment for 24 h. **D** Cells were pre-treated with GL-V9 for 12 h, then co-treated with GSK1016790A for another 12 h. **E** In vitro adhesion to HUVEC assay. Monolayer HUVEC cells were incubated with MHCC-97H cells for 1 h which were previously suspension-grown, treated with GL-V9 for 24 h, then labeled with Dil. Dil-labeled MHCC-97H cells that were adhered to HUVEC was photographed and quantified (×4, Red fluorescence: Dil, scale bar: 200 µm). **F** In vitro transendothelial cell migration assay. The extravasation migration through HUVEC monolayer of Dil-labeled MHCC-97H cells were photographed and quantified (×4, Red: Dil, scale bar: 200 µm). **G** Pretreated cells were cultured in suspension, and the living cell workstation monitored single cell motility for 24 h. Cell movement distance and speed were quantified. Bar, SD. ^*^P < 0.05, ^**^P < 0.01, ^***^P < 0.001 or ^****^P < 0.0001.

On the other hand, GL-V9 treatment lower than 4 μM had no discernible effects on the growth of suspension-grown MHCC-97H cells (Supplementary Fig. S4C, D). Being identical to HC06704, GL-V9 considerably reduced the proportion of sc polarized cells (Fig. 5A and Supplementary Fig. S4E), and diminished the promoting effect of GSK1016790A (Fig. 5B, Supplementary Fig. S4F), being the same as HC06704. GL-V9 also inactivated AMPK/MLC/ERM signaling axis of suspension-grown HCC cells (Fig. 5C and Supplementary Fig. S4G), and abolished the activating effects of GSK1016790A on AMPK/MLC/ERM axis (Fig. 5D and Supplementary Fig. S4H). Additional research revealed that GL-V9 inhibited the adhesion to HUVEC and the transendothelial migration of suspension-grown MHCC-97H cells (Fig. 5E, F). It was well known that the movement pattern of sc polarity is similar to the movement of amoeba [15, 37]. We used the live cell workstation to observe the rapid amoebic movement trajectory of suspension-grown MHCC-97H cells. The distance and speed of cell movement both clearly decreased after exposure to GL-V9 (Fig. 5G, Supplementary video 1, and Supplementary video 2). As a result, the TRPV4 inhibitor GL-V9 inactivates the AMPK/MLC/ERM axis of suspended HCC cells, suppressing sc polarity, adhesion to HUVEC and transendothelial migration.

### GL-V9 reduces the adhesion of HCC cells to blood vessel and suppresses metastasis in vivo

**Fig. 6:**
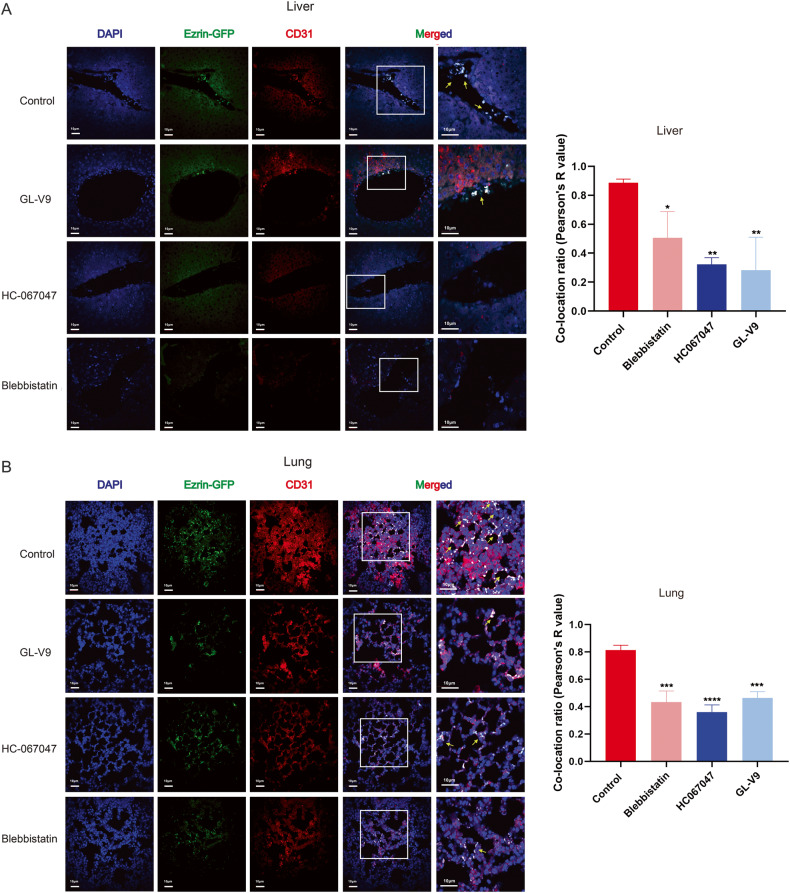
GL-V9 reduces the adhesion of HCC cells to blood vessel in vivo. Suspended MHCC-97H cells overexpressed ezrin-GFP were pre-treated with 2 μM GL-V9, 8 μM HC-067047, or 2.5 μM blebbistatin for 1 h. Nude mice (n = 3 for each group) were injected with 5 × 10^6^ pre-treated MHCC-97H cells through the tail vein, and sacrificed 2 h later. The tissue slices of the livers (**A**) and lungs (**B**) were used for immunofluorescence assay (×40, scale bars: 10 µm). Ezrin-GFP of MHCC-97H cells and CD31 of vascular endothelial cells were co-localized and the degree of co-localization was represented by Pearson’s R value. Bar, SD. ^*^P < 0.05, ^**^P < 0.01, ^***^P < 0.001, or ^****^P < 0.0001.

In further study, we investigated how GL-V9 may affect the adhesion to HUVEC and metastasis of HCC in vivo. We injected pretreated MHCC-97H cells into nude mouse through the tail vein and observed the adhesion of HCC cells to the blood vessels in the livers and lungs. As shown in Fig. 6, pretreatment with GL-V9 greatly reduced the adhesion of MHCC-97H cells to the blood vessel marked by CD31 in the livers and lungs, while HC-067047 and blebbistatin also had a comparable inhibitory effect. According to this finding, the inhibition of TRPV4 and the prevention of sc polarity both hinder the adhesion of HCC cells to vascular endothelial cells.

**Fig. 7:**
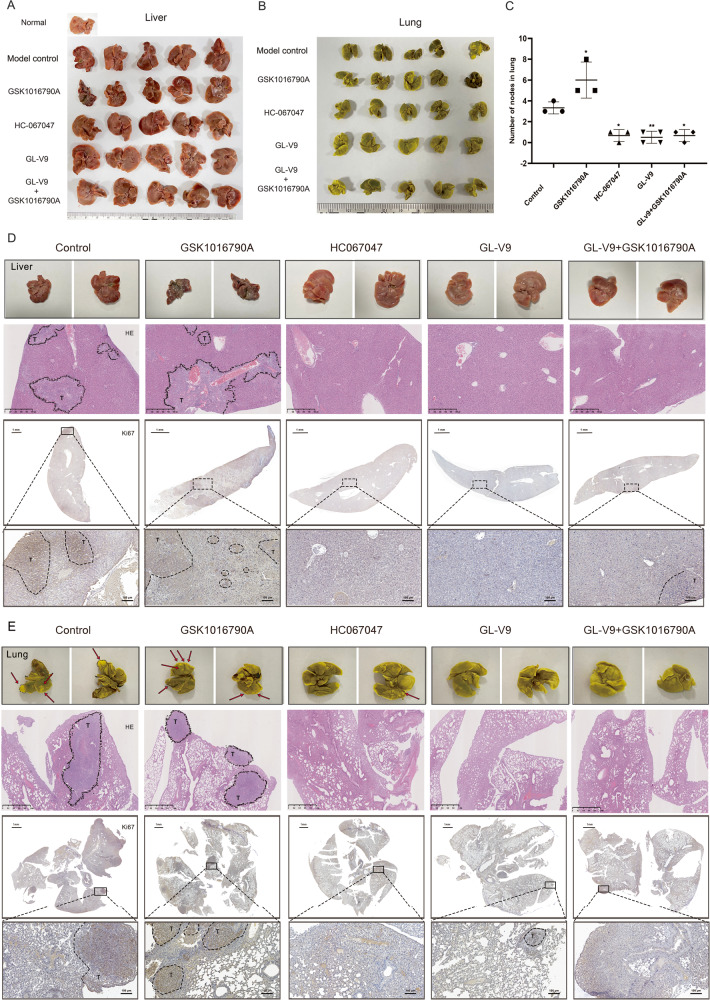
GL-V9 suppresses the liver and lung metastases of MHCC-97H cells in vivo. The metastatic HCC model was established via tail-vein injection of 7 × 10^6^ MHCC-97H cells per mouse (*n* = 5). **A**, **B** The photo of liver and lung metastases. **C** Quantification of pulmonary metastatic nodules in lung. Bar, SD. ^*^P < 0.05, ^**^P < 0.01. **D** Photos of a representative selection from each group were enlarged and displayed on the front and back. Arrows indicate visible metastatic nodules. HE staining of the liver and lungs tissue (×20, scale bars ^liver^: 500 µm, scale bars ^lung^: 1 mm). IHC assay for Ki67 of the liver and lung tissue (scale bars ^full view^: 1 mm; ×20, scale bars ^local^: 100 µm). “T” was referred to the metastatic location.

**Fig. 8:**
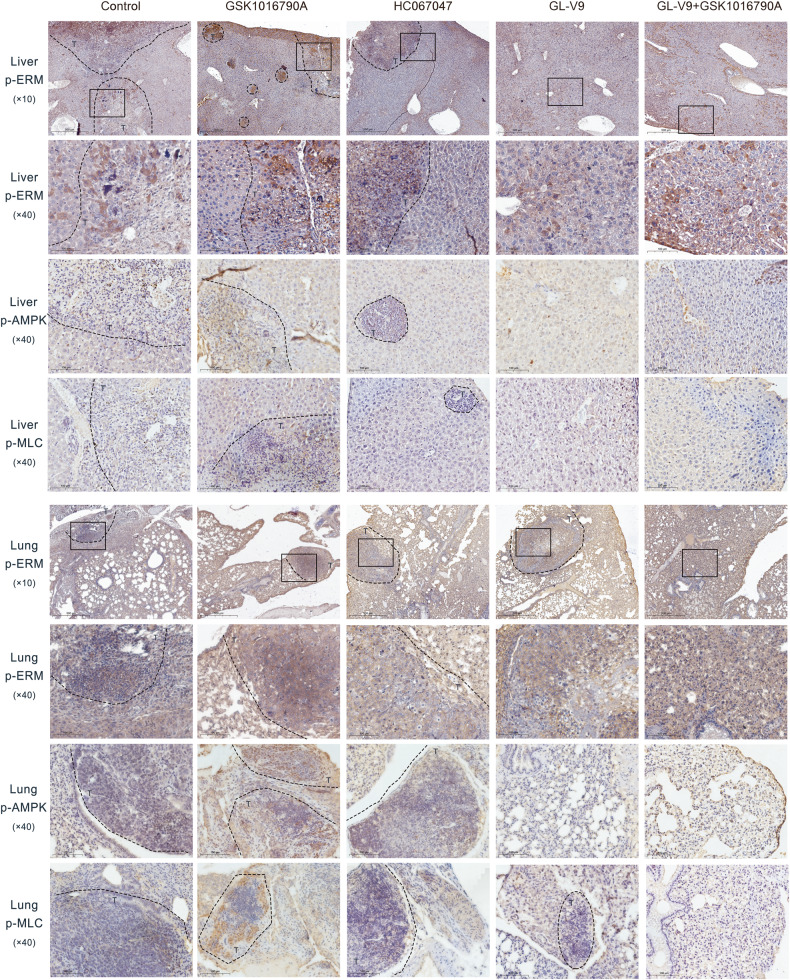
IHC assay for p-ERM, p-MLC, p-AMPK protein in liver and lung tissue of the metastatic HCC model mice. ×10, scale bars: 500 µm; ×40, scale bars: 100 µm. “T” was referred to the metastatic location.

Following that, we evaluated the pharmacological effects of GL-V9 on the metastasis using the metastatic HCC model by tail vein injection of MHCC-97H cells. The results demonstrated that TRPV4 agonist GSK1016790A promoted the liver metastasis of MHCC-97H cells (Fig. 7A), and increased lung metastatic locations (Fig. 7B, C). Instead, the liver and lung metastases were dramatically reduced by the TRPV4 inhibitors GL-V9 and HC067047. The rise in liver and lung metastases brought on by GSK1016790A was also prevented by GL-V9 co-treatment. Studies of HE staining and immunohistochemistry assay revealed that the liver and lung metastatic sites exhibited deeper nuclear staining, more aberrant cell aggregation and elevated levels of Ki67 (Fig. 7D, E). Additionally, GL-V9 and HC-067047 treatment groups showed lower level of p-ERM, p-MLC, and p-AMPK than the model control and GSK1016790A treatment groups (Fig. 8). Due to TRPV4 activation mediates the influx of calcium ions, it may result in heart and kidney damage, particularly in this lengthy experiment. Therefore, we assessed the toxicity of TRPV4 inhibitors GL-V9, HC-067047 and TRPV4 agonist GSK1016790A. The results of HE staining revealed that no significant damages were observed in the hearts and kidneys (Supplementary Fig. S5A). Also, mice with tumors lost body weight when exposed to the GSK1016790A, but not when exposed to GL-V9 or HC-067047 (Supplementary Fig. S5B). These findings demonstrate that TRPV4 activation promotes the metastasis of HCC in vivo, and GL-V9 effectively prevents HCC metastasis by inhibiting AMPK/MLC/ERM pathway implicated in sc polarity.

## Discussion

**Fig. 9:**
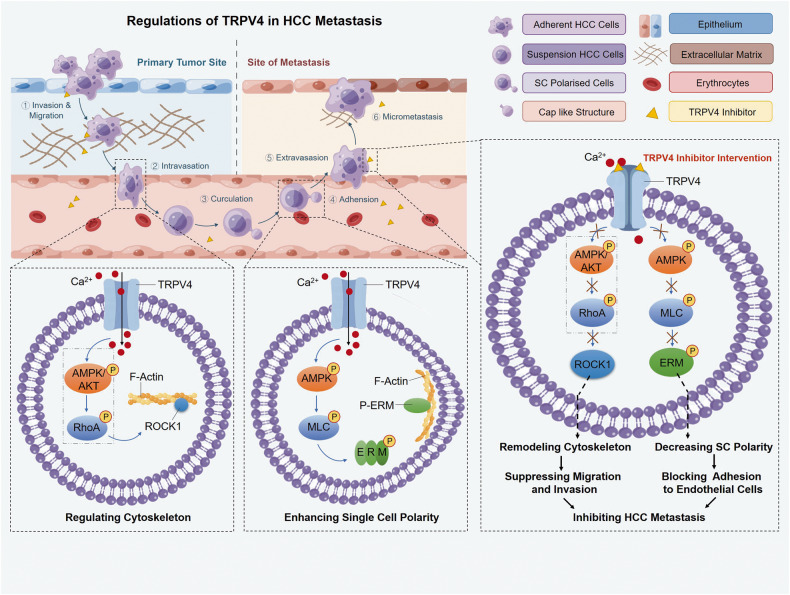
Schematic representation of TRPV4 regulations in the metastasis of HCC. Activation of Ca^2+^ channel TRPV4 modifies ROCK1 to promote the migration and invasion of HCC cells. Meanwhile, TRPV4 activation affects sc polarity by upregulating Ca^2+^-dependent AMPK/MLC/ERM pathway, which increases the adhesion of metastatic HCC cells to blood vessel. Pharmacological inhibition of TRPV4 effectively suppresses HCC metastasis by intervening cell migration, invasion and sc polarity. The contents implicated in AMPK or AKT/RhoA/ROCK1 pathway shown in gray box has been proved by previous reports. Part of the drawing elements was from Figdraw (www.Fogdraw.com).

HCC is the leading cause of cancer-related death. Most advanced HCC patients do not meet the indications for surgery, and can only undergo systematic chemotherapy and molecular targeted therapy. Unfortunately, many drug therapies applied for HCC fail to prolong patient survival due to the heterogeneity of tumor cells, drug resistance and metastasis [38–40]. Because of the abundant of vascular system in liver, HCC cells are more likely to enter the circulatory system, and overcome the fluid shear as well as immune system [41]. Thus, targeting the circulating tumor cells will be a potential strategy to prevent HCC metastasis [42]. In this study, we investigated TRPV4 regulations in HCC metastasis. TRPV4 modifies cytoskeleton-related protein to promote the migration and invasion of HCC cells, and alters sc polarity via activating Ca^2+^-dependent AMPK/MLC/ERM pathway, which increases the adhesion of metastatic HCC cells to blood vessels. Consequently, pharmacological inhibition of TRPV4 effectively prevents the metastasis of HCC by inhibiting cell migration, invasion, and sc polarity (Fig. 9).

Many types of cancer, including liver cancer, breast cancer, esophageal squamous cell carcinoma, gastric cancer cells, and colon cancer, maintain high expression and activated characteristics of TRPV4 [22, 43–47]. Recent studies have revealed that TRPV4 and intracellular calcium signaling are crucial to cancer metastasis [48]. TRPV4 can affect tumor angiogenesis and inflammatory microenvironment [49, 50], intervene in the cytoskeleton-related proteins including RhoA, CDC42, and Rac1 [51, 52], and promote cancer cells EMT. In breast cancer-derived endothelial cells, TRPV4 co-localizes with actin, regulates filopodia and lamellipodia changes, and improves cell motility [53]. In the circulatory system, when metastatic tumor cells are subjected to fluid shear force, TRPV4 channel is opened and changes cytoskeleton, thereby promoting the extravasation of cancer cells [54, 55]. Here, we discover that TRPV4 is not only crucial for cytoskeletal regulation, but also act as a critical modulator of sc polarity. Interestingly, our results showed neither activation nor inhibition of TRPV4 significantly changed the EMT marker expression in HCC. We speculate that the distinct role of TRPV4 in EMT may be due to the different metastatic propensity of types of cancer cells. Therefore, there is still a need for extensive study on the roles of TRPV4 in metastasis.

Sc polarity is a basal cell cortical polarity that maintains only in the liquid phase, which increases the resistance to liquids of anchorage-independent cells, enhances adhesion ability to endothelial cells and promotes metastasis [8, 12]. ERM and F-actin proteins work together to form a dot or cap structure in the cellular cortex. *TRPV4* knockdown in breast cancer reduces the cellular blistering motility and flexibility by regulating ERM phosphorylation, contributing to the processes of tumor extravasation and transcellular migration [56]. Our study demonstrates that TRPV4 promotes sc polarity via upregulating Ca^2+^-dependent AMPK/MLC/ERM pathway. Cascade activation of AMPK/MLC regulates the phosphorylation of ERM. According to previous literatures, RhoA-ROCK1 also affects ERM phosphorylation [57, 58]. ROCK1 serves an important function in cell migration and invasion in neoplasms [59], and is the classic downstream effector of the small GTPase RhoA [60], the activation of which is mediated by AKT phosphorylation [61, 62]. Additionally, AMPK phosphorylates RhoA and affects ROCK activity to mediate the cytoskeleton remodeling [63]. Recently, ROCK1 is found as a novel Rac1 effector [64], and TRPV4 targets the AKT for phosphorylation, promotes migration and invasion through AKT/Rac1 signaling in glioma [65]. Therefore, TRPV4 control RhoA/ROCK1 pathway by activating AKT and AMPK, and contribute to the migration as well as sc polarity of HCC cells.

A broad range of abnormal calcium signaling dynamics are linked to cancer progression through the capacity to sense and modify mechanically altered microenvironment [66, 67], the association with mechanosensitive Rho GTPases and actin cytoskeleton [68], as well as the role in angiogenesis [53, 69] and cell proliferation [70]. A typical T-type Ca^2+^ channel blockers named mibefradil, previously used for cardiovascular disease, are currently undergoing clinical trials for glioblastoma multiforme [71, 72]. The TRPV6 inhibitor SOR-C13 is already in Phase I clinical trials in patients with advanced solid cancers and is approved as an orphan drug for pancreatic and ovarian cancers [73]. TRPV4 has primarily been studied as a target for cardiovascular disease treatment [74, 75]. Our studies suggest that TRPV4 inhibition effectively prevents HCC metastasis. Thodeti et al. also demonstrate TRPV4 channels to be critical regulators of tumor angiogenesis and represent a novel target for anti-angiogenic and vascular normalization therapies [69, 76]. Unfortunately, TRPV4 regulators have not yet been used in cancer therapy clinics. Preclinical investigations have shown that TRPV4 agonist GSK1016790A inhibits breast cancer cell growth by calcium overload-induced apoptosis [77]. TRPV4 inhibitors further have the potential to treat certain cancers with high safety, as demonstrated by the success of a Phase II clinical study involving the TRPV4 inhibitor GSK2798745 in patients with congestive heart failure [74]. In our studies, TRPV4 inhibitor GL-V9 exerts strong anti-metastatic efficiency in HCC by inactivating both AMPK and AKT-related pathway. Many previous studies have also demonstrated that GL-V9 inactivates AKT signal in types of cancer [35, 78–80]. Similar to the pharmacological components extracted from Scutellaria baicalensis [81, 82], GL-V9 achieves the anticancer effects by regulating multiple pathways. Recent studies suggest that GL-V9 promotes apoptosis of senescent cancer cells by alkalinizing lysosomes and regulating ROS levels [83]. Compared to those natural flavonoids like wogonin, the derivative GL-V9 is greatly improved in the water solubility and bioavailability through chemical modification, enhancing its anticancer effects and druggability [84]. As a result, GL-V9 is a potential drug-candidate for metastatic HCC.

Our research suggests that TRPV4 is a promising therapeutic target for preventing cancer metastasis. Activation of TRPV4 remodels the cytoskeleton by regulating ROCK1 and promotes sc polarity via AMPK/MLC/ERM axis, thereby affecting the migration, invasion and adhesion to blood vessel of metastatic cancer cells. Importantly, pre-clinical studies have shown that TRPV4 inhibitor GL-V9 has a potent anti-metastatic effect and low toxicity. Clinical research will need to assess the therapeutic potential of inhibiting TRPV4 and sc polarity to target cancer metastasis.

## Supplementary information

Supplementary Figures

Supplementary Tables

Supplementary Materials and Methods

Supplementary Vedio 1 Movement of alive MHCC-97H cells without treatment

Supplementary Vedio 2 Movement of alive MHCC-97H cells upon GL-V9 treatment

Reproducibility checklist

## Data Availability

The data that support the findings of this study are available from the corresponding author upon reasonable request.
