# Supplementary material for: Inhibition of TRPV4 remodels single cell polarity and suppresses the metastasis of hepatocellular carcinoma
Source: Cell Death Dis. 2023 Jun 28;14(6):379. doi: 10.1038/s41419-023-05903-z (PMC10300155; doi:10.1038/s41419-023-05903-z)
Supplement: Supplementary file 1 — Supplementary Figures [file 41419_2023_5903_MOESM1_ESM.docx]

**Supplementary Figures**


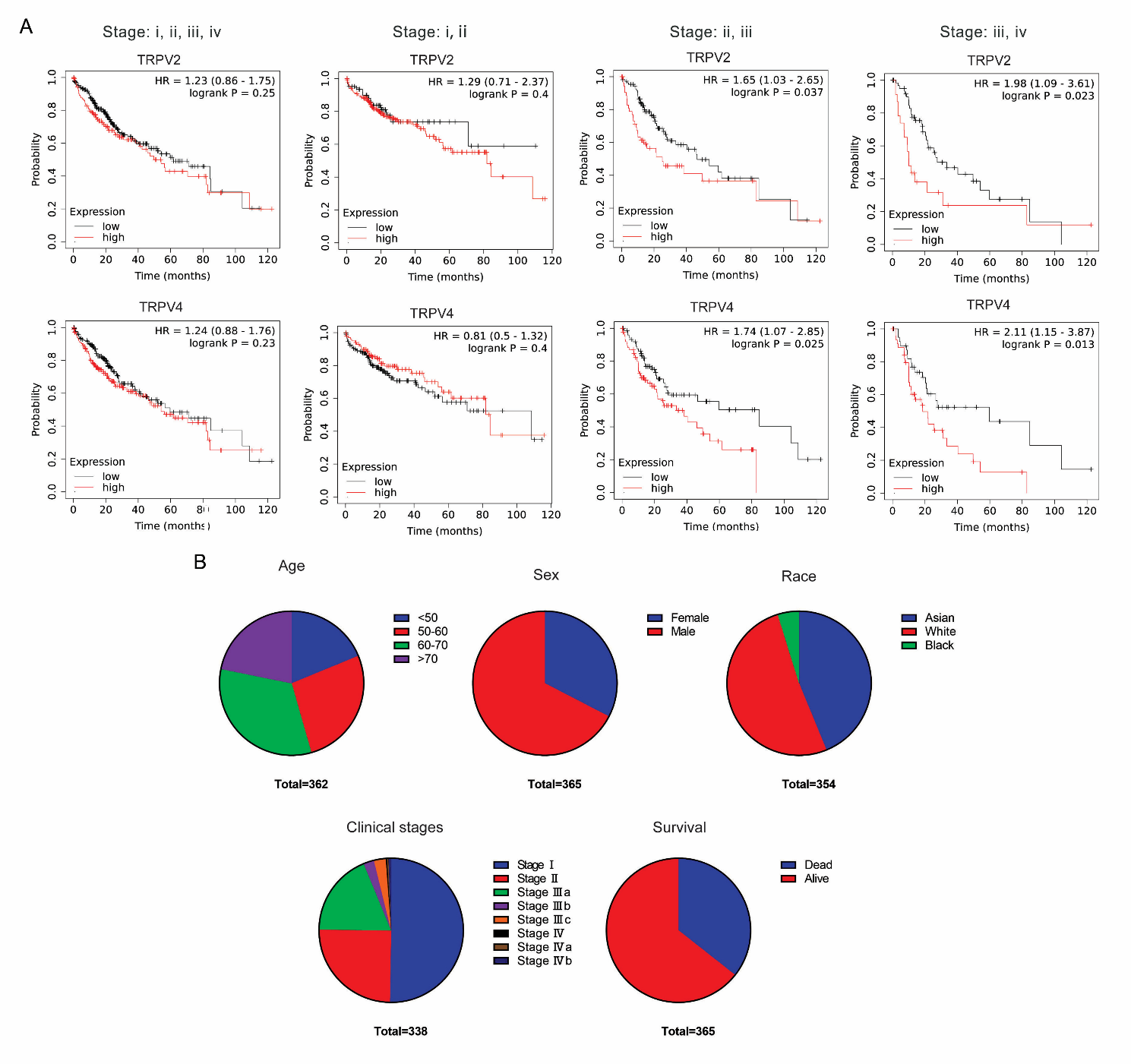


**Supplementary Figure S1** (**A**) Kaplan-Meier Plotter analysis of survival curves in patients with hepatocellular carcinoma expressing *TRPV2* or *TRPV4*. In analyses of Kaplan-Meier curves with a log-rank test, *P* value < 0.05 was considered significant. (**B**) Clinical traits of hepatocellular carcinoma patients who express *TRPV2* or *TRPV4*.


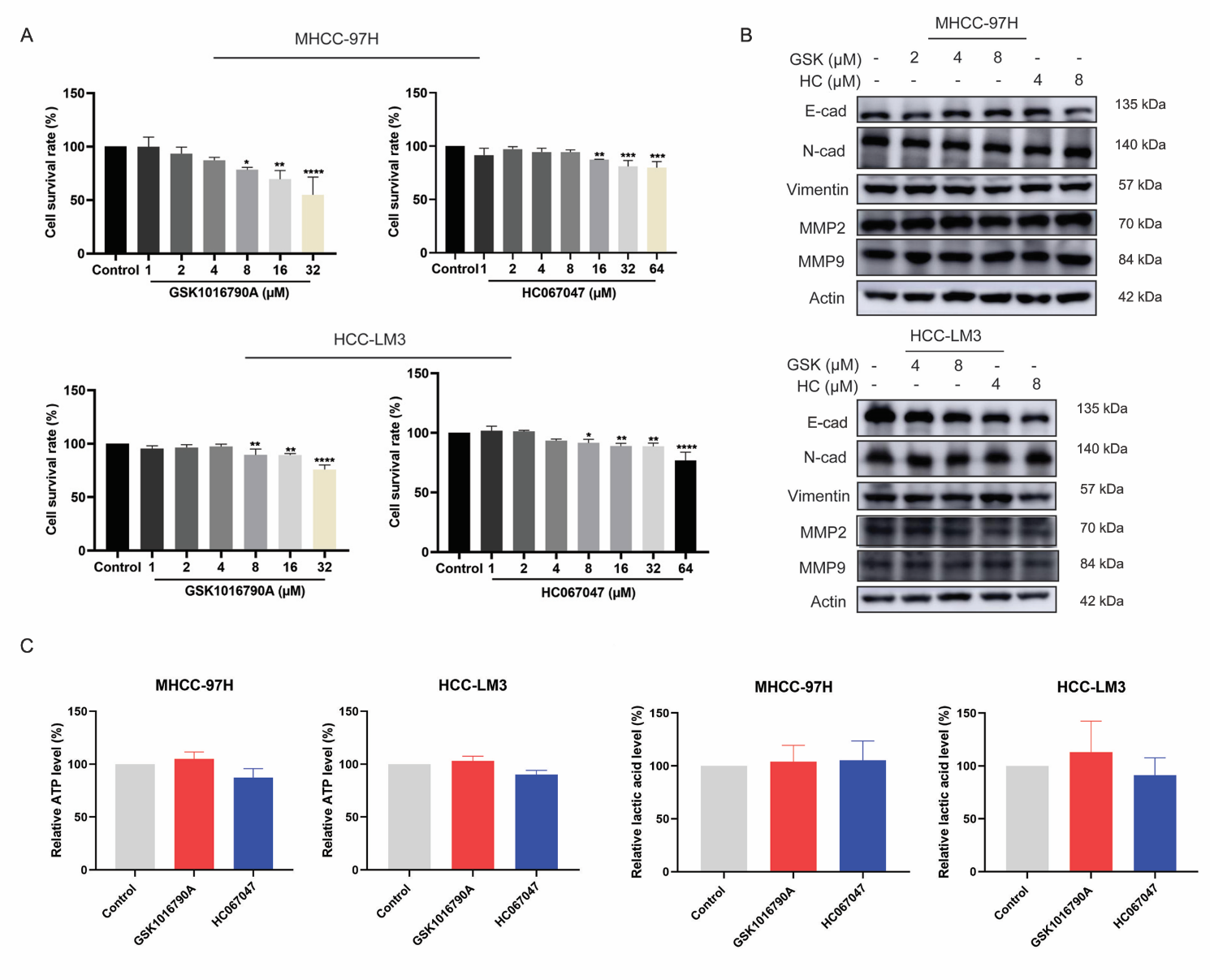


**Supplementary Figure S2** (**A**) MTT assay of MHCC-97H or HCC-LM3 cells upon the treatment of GSK1016790A and HC-067047 for 24 h or 48 h, respectively. (**B, C**) MHCC-97H or HCC-LM3 cells were treated with GSK1016790A or HC067047 for 24 h. (**B**) Expressions of EMT-related proteins and MMP2, MMP9 were measured by western blot. (**C**) ATP levels and lactate generation were assayed. *Bar*, SD. *^*^P*< 0.05, *^**^P*< 0.01, *^***^P*<0.001 or *^****^P*<0.0001 versus the untreated control.


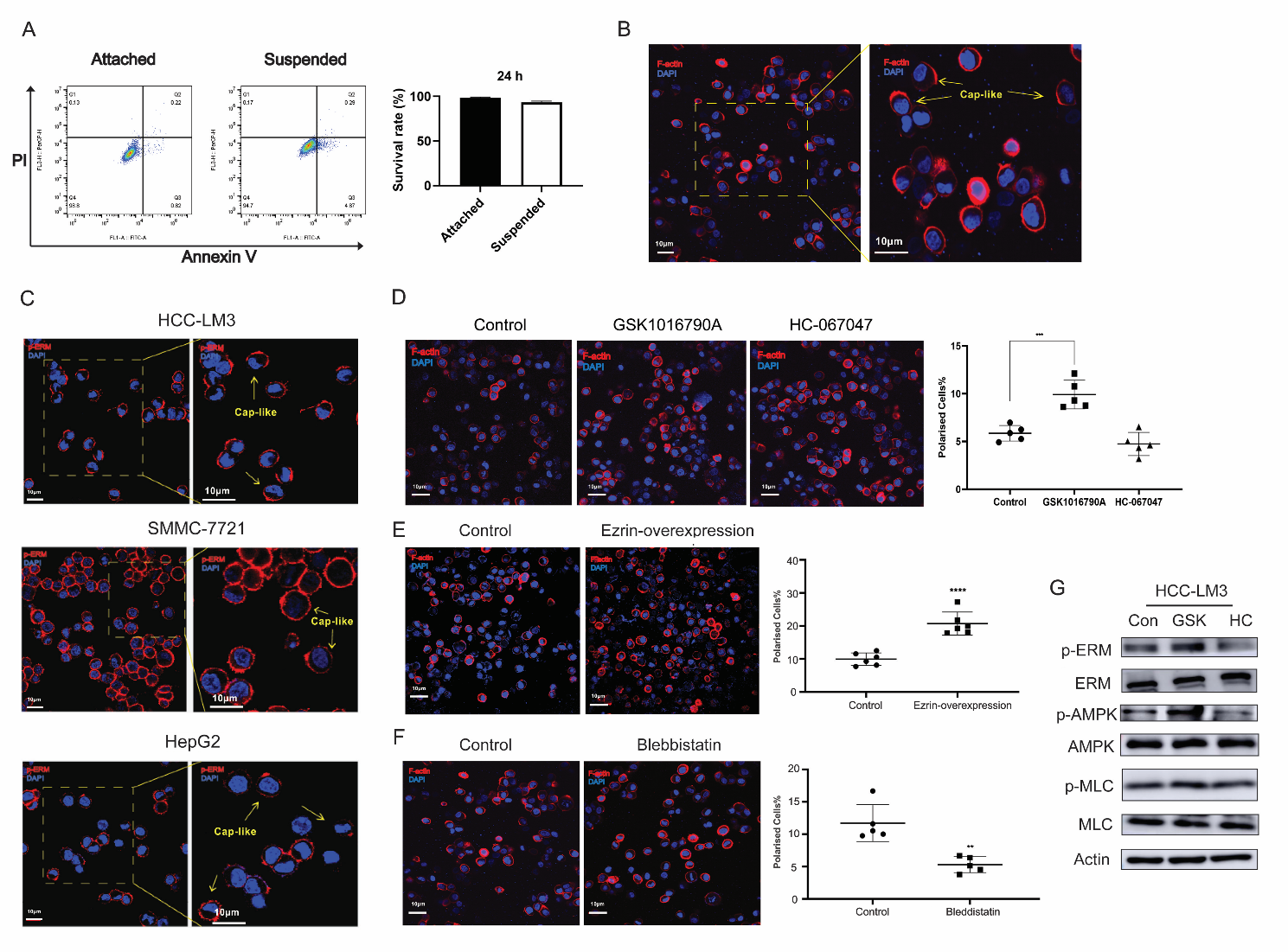


**Supplementary Figure S3** (**A**) The cell survival rate of MHCC-97H cells was assessed using an Annexin-V/PI staining assay after the cells were cultured in adhered and suspended forms for 24 h. (**B**) Morphology of suspension-grown MHCC-97H cells with cap-like pole (40×, Red: F-actin, Blue: DAPI). Scale bars: 10 µm. (**C**) Morphology of suspension-grown HCC-LM3, SMMC-7721 or HepG2 cells with cap-like pole (40×, Red: F-actin, Blue: DAPI, scale bars: 10 µm). (**D-F**) Sc polarity structure of treated suspension-grown MHCC-97H was photographed by the confocal microscope (40×, Red: F-actin, Blue: DAPI, scale bars: 10 µm). The percentage of cells with cap-like pole were quantified. (**D**) Cells were treated with 8 μM HC067047 or 4 μM GSK1016790A for 12 h. (**E**) Cells were overexpressed with ezrin-GFP. (**F**) Cells were treated with 2.5 µM blebbistatin for 24 h. (**G**) Suspension-grown HCC-LM3 cells were treated with 4 μM GSK1016790A and 4 μM HC067047 for 12 h. Protein expression involving in AMPK/MLC/ERM axis were detected. *Bar*, SD. *^*^P*< 0.05, *^**^P*< 0.01, *^***^P*<0.001 or *^****^P*<0.0001 versus the untreated control.


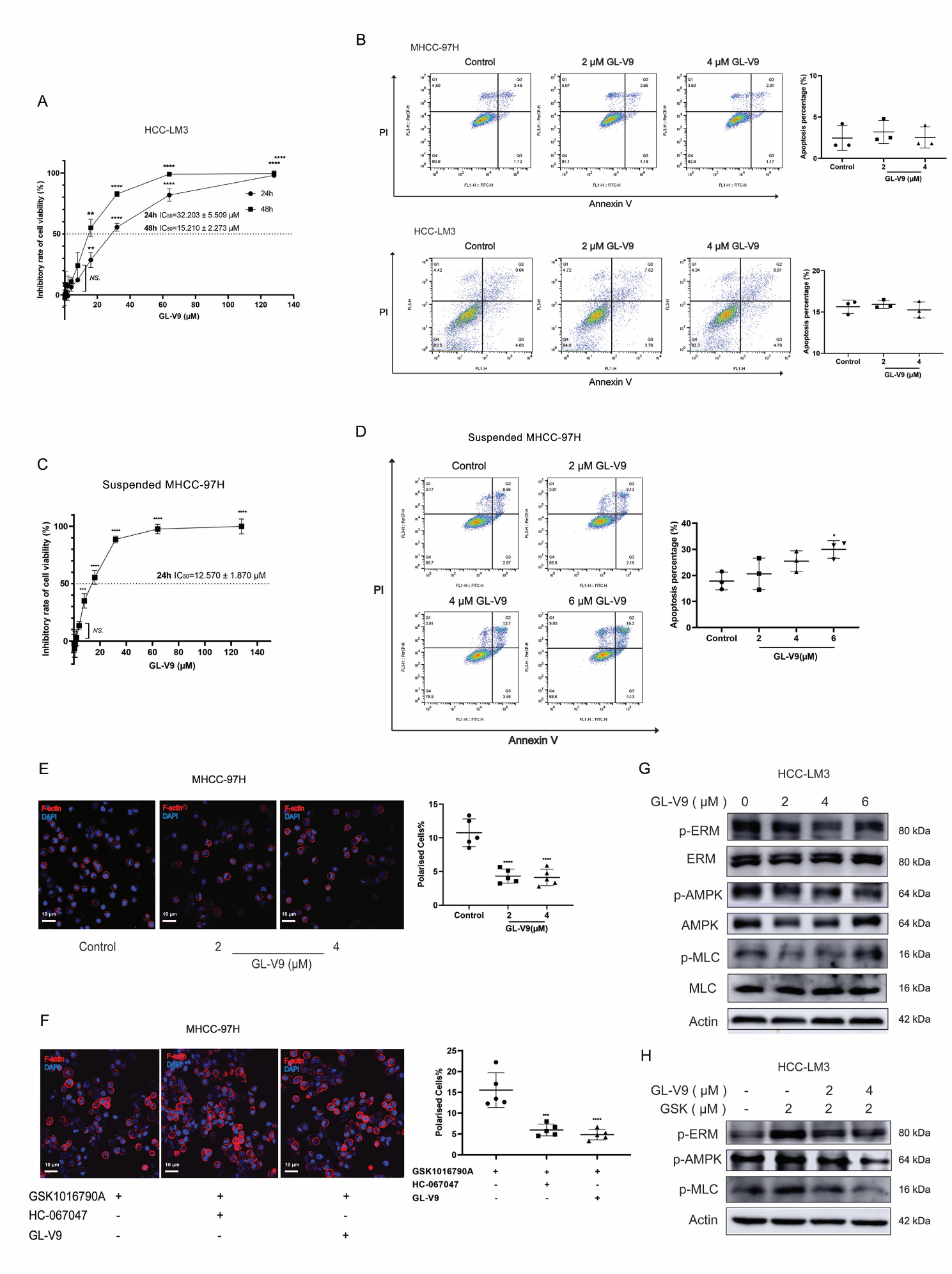


**Supplementary Figure S4** (**A**) MTT assay of adherent HCC-LM3 cells that were treated with gradient concentrations of GL-V9 for 24 h or 48 h. (**B**) Annexin-V/PI double-staining assay of adherent HCC cells. (**C, D**) MHCC-97H cells were cultured in poly-HEMA-coated six-well plate and maintained in suspension. (**C, D**) CCK-8 assay and Annexin-V/PI double-staining assay of suspension-grown MHCC-97H cells that were treated with gradient concentrations of GL-V9 for 24 h. (**E, F**) Morphology of suspended MHCC-97H cells with cap-like pole (40×, Red: F-actin, Blue: DAPI). Scale bars: 10 µm. The percentage of cells with cap-like pole were quantified. (**E**) GL-V9 treatment for 24 h. (**F**) Pretreatment of 2 μM GL-V9 or 8 μM HC067047 for 12 h, then followed by 4 μM GSK1016790A co-treatment for a further 12 h. (**G, H**) Protein expressions involved in AMPK/MLC/ERM axis of suspended HCC-LM3 cells were assayed. (**G**) GL-V9 treatment for 24 h. (**H**) Pretreatment of GL-V9 for 12 h followed by the co-treatment with GSK1016790A for a further 12 h. *Bar*, SD. *^*^P*< 0.05, *^**^P*< 0.01, *^***^P*<0.001 or *^****^P*<0.0001 versus the untreated control. “*NS.*” means no significance.


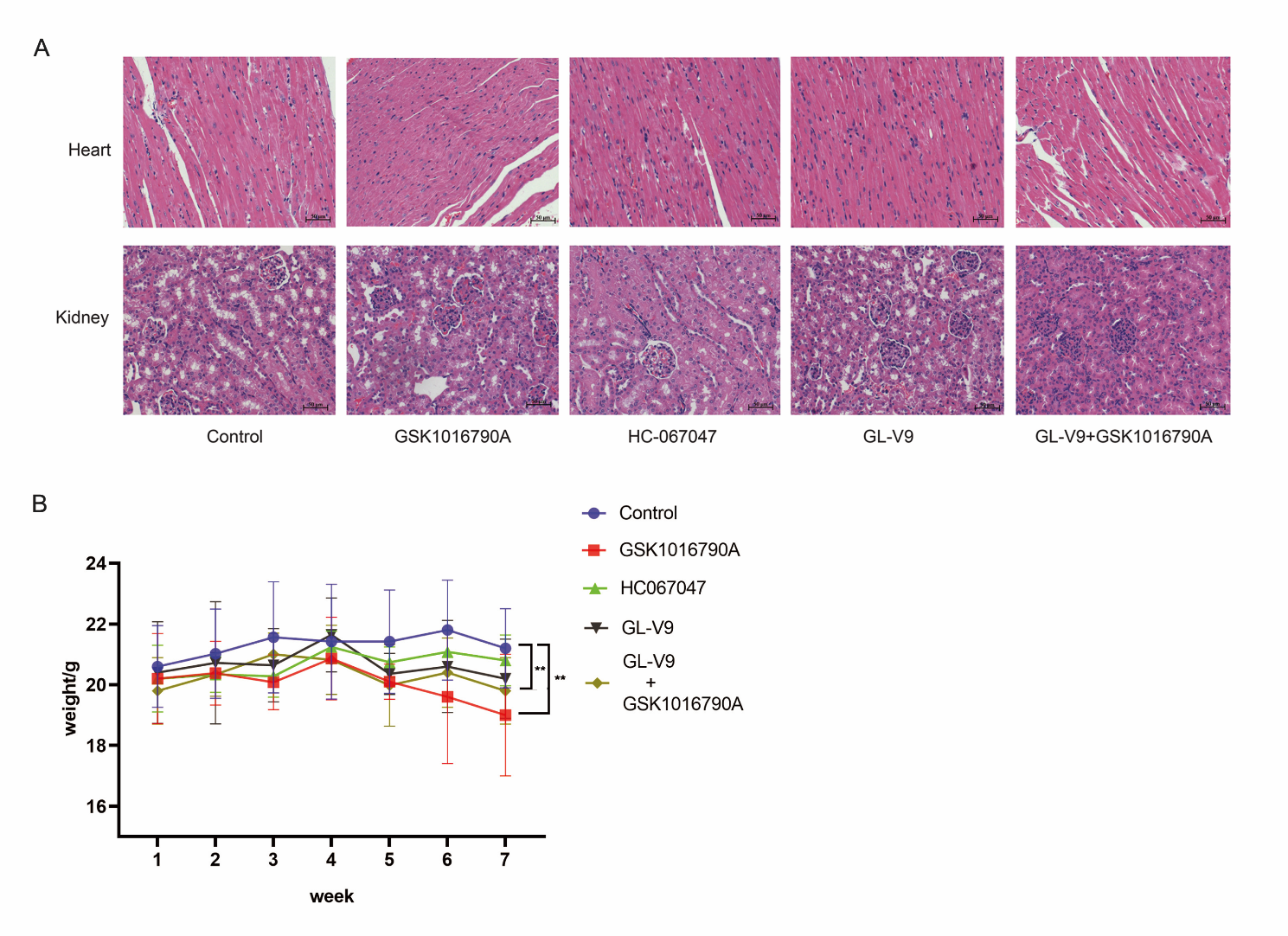


**Supplementary Figure S5** The metastatic HCC model was established via tail-vein injection of 7×10^6^ MHCC-97H cells per mouse (n=6). (**A**) HE staining for heart and kidneys tissue (20×, scale bars: 50 µm). (**B**) Mice body weight. *Bar*, SD. *^**^P*< 0.01 versus the untreated control.
