# Supplementary material for: Inhibition of TRPV4 remodels single cell polarity and suppresses the metastasis of hepatocellular carcinoma
Source: Cell Death Dis. 2023 Jun 28;14(6):379. doi: 10.1038/s41419-023-05903-z (PMC10300155; doi:10.1038/s41419-023-05903-z)
Supplement: Supplementary file 2 — Supplementary Tables [file 41419_2023_5903_MOESM2_ESM.pdf]

Supplementary Tables

Supplementary Table 1 The RNA expression of TRPV channel family in HCC cases from TCGA databases.

Supplementary Table 2 Clinical traits of hepatocellular carcinoma patients who express *TRPV2* or *TRPV4*.

Supplementary Table 3 The detailed patients' information of non-stage I HCC cases with *TRPV2* or *TRPV4* RNA low and high expression.

Supplementary Table 1 The RNA expression of TRPV channel family in HCC cases from TCGA databases.

TRPV1 RNA expression (N=365)

Sample	Description	FPKM
TCGA-ED-A66Y-01A	51 years, female, asian, stage:iiia, dead, 296 days	0.3
TCGA-DD-A4NK-01A	80 years, female, white, stage:iiia, dead, 1210 days	0.2
TCGA-DD-AADW-01A	48 years, male, asian, stage:i, alive, 587 days	0.2
TCGA-G3-A6UC-01A	65 years, male, white, stage:iiib, alive, 671 days	0.2
TCGA-DD-A4NJ-01A	54 years, female, white, stage:ii, alive, 928 days	0.2
TCGA-GJ-A9DB-01A	68 years, male, white, stage:i, dead, 67 days	0.2
TCGA-DD-A4ND-01A	56 years, female, white, stage:i, alive, 2746 days	0.2
TCGA-CC-A8HS-01A	18 years, male, asian, stage:iiic, dead, 300 days	0.2
TCGA-2Y-A9GZ-01A	82 years, female, white, stage:ii, dead, 848 days	0.2
TCGA-LG-A9QC-01A	48 years, male, white, stage:i, alive, 425 days	0.2
TCGA-2Y-A9GV-01A	54 years, female, white, stage:i, dead, 2532 days	0.2
TCGA-DD-A4NV-01A	61 years, male, white, stage:iiia, alive, 2398 days	0.2
TCGA-2Y-A9H6-01A	68 years, female, white, stage:i, alive, 357 days	0.2
TCGA-2Y-A9GU-01A	55 years, female, white, stage:i, alive, 1939 days	0.1
TCGA-DD-AACN-01A	32 years, male, asian, stage:i, alive, 1302 days	0.1
TCGA-ED-A66X-01A	35 years, male, asian, stage:iiia, alive, 406 days	0.1
TCGA-ZS-A9CF-01A	64 years, male, white, stage:ii, alive, 2412 days	0.1
TCGA-2Y-A9H5-01A	59 years, female, white, stage:i, dead, 555 days	0.1
TCGA-UB-A7MC-01A	59 years, male, white, stage:iiia, alive, 500 days	0.1
TCGA-XR-A8TC-01A	43 years, female, white, stage:i, alive, 1339 days	0.1
TCGA-DD-A4NL-01A	46 years, male, white, stage:i, alive, 1711 days	0.1
TCGA-DD-AACK-01A	70 years, male, asian, stage:i, alive, 9 days	0.1
TCGA-DD-AAVS-01A	56 years, male, asian, stage:i, alive, 1823 days	0.1
TCGA-DD-A11B-01A	73 years, male, white, stage:i, dead, 14 days	0.1
TCGA-DD-A4NO-01A	66 years, male, white, stage:i, alive, 2245 days	0.1
TCGA-5C-A9VG-01A	58 years, male, white, stage:ii, alive, 328 days	0.1
TCGA-DD-A1EF-01A	57 years, female, white, stage:i, dead, 394 days	0.1
TCGA-DD-A4NS-01A	62 years, female, white, stage:i, dead, 2456 days	0.1
TCGA-CC-A8HV-01A	51 years, female, asian, stage:ii, dead, 279 days	0.1
TCGA-DD-A73A-01A	71 years, male, white, stage:i, alive, 728 days	0.1
TCGA-DD-A4NI-01A	67 years, male, white, stage:ii, alive, 816 days	0.1
TCGA-2Y-A9H2-01A	64 years, female, white, stage:i, alive, 1731 days	0.1
TCGA-DD-AADK-01A	68 years, female, asian, stage:ii, alive, 1049 days	0.1
TCGA-DD-AACW-01A	43 years, male, asian, stage:i, alive, 1424 days	0.1
TCGA-DD-A4NQ-01A	60 years, male, white, stage:ii, dead, 373 days	0.1
TCGA-DD-AA3A-01A	81 years, female, white, stage:i, dead, 410 days	0.1
TCGA-2Y-A9GX-01A	68 years, male, white, stage:i, alive, 2442 days	0.1

TCGA-UB-AA0U-01A	60 years, male, white, stage:ii, alive, 327 days	0.1
TCGA-BC-A10T-01A	76 years, male, white, dead, 837 days	0.1
TCGA-2Y-A9HA-01A	70 years, male, white, stage:ii, dead, 36 days	0.1
TCGA-BD-A3ER-01A	62 years, male, white, stage:ii, alive, 1115 days	0.1
TCGA-2Y-A9GT-01A	51 years, male, white, stage:i, dead, 1624 days	0.1
TCGA-ZS-A9CE-01A	79 years, female, white, stage:ii, alive, 1241 days	0.1
TCGA-ED-A4XI-01A	58 years, male, asian, stage:ii, alive, 819 days	0.1
TCGA-DD-A4NH-01A	65 years, female, white, stage:iiib, alive, 917 days	0.1
TCGA-2Y-A9HB-01A	66 years, male, stage:i, alive, 260 days	0.1
TCGA-KR-A7K0-01A	65 years, male, white, stage:i, dead, 65 days	0.1
TCGA-DD-AADP-01A	45 years, male, asian, stage:i, alive, 458 days	0.1
TCGA-ZP-A9D4-01A	64 years, female, white, alive, 395 days	0.1
TCGA-DD-AACV-01A	53 years, male, asian, stage:i, alive, 1531 days	0.1
TCGA-DD-AAW0-01A	54 years, male, asian, stage:i, alive, 2015 days	0.1
TCGA-UB-A7MB-01A	24 years, male, white, stage:ii, alive, 601 days	0.1
TCGA-WQ-A9G7-01A	female, white, alive, 30 days	0.1
TCGA-DD-A73C-01A	65 years, female, white, stage:iiia, alive, 701 days	0.1
TCGA-HP-A5MZ-01A	62 years, male, stage:i, dead, 91 days	0.1
TCGA-CC-A7IE-01A	57 years, male, asian, stage:iiia, dead, 217 days	0.1
TCGA-DD-A113-01A	55 years, female, white, stage:ii, alive, 2425 days	0.1
TCGA-FV-A2QQ-01A	80 years, male, white, stage:i, alive, 729 days	0.1
TCGA-CC-A8HT-01A	74 years, male, asian, stage:iiia, dead, 140 days	0.1
TCGA-4R-AA8I-01A	66 years, male, white, stage:ii, dead, 262 days	0.1
TCGA-DD-AAVY-01A	56 years, male, asian, stage:iiia, alive, 1970 days	0.1
TCGA-XR-A8TF-01A	74 years, male, white, stage:i, dead, 693 days	0.1
TCGA-WX-AA47-01A	33 years, female, white, stage:iiia, dead, 556 days	0.1
TCGA-K7-A5RG-01A	66 years, male, black or african american, stage:i, alive, 519 days	0.1
TCGA-DD-AADY-01A	55 years, female, asian, stage:i, alive, 555 days	0.1
TCGA-DD-AAD8-01A	73 years, female, asian, stage:i, alive, 1219 days	0.1
TCGA-DD-A73D-01A	68 years, female, white, stage:ii, alive, 693 days	0.1
TCGA-ED-A8O6-01A	50 years, female, asian, stage:iiia, dead, 56 days	0.1
TCGA-ED-A7XP-01A	53 years, female, asian, stage:ii, alive, 400 days	0.1
TCGA-DD-A4NN-01A	56 years, female, white, stage:i, dead, 899 days	0.1
TCGA-EP-A2KC-01A	62 years, male, black or african american, stage:i, dead, 19 days	0.1
TCGA-ED-A7PY-01A	20 years, female, asian, stage:ii, alive, 390 days	0.1
TCGA-G3-A3CH-01A	53 years, male, asian, stage:iiia, alive, 780 days	0.1
TCGA-WJ-A86L-01A	68 years, female, white, stage:i, alive, 345 days	0.1
TCGA-DD-A4NB-01A	25 years, male, white, stage:i, alive, 989 days	0.1
TCGA-BC-A8YO-01A	66 years, female, white, stage:iiic, alive, 562 days	0.1
TCGA-DD-A4NR-01A	85 years, female, white, stage:i, dead, 9 days	0.1
TCGA-EP-A2KB-01A	46 years, female, white, stage:i, dead, 596 days	0.1
TCGA-2Y-A9H9-01A	70 years, male, white, stage:i, alive, 697 days	0.1
TCGA-ED-A97K-01A	54 years, male, asian, stage:iiia, alive, 6 days	0.1

TCGA-DD-AAEA-01A	65 years, male, asian, stage:i, alive, 575 days	0.1
TCGA-ZP-A9D0-01A	67 years, female, black or african american, alive, 1091 days	0.1
TCGA-DD-A4NE-01A	75 years, female, white, stage:iiia, dead, 660 days	0.1
TCGA-DD-A1EE-01A	73 years, male, white, stage:iiia, dead, 349 days	0.1
TCGA-DD-AAW3-01A	69 years, male, asian, stage:i, alive, 1633 days	0.1
TCGA-G3-A7M8-01A	31 years, male, asian, stage:i, alive, 430 days	0.1
TCGA-BD-A3EP-01A	76 years, female, black or african american, stage:i, alive, 409 days	0.1
TCGA-ED-A8O5-01A	59 years, female, asian, stage:iiia, alive, 406 days	0.1
TCGA-2Y-A9GS-01A	58 years, male, white, dead, 724 days	0.1
TCGA-RC-A7SH-01A	42 years, male, asian, stage:ii, alive, 468 days	0.1
TCGA-DD-AACQ-01A	50 years, male, asian, stage:ii, dead, 432 days	0.1
TCGA-FV-A496-01A	84 years, female, white, stage:i, alive, 10 days	0.1
TCGA-WX-AA46-01A	62 years, male, white, stage:ii, alive, 756 days	0.1
TCGA-ZP-A9CY-01A	66 years, female, white, alive, 782 days	0.1
TCGA-GJ-A3OU-01A	59 years, male, white, stage:i, alive, 879 days	0.1
TCGA-CC-A7IF-01A	59 years, male, asian, stage:iiia, dead, 649 days	0.1
TCGA-CC-A7IH-01A	58 years, male, asian, stage:iiia, alive, 365 days	0.1
TCGA-2Y-A9H7-01A	81 years, female, white, stage:i, alive, 1168 days	0.1
TCGA-ZP-A9D1-01A	56 years, female, white, alive, 21 days	0.1
TCGA-G3-A7M7-01A	65 years, male, white, stage:i, alive, 361 days	0.1
TCGA-DD-A4NA-01A	67 years, female, white, stage:iiic, alive, 1008 days	0.1
TCGA-G3-AAV1-01A	51 years, male, asian, stage:iiic, dead, 359 days	0.1
TCGA-UB-A7MA-01A	62 years, female, white, stage:ii, alive, 848 days	0.1
TCGA-RC-A7SK-01A	59 years, male, asian, stage:i, alive, 472 days	0.0
TCGA-UB-AA0V-01A	69 years, female, white, stage:i, alive, 314 days	0.0
TCGA-CC-A9FW-01A	68 years, male, asian, stage:iiia, alive, 248 days	0.0
TCGA-EP-A12J-01A	63 years, male, black or african american, stage:i, alive, 570 days	0.0
TCGA-FV-A4ZQ-01A	52 years, male, white, stage:i, alive, 12 days	0.0
TCGA-DD-A4NG-01A	77 years, male, white, stage:iiia, dead, 802 days	0.0
TCGA-MI-A75C-01A	64 years, male, white, stage:i, alive, 291 days	0.0
TCGA-DD-AAVW-01A	35 years, male, asian, stage:i, alive, 2317 days	0.0
TCGA-CC-A3MC-01A	54 years, male, asian, stage:iiia, alive, 363 days	0.0
TCGA-DD-AADC-01A	53 years, male, asian, stage:i, dead, 425 days	0.0
TCGA-K7-A6G5-01A	66 years, male, white, stage:i, alive, 512 days	0.0
TCGA-3K-AAZ8-01A	65 years, male, black or african american, stage:iiib, alive, 396 days	0.0
TCGA-2Y-A9GW-01A	64 years, male, white, stage:i, dead, 1271 days	0.0
TCGA-FV-A23B-01A	70 years, female, white, stage:ii, dead, 1852 days	0.0
TCGA-KR-A7K2-01A	64 years, male, white, stage:i, alive, 829 days	0.0
TCGA-G3-A7M6-01A	60 years, female, white, stage:i, alive, 632 days	0.0
TCGA-DD-A4NP-01A	32 years, male, white, stage:i, alive, 3308 days	0.0
TCGA-DD-AACT-01A	69 years, female, asian, stage:i, alive, 1562 days	0.0
TCGA-5C-A9VH-01A	70 years, male, white, stage:i, alive, 322 days	0.0
TCGA-DD-AAD0-01A	73 years, female, asian, stage:i, alive, 137 days	0.0

TCGA-DD-AADL-01A	58 years, male, asian, stage:i, alive, 636 days	0.0
TCGA-DD-AACX-01A	66 years, male, asian, stage:ii, alive, 170 days	0.0
TCGA-NI-A8LF-01A	74 years, male, white, stage:i, alive, 799 days	0.0
TCGA-DD-A73B-01A	72 years, female, white, stage:i, dead, 283 days	0.0
TCGA-DD-AAE0-01A	45 years, female, asian, stage:iiia, alive, 555 days	0.0
TCGA-BC-A10U-01A	69 years, male, white, dead, 837 days	0.0
TCGA-BC-A3KF-01A	66 years, female, white, stage:i, alive, 8 days	0.0
TCGA-DD-AAVZ-01A	38 years, male, asian, stage:i, alive, 1900 days	0.0
TCGA-G3-A5SM-01A	58 years, male, white, stage:ii, alive, 520 days	0.0
TCGA-G3-AAV6-01A	53 years, female, white, stage:iiia, dead, 65 days	0.0
TCGA-K7-A5RF-01A	64 years, male, white, stage:i, alive, 631 days	0.0
TCGA-DD-AACP-01A	65 years, male, asian, stage:i, alive, 415 days	0.0
TCGA-CC-A9FS-01A	55 years, male, asian, stage:ii, alive, 211 days	0.0
TCGA-G3-AAV7-01A	38 years, male, asian, stage:ii, alive, 361 days	0.0
TCGA-MR-A8JO-01A	34 years, male, white, stage:i, alive, 330 days	0.0
TCGA-CC-5258-01A	48 years, male, asian, stage:ii, dead, 129 days	0.0
TCGA-FV-A3I1-01A	female, white, stage:ii, dead, 247 days	0.0
TCGA-CC-A5UE-01A	48 years, male, asian, stage:iiib, dead, 272 days	0.0
TCGA-LG-A9QD-01A	68 years, male, white, stage:iiia, alive, 366 days	0.0
TCGA-XR-A8TE-01A	16 years, male, white, stage:iiia, alive, 925 days	0.0
TCGA-HP-A5N0-01A	88 years, female, dead, 1147 days	0.0
TCGA-DD-A1EB-01A	72 years, female, stage:i, alive, 2017 days	0.0
TCGA-FV-A2QR-01A	male, white, stage:i, dead, 581 days	0.0
TCGA-BC-A10Q-01A	72 years, female, white, dead, 1135 days	0.0
TCGA-G3-A25U-01A	63 years, female, asian, stage:i, alive, 1636 days	0.0
TCGA-FV-A3R3-01A	38 years, female, white, stage:i, dead, 366 days	0.0
TCGA-G3-A7M9-01A	70 years, male, white, stage:iiib, dead, 56 days	0.0
TCGA-ED-A627-01A	74 years, male, white, stage:i, alive, 423 days	0.0
TCGA-DD-AADA-01A	66 years, female, asian, stage:i, alive, 1233 days	0.0
TCGA-FV-A3R2-01A	75 years, male, white, stage:i, dead, 194 days	0.0
TCGA-GJ-A6C0-01A	75 years, female, white, stage:ii, dead, 31 days	0.0
TCGA-LG-A6GG-01A	79 years, female, white, stage:ii, alive, 387 days	0.0
TCGA-MR-A520-01A	58 years, male, white, stage:i, alive, 229 days	0.0
TCGA-ZP-A9CZ-01A	72 years, male, asian, alive, 706 days	0.0
TCGA-DD-AAE1-01A	52 years, male, asian, stage:i, alive, 552 days	0.0
TCGA-DD-AAW1-01A	55 years, male, asian, stage:iiia, alive, 1989 days	0.0
TCGA-XR-A8TG-01A	59 years, male, white, stage:i, alive, 898 days	0.0
TCGA-ZS-A9CD-01A	73 years, male, white, stage:ii, dead, 1386 days	0.0
TCGA-QA-A7B7-01A	48 years, male, black or african american, stage:ii, alive, 94 days	0.0
TCGA-YA-A8S7-01A	69 years, male, white, stage:iiia, dead, 412 days	0.0
TCGA-DD-AAE2-01A	51 years, male, asian, stage:i, alive, 638 days	0.0
TCGA-CC-A7IL-01A	61 years, male, asian, stage:iiia, dead, 278 days	0.0
TCGA-DD-AAEG-01A	59 years, female, asian, stage:i, alive, 719 days	0.0

TCGA-BC-A10Z-01A	62 years, female, white, stage:i, dead, 34 days	0.0
TCGA-DD-AADG-01A	70 years, male, asian, stage:iiia, alive, 1145 days	0.0
TCGA-DD-A3A2-01A	76 years, female, white, stage:i, dead, 2131 days	0.0
TCGA-BC-A216-01A	62 years, female, white, stage:iiia, alive, 1351 days	0.0
TCGA-EP-A3RK-01A	73 years, male, white, stage:iiia, alive, 363 days	0.0
TCGA-DD-AAD2-01A	66 years, male, asian, stage:i, alive, 658 days	0.0
TCGA-ZP-A9D2-01A	51 years, male, white, dead, 765 days	0.0
TCGA-G3-AAV2-01A	50 years, male, white, stage:i, alive, 372 days	0.0
TCGA-BC-A10W-01A	50 years, male, asian, dead, 91 days	0.0
TCGA-K7-AAU7-01A	61 years, male, white, stage:ii, alive, 359 days	0.0
TCGA-ZP-A9CV-01A	59 years, male, white, dead, 1088 days	0.0
TCGA-DD-AAE6-01A	59 years, female, asian, stage:i, alive, 141 days	0.0
TCGA-DD-AACS-01A	39 years, male, asian, stage:i, alive, 1804 days	0.0
TCGA-BC-A3KG-01A	68 years, female, white, stage:ii, alive, 680 days	0.0
TCGA-EP-A26S-01A	70 years, male, white, stage:i, alive, 608 days	0.0
TCGA-BC-A112-01A	80 years, male, white, dead, 153 days	0.0
TCGA-BC-A10Y-01A	76 years, male, white, dead, 711 days	0.0
TCGA-BC-A10S-01A	81 years, male, white, dead, 1423 days	0.0
TCGA-DD-AADB-01A	51 years, male, asian, stage:i, alive, 1242 days	0.0
TCGA-T1-A6J8-01A	68 years, male, white, alive, 23 days	0.0
TCGA-2Y-A9H4-01A	68 years, male, black or african american, stage:i, alive, 1452 days	0.0
TCGA-DD-AAEK-01A	51 years, male, asian, stage:ii, alive, 1067 days	0.0
TCGA-KR-A7K7-01A	61 years, female, white, stage:ii, alive, 951 days	0.0
TCGA-CC-A3MB-01A	36 years, male, asian, stage:iiia, dead, 315 days	0.0
TCGA-DD-AAVX-01A	38 years, male, asian, stage:ii, alive, 1570 days	0.0
TCGA-DD-A11D-01A	57 years, female, white, stage:i, dead, 1560 days	0.0
TCGA-DD-A1EA-01A	68 years, male, asian, stage:ii, alive, 2415 days	0.0
TCGA-DD-A11A-01A	67 years, male, black or african american, stage:i, alive, 79 days	0.0
TCGA-DD-AAW2-01A	69 years, male, asian, stage:i, alive, 1855 days	0.0
TCGA-DD-A39W-01A	29 years, female, white, stage:iii, dead, 827 days	0.0
TCGA-CC-A7IJ-01A	56 years, male, asian, stage:ii, alive, 382 days	0.0
TCGA-RG-A7D4-01A	69 years, male, black or african american, stage:ii, alive, 1098 days	0.0
TCGA-2Y-A9H8-01A	85 years, female, white, dead, 633 days	0.0
TCGA-MI-A75G-01A	63 years, male, white, stage:ii, alive, 698 days	0.0
TCGA-FV-A3I0-01A	76 years, female, white, stage:ii, alive, 848 days	0.0
TCGA-DD-AADD-01A	51 years, male, asian, stage:i, alive, 1231 days	0.0
TCGA-NI-A4U2-01A	71 years, male, white, stage:iiia, dead, 1791 days	0.0
TCGA-MI-A75H-01A	77 years, male, white, alive, 747 days	0.0
TCGA-DD-A1EH-01A	23 years, male, white, stage:iii, alive, 1495 days	0.0
TCGA-XR-A8TD-01A	49 years, female, white, stage:iiib, alive, 1030 days	0.0
TCGA-DD-AAD5-01A	54 years, male, asian, stage:i, alive, 1345 days	0.0
TCGA-RC-A7SB-01A	53 years, male, asian, stage:ii, alive, 588 days	0.0
TCGA-DD-AAE9-01A	69 years, male, asian, stage:i, alive, 722 days	0.0

TCGA-5R-AAAM-01A	65 years, female, white, stage:ii, dead, 46 days	0.0
TCGA-ED-A459-01A	47 years, male, asian, stage:ii, alive, 910 days	0.0
TCGA-ED-A82E-01A	60 years, female, asian, stage:iiia, alive, 408 days	0.0
TCGA-DD-AAEE-01A	55 years, male, asian, stage:i, alive, 810 days	0.0
TCGA-PD-A5DF-01A	58 years, female, white, stage:iiib, dead, 639 days	0.0
TCGA-G3-A3CI-01A	71 years, male, white, stage:i, alive, 180 days	0.0
TCGA-G3-AAV3-01A	58 years, female, white, stage:ii, alive, 412 days	0.0
TCGA-DD-AADV-01A	50 years, male, asian, stage:i, alive, 574 days	0.0
TCGA-DD-A1ED-01A	68 years, male, white, stage:i, alive, 2301 days	0.0
TCGA-G3-A7M5-01A	76 years, male, asian, stage:i, alive, 447 days	0.0
TCGA-G3-A3CK-01A	61 years, male, asian, stage:i, alive, 585 days	0.0
TCGA-DD-A3A8-01A	75 years, male, white, stage:ii, dead, 11 days	0.0
TCGA-DD-A118-01A	77 years, female, white, stage:ii, alive, 3437 days	0.0
TCGA-RC-A6M4-01A	74 years, female, white, stage:iiia, alive, 22 days	0.0
TCGA-ED-A5KG-01A	60 years, female, asian, stage:ii, alive, 854 days	0.0
TCGA-DD-A1EK-01A	64 years, female, white, stage:ivb, dead, 558 days	0.0
TCGA-CC-A3MA-01A	61 years, male, asian, stage:iiia, dead, 303 days	0.0
TCGA-KR-A7K8-01A	57 years, male, stage:i, alive, 906 days	0.0
TCGA-DD-AAD3-01A	43 years, male, asian, stage:i, alive, 1295 days	0.0
TCGA-DD-AAEH-01A	73 years, male, asian, stage:i, alive, 784 days	0.0
TCGA-G3-AAV4-01A	83 years, female, white, stage:i, dead, 27 days	0.0
TCGA-DD-AAED-01A	51 years, male, asian, stage:i, alive, 763 days	0.0
TCGA-RC-A7SF-01A	66 years, male, asian, stage:i, alive, 579 days	0.0
TCGA-BC-4072-01B	74 years, female, white, stage:iiia, dead, 1490 days	0.0
TCGA-G3-A25V-01A	68 years, male, white, stage:i, alive, 860 days	0.0
TCGA-DD-A73E-01A	66 years, male, white, stage:i, alive, 44 days	0.0
TCGA-DD-AAEB-01A	60 years, male, asian, stage:i, alive, 478 days	0.0
TCGA-5R-AA1C-01A	57 years, male, white, stage:ii, alive, 520 days	0.0
TCGA-UB-A7ME-01A	51 years, male, asian, stage:i, alive, 486 days	0.0
TCGA-CC-5260-01A	61 years, female, asian, stage:iiic, dead, 87 days	0.0
TCGA-DD-A1EL-01A	23 years, male, black or african american, stage:ii, dead, 415 days	0.0
TCGA-DD-AACA-01A	65 years, male, asian, stage:i, alive, 2301 days	0.0
TCGA-G3-A5SJ-01A	59 years, male, white, stage:i, alive, 698 days	0.0
TCGA-RC-A7S9-01A	47 years, female, asian, stage:i, alive, 640 days	0.0
TCGA-CC-5261-01A	44 years, male, asian, stage:ii, dead, 97 days	0.0
TCGA-DD-AAC8-01A	72 years, male, asian, stage:i, dead, 16 days	0.0
TCGA-DD-AADQ-01A	59 years, male, asian, stage:ii, alive, 436 days	0.0
TCGA-EP-A3JL-01A	76 years, male, white, stage:i, alive, 303 days	0.0
TCGA-DD-AACE-01A	62 years, male, asian, stage:i, alive, 2184 days	0.0
TCGA-RC-A6M5-01A	20 years, female, white, stage:iva, alive, 15 days	0.0
TCGA-DD-AAD6-01A	66 years, male, asian, stage:iiia, alive, 672 days	0.0
TCGA-CC-A7IG-01A	47 years, male, asian, stage:ii, dead, 299 days	0.0
TCGA-DD-AACY-01A	61 years, male, asian, stage:i, alive, 1450 days	0.0

TCGA-DD-A4NF-01A	72 years, male, white, stage:i, alive, 942 days	0.0
TCGA-MI-A75E-01A	61 years, male, white, stage:iiic, alive, 507 days	0.0
TCGA-DD-A1EC-01A	20 years, female, white, stage:i, alive, 602 days	0.0
TCGA-DD-AAD1-01A	51 years, female, asian, stage:i, alive, 564 days	0.0
TCGA-CC-A7IK-01A	59 years, male, asian, stage:iiia, dead, 262 days	0.0
TCGA-CC-A1HT-01A	50 years, male, asian, stage:iiia, dead, 101 days	0.0
TCGA-DD-AAVP-01A	48 years, male, asian, stage:i, alive, 2752 days	0.0
TCGA-G3-A3CG-01A	80 years, male, white, stage:i, alive, 673 days	0.0
TCGA-DD-AAC9-01A	51 years, male, asian, stage:i, alive, 347 days	0.0
TCGA-RC-A6M6-01A	75 years, male, white, stage:ii, alive, 9 days	0.0
TCGA-DD-A1EI-01A	46 years, male, asian, stage:i, alive, 183 days	0.0
TCGA-G3-A25Y-01A	52 years, female, asian, stage:i, dead, 452 days	0.0
TCGA-ED-A7PX-01A	48 years, female, asian, stage:ii, alive, 6 days	0.0
TCGA-DD-A114-01A	42 years, male, black or african american, stage:ii, dead, 1149 days	0.0
TCGA-DD-AADU-01A	60 years, male, asian, stage:ii, alive, 554 days	0.0
TCGA-CC-5264-01A	71 years, male, asian, stage:iiia, dead, 102 days	0.0
TCGA-BC-A110-01A	51 years, female, black or african american, dead, 2116 days	0.0
TCGA-G3-AAV5-01A	67 years, male, white, stage:ii, alive, 354 days	0.0
TCGA-G3-A5SL-01A	70 years, male, white, stage:ii, alive, 621 days	0.0
TCGA-DD-AACU-01A	59 years, male, asian, stage:i, alive, 1567 days	0.0
TCGA-2Y-A9GY-01A	64 years, female, white, stage:ii, dead, 757 days	0.0
TCGA-DD-AAE7-01A	72 years, male, asian, stage:i, alive, 644 days	0.0
TCGA-DD-A1EG-01A	77 years, male, white, stage:i, dead, 1372 days	0.0
TCGA-DD-AACZ-01A	63 years, female, asian, stage:i, dead, 171 days	0.0
TCGA-DD-A1EJ-01A	71 years, female, white, stage:iiic, dead, 1005 days	0.0
TCGA-WQ-AB4B-01A	62 years, male, white, stage:ii, alive, 395 days	0.0
TCGA-UB-A7MD-01A	67 years, male, black or african american, stage:i, dead, 52 days	0.0
TCGA-CC-A5UC-01A	63 years, male, asian, stage:iiia, dead, 347 days	0.0
TCGA-DD-A73F-01A	77 years, female, white, stage:i, alive, 1085 days	0.0
TCGA-EP-A2KA-01A	52 years, female, white, stage:iiia, dead, 627 days	0.0
TCGA-ED-A7XO-01A	29 years, male, asian, stage:iiia, alive, 427 days	0.0
TCGA-O8-A75V-01A	54 years, male, stage:i, alive, 538 days	0.0
TCGA-CC-5263-01A	35 years, male, asian, stage:iiia, dead, 129 days	0.0
TCGA-BD-A2L6-01A	69 years, male, white, alive, 1363 days	0.0
TCGA-BC-A10R-01A	66 years, female, white, dead, 308 days	0.0
TCGA-CC-A5UD-01A	45 years, male, asian, stage:iiia, dead, 304 days	0.0
TCGA-BC-4073-01B	73 years, male, white, stage:iiia, alive, 849 days	0.0
TCGA-DD-AAVV-01A	56 years, male, asian, stage:ii, alive, 2455 days	0.0
TCGA-CC-A8HU-01A	39 years, female, asian, stage:iiia, dead, 344 days	0.0
TCGA-UB-A7MF-01A	57 years, male, white, stage:iiia, dead, 214 days	0.0
TCGA-DD-AAVU-01A	46 years, male, asian, stage:ii, alive, 2202 days	0.0
TCGA-G3-A25Z-01A	58 years, male, asian, stage:i, alive, 655 days	0.0
TCGA-DD-A11C-01A	69 years, male, white, stage:i, alive, 662 days	0.0

TCGA-DD-AADM-01A	58 years, male, asian, stage:ii, dead, 12 days	0.0
TCGA-BC-A10X-01A	52 years, female, white, stage:iiia, dead, 770 days	0.0
TCGA-DD-AADO-01A	55 years, male, asian, stage:i, alive, 453 days	0.0
TCGA-5R-AA1D-01A	17 years, female, white, stage:iiia, alive, 449 days	0.0
TCGA-DD-AACL-01A	66 years, female, asian, stage:i, dead, 107 days	0.0
TCGA-ZS-A9CG-01A	55 years, male, white, stage:ii, alive, 341 days	0.0
TCGA-BC-A69I-01A	69 years, male, white, stage:i, alive, 387 days	0.0
TCGA-DD-A3A4-01A	37 years, male, white, stage:iiia, dead, 612 days	0.0
TCGA-DD-A73G-01A	73 years, female, white, stage:i, alive, 3478 days	0.0
TCGA-DD-AAEI-01A	72 years, male, asian, stage:i, alive, 1531 days	0.0
TCGA-BW-A5NO-01A	50 years, male, black or african american, stage:iiia, alive, 20 days	0.0
TCGA-DD-AAVQ-01A	38 years, male, asian, stage:i, alive, 2728 days	0.0
TCGA-WX-AA44-01A	64 years, female, white, stage:i, alive, 615 days	0.0
TCGA-CC-5259-01A	60 years, female, asian, stage:iiic, alive, 250 days	0.0
TCGA-DD-A39Y-01A	67 years, male, asian, stage:i, dead, 171 days	0.0
TCGA-DD-AACH-01A	69 years, male, asian, stage:ii, dead, 195 days	0.0
TCGA-DD-AADJ-01A	70 years, female, asian, stage:i, alive, 1066 days	0.0
TCGA-DD-AAE3-01A	50 years, male, asian, stage:i, alive, 566 days	0.0
TCGA-DD-AACG-01A	52 years, male, asian, stage:ii, dead, 469 days	0.0
TCGA-DD-AACC-01A	61 years, male, asian, stage:i, dead, 1685 days	0.0
TCGA-DD-AADS-01A	63 years, male, asian, stage:i, alive, 474 days	0.0
TCGA-5C-AAPD-01A	61 years, male, asian, stage:ii, alive, 20 days	0.0
TCGA-DD-AADR-01A	58 years, male, asian, stage:i, alive, 2028 days	0.0
TCGA-DD-A3A6-01A	72 years, female, white, stage:ii, dead, 3258 days	0.0
TCGA-BC-A5W4-01A	69 years, male, white, stage:iiia, dead, 547 days	0.0
TCGA-DD-A115-01A	53 years, male, white, stage:iiia, dead, 2542 days	0.0
TCGA-DD-A39Z-01A	43 years, female, stage:ii, dead, 601 days	0.0
TCGA-G3-A5SI-01A	44 years, male, asian, stage:ii, dead, 768 days	0.0
TCGA-G3-A5SK-01A	58 years, male, white, stage:i, alive, 744 days	0.0
TCGA-DD-A3A5-01A	66 years, female, white, stage:iii, dead, 3125 days	0.0
TCGA-DD-A3A9-01A	64 years, female, white, stage:ivb, dead, 931 days	0.0
TCGA-G3-A25S-01A	64 years, male, white, stage:i, dead, 416 days	0.0
TCGA-DD-A3A1-01A	65 years, male, stage:iiia, dead, 233 days	0.0
TCGA-DD-AACF-01A	68 years, male, asian, stage:i, dead, 365 days	0.0
TCGA-2Y-A9H0-01A	49 years, male, white, stage:iiia, alive, 3675 days	0.0
TCGA-MI-A75I-01A	61 years, male, black or african american, alive, 630 days	0.0
TCGA-CC-A3M9-01A	45 years, male, asian, stage:iiia, dead, 300 days	0.0
TCGA-BC-A217-01A	75 years, female, white, stage:ii, dead, 1397 days	0.0
TCGA-ED-A7PZ-01A	61 years, male, asian, stage:ii, alive, 6 days	0.0
TCGA-DD-AACJ-01A	75 years, male, asian, stage:ii, alive, 2102 days	0.0
TCGA-DD-AADN-01A	59 years, male, asian, stage:i, alive, 898 days	0.0
TCGA-DD-AAE4-01A	49 years, female, asian, stage:i, alive, 608 days	0.0
TCGA-DD-AACB-01A	74 years, female, asian, stage:i, alive, 2324 days	0.0

TCGA-DD-A39V-01A	77 years, male, white, stage:ii, dead, 643 days	0.0
TCGA-ES-A2HS-01A	80 years, male, white, stage:i, dead, 688 days	0.0
TCGA-DD-A39X-01A	78 years, female, white, stage:i, dead, 1694 days	0.0
TCGA-DD-AADI-01A	43 years, female, asian, stage:i, alive, 1085 days	0.0
TCGA-2Y-A9H1-01A	58 years, male, white, stage:i, dead, 1229 days	0.0
TCGA-2Y-A9H3-01A	45 years, male, white, stage:ii, alive, 1516 days	0.0
TCGA-G3-A25T-01A	45 years, female, white, stage:iiia, alive, 1553 days	0.0
TCGA-DD-AACI-01A	69 years, male, asian, stage:ii, alive, 1618 days	0.0
TCGA-DD-A116-01A	68 years, male, asian, stage:iiia, dead, 1622 days	0.0
TCGA-DD-A3A3-01A	45 years, male, asian, stage:i, dead, 535 days	0.0
TCGA-G3-A25X-01A	73 years, male, asian, stage:ii, alive, 1779 days	0.0
TCGA-DD-A119-01A	40 years, male, asian, stage:iv, dead, 223 days	0.0
TCGA-G3-AAUZ-01A	48 years, male, stage:i, alive, 480 days	0.0
TCGA-G3-A3CJ-01A	52 years, male, american indian or alaska native, stage:ii, alive, 594 days	0.0
TCGA-BC-A69H-01A	64 years, male, white, stage:ii, alive, 444 days	0.0
TCGA-CC-5262-01A	67 years, male, asian, stage:iiic, dead, 103 days	0.0
TCGA-DD-AADF-01A	64 years, female, asian, stage:i, dead, 115 days	0.0
TCGA-CC-A123-01A	24 years, female, asian, stage:iiia, alive, 219 days	0.0
TCGA-G3-AAV0-01A	58 years, male, asian, stage:i, alive, 476 days	0.0
TCGA-DD-AACD-01A	48 years, male, asian, stage:i, dead, 381 days	0.0
TCGA-DD-AACO-01A	40 years, male, asian, stage:i, alive, 1876 days	0.0
TCGA-DD-A3A7-01A	67 years, male, stage:iiib, dead, 419 days	0.0
TCGA-ES-A2HT-01A	54 years, male, black or african american, stage:i, dead, 438 days	0.0
TCGA-DD-AAVR-01A	44 years, male, asian, stage:i, alive, 2513 days	0.0
TCGA-FV-A4ZP-01A	78 years, male, white, stage:iiia, dead, 2486 days	0.0
TCGA-CC-A7II-01A	55 years, male, asian, stage:iiia, alive, 399 days	0.0
TCGA-FV-A495-01A	51 years, female, white, stage:ii, alive, 1 days	0.0

TRPV2 RNA expression (N=365)

Sample	Description	FPKM
TCGA-DD-AAD5-01A	54 years, male, asian, stage:i, alive, 1345 days	51.4
TCGA-RC-A6M6-01A	75 years, male, white, stage:ii, alive, 9 days	37.8
TCGA-DD-AACZ-01A	63 years, female, asian, stage:i, dead, 171 days	32.8
TCGA-2Y-A9GY-01A	64 years, female, white, stage:ii, dead, 757 days	29.9
TCGA-FV-A4ZP-01A	78 years, male, white, stage:iiia, dead, 2486 days	21.9
TCGA-DD-A3A6-01A	72 years, female, white, stage:ii, dead, 3258 days	21.8
TCGA-DD-AACH-01A	69 years, male, asian, stage:ii, dead, 195 days	21.2
TCGA-CC-A7IL-01A	61 years, male, asian, stage:iiia, dead, 278 days	20.8
TCGA-GJ-A3OU-01A	59 years, male, white, stage:i, alive, 879 days	20.6
TCGA-BC-4073-01B	73 years, male, white, stage:iiia, alive, 849 days	19.0
TCGA-DD-A113-01A	55 years, female, white, stage:ii, alive, 2425 days	16.1
TCGA-CC-A7IE-01A	57 years, male, asian, stage:iiia, dead, 217 days	15.7

TCGA-CC-A3M9-01A	45 years, male, asian, stage:iiia, dead, 300 days	15.4
TCGA-CC-A5UE-01A	48 years, male, asian, stage:iiib, dead, 272 days	11.4
TCGA-ED-A5KG-01A	60 years, female, asian, stage:ii, alive, 854 days	11.2
TCGA-CC-A8HT-01A	74 years, male, asian, stage:iiia, dead, 140 days	10.7
TCGA-G3-A25S-01A	64 years, male, white, stage:i, dead, 416 days	10.1
TCGA-DD-A4NR-01A	85 years, female, white, stage:i, dead, 9 days	9.9
TCGA-5C-AAPD-01A	61 years, male, asian, stage:ii, alive, 20 days	8.2
TCGA-DD-A114-01A	42 years, male, black or african american, stage:ii, dead, 1149 days	7.9
TCGA-CC-A1HT-01A	50 years, male, asian, stage:iiia, dead, 101 days	7.7
TCGA-EP-A3RK-01A	73 years, male, white, stage:iiia, alive, 363 days	7.5
TCGA-K7-A5RG-01A	66 years, male, black or african american, stage:i, alive, 519 days	6.9
TCGA-CC-A3MA-01A	61 years, male, asian, stage:iiia, dead, 303 days	6.7
TCGA-DD-A1EC-01A	20 years, female, white, stage:i, alive, 602 days	6.7
TCGA-DD-AACI-01A	69 years, male, asian, stage:ii, alive, 1618 days	6.6
TCGA-XR-A8TD-01A	49 years, female, white, stage:iiib, alive, 1030 days	6.0
TCGA-G3-A7M9-01A	70 years, male, white, stage:iiib, dead, 56 days	6.0
TCGA-DD-AACL-01A	66 years, female, asian, stage:i, dead, 107 days	6.0
TCGA-DD-A4NS-01A	62 years, female, white, stage:i, dead, 2456 days	5.9
TCGA-CC-5260-01A	61 years, female, asian, stage:iiic, dead, 87 days	5.7
TCGA-YA-A8S7-01A	69 years, male, white, stage:iiia, dead, 412 days	5.5
TCGA-CC-A7IJ-01A	56 years, male, asian, stage:ii, alive, 382 days	5.4
TCGA-DD-AACC-01A	61 years, male, asian, stage:i, dead, 1685 days	5.3
TCGA-KR-A7K8-01A	57 years, male, stage:i, alive, 906 days	5.3
TCGA-MR-A8JO-01A	34 years, male, white, stage:i, alive, 330 days	5.1
TCGA-DD-AAVQ-01A	38 years, male, asian, stage:i, alive, 2728 days	5.1
TCGA-G3-A25X-01A	73 years, male, asian, stage:ii, alive, 1779 days	5.1
TCGA-2Y-A9H3-01A	45 years, male, white, stage:ii, alive, 1516 days	5.0
TCGA-2Y-A9GW-01A	64 years, male, white, stage:i, dead, 1271 days	5.0
TCGA-ED-A627-01A	74 years, male, white, stage:i, alive, 423 days	5.0
TCGA-CC-5261-01A	44 years, male, asian, stage:ii, dead, 97 days	4.8
TCGA-2Y-A9GX-01A	68 years, male, white, stage:i, alive, 2442 days	4.6
TCGA-ED-A66X-01A	35 years, male, asian, stage:iiia, alive, 406 days	4.6
TCGA-FV-A495-01A	51 years, female, white, stage:ii, alive, 1 days	4.5
TCGA-DD-A1EG-01A	77 years, male, white, stage:i, dead, 1372 days	4.4
TCGA-DD-AAD1-01A	51 years, female, asian, stage:i, alive, 564 days	4.3
TCGA-GJ-A6C0-01A	75 years, female, white, stage:ii, dead, 31 days	4.3
TCGA-2Y-A9HA-01A	70 years, male, white, stage:ii, dead, 36 days	4.3
TCGA-DD-A3A3-01A	45 years, male, asian, stage:i, dead, 535 days	4.2
TCGA-DD-A4NB-01A	25 years, male, white, stage:i, alive, 989 days	4.1
TCGA-ED-A7PX-01A	48 years, female, asian, stage:ii, alive, 6 days	4.1
TCGA-DD-A11A-01A	67 years, male, black or african american, stage:i, alive, 79 days	4.1
TCGA-2Y-A9H0-01A	49 years, male, white, stage:iiia, alive, 3675 days	4.1
TCGA-DD-A4NA-01A	67 years, female, white, stage:iiic, alive, 1008 days	4.1

TCGA-DD-A73F-01A	77 years, female, white, stage:i, alive, 1085 days	4.0
TCGA-5R-AA1D-01A	17 years, female, white, stage:iiia, alive, 449 days	4.0
TCGA-DD-AAVV-01A	56 years, male, asian, stage:ii, alive, 2455 days	3.9
TCGA-FV-A4ZQ-01A	52 years, male, white, stage:i, alive, 12 days	3.9
TCGA-PD-A5DF-01A	58 years, female, white, stage:iiib, dead, 639 days	3.8
TCGA-G3-AAV3-01A	58 years, female, white, stage:ii, alive, 412 days	3.8
TCGA-DD-AAW0-01A	54 years, male, asian, stage:i, alive, 2015 days	3.8
TCGA-DD-A3A9-01A	64 years, female, white, stage:ivb, dead, 931 days	3.7
TCGA-DD-AADR-01A	58 years, male, asian, stage:i, alive, 2028 days	3.7
TCGA-HP-A5MZ-01A	62 years, male, stage:i, dead, 91 days	3.7
TCGA-DD-AAE4-01A	49 years, female, asian, stage:i, alive, 608 days	3.6
TCGA-CC-5262-01A	67 years, male, asian, stage:iiic, dead, 103 days	3.6
TCGA-G3-A7M5-01A	76 years, male, asian, stage:i, alive, 447 days	3.6
TCGA-UB-A7MF-01A	57 years, male, white, stage:iiia, dead, 214 days	3.6
TCGA-DD-AADB-01A	51 years, male, asian, stage:i, alive, 1242 days	3.5
TCGA-ED-A8O6-01A	50 years, female, asian, stage:iiia, dead, 56 days	3.5
TCGA-FV-A3R3-01A	38 years, female, white, stage:i, dead, 366 days	3.5
TCGA-ED-A97K-01A	54 years, male, asian, stage:iiia, alive, 6 days	3.5
TCGA-DD-A4ND-01A	56 years, female, white, stage:i, alive, 2746 days	3.5
TCGA-DD-AAVW-01A	35 years, male, asian, stage:i, alive, 2317 days	3.5
TCGA-DD-AADI-01A	43 years, female, asian, stage:i, alive, 1085 days	3.5
TCGA-BD-A3EP-01A	76 years, female, black or african american, stage:i, alive, 409 days	3.5
TCGA-BC-A10S-01A	81 years, male, white, dead, 1423 days	3.4
TCGA-BC-4072-01B	74 years, female, white, stage:iiia, dead, 1490 days	3.4
TCGA-DD-AACB-01A	74 years, female, asian, stage:i, alive, 2324 days	3.4
TCGA-DD-A39V-01A	77 years, male, white, stage:ii, dead, 643 days	3.4
TCGA-DD-A39X-01A	78 years, female, white, stage:i, dead, 1694 days	3.2
TCGA-EP-A3JL-01A	76 years, male, white, stage:i, alive, 303 days	3.2
TCGA-DD-AAVR-01A	44 years, male, asian, stage:i, alive, 2513 days	3.2
TCGA-CC-A5UC-01A	63 years, male, asian, stage:iiia, dead, 347 days	3.2
TCGA-BC-A69H-01A	64 years, male, white, stage:ii, alive, 444 days	3.1
TCGA-UB-A7ME-01A	51 years, male, asian, stage:i, alive, 486 days	3.1
TCGA-UB-A7MD-01A	67 years, male, black or african american, stage:i, dead, 52 days	3.1
TCGA-DD-AAEK-01A	51 years, male, asian, stage:ii, alive, 1067 days	3.1
TCGA-DD-AAD2-01A	66 years, male, asian, stage:i, alive, 658 days	3.0
TCGA-FV-A3I1-01A	female, white, stage:ii, dead, 247 days	3.0
TCGA-G3-A25Y-01A	52 years, female, asian, stage:i, dead, 452 days	3.0
TCGA-G3-A25T-01A	45 years, female, white, stage:iiia, alive, 1553 days	3.0
TCGA-5R-AAAM-01A	65 years, female, white, stage:ii, dead, 46 days	3.0
TCGA-ZP-A9CZ-01A	72 years, male, asian, alive, 706 days	3.0
TCGA-K7-AAU7-01A	61 years, male, white, stage:ii, alive, 359 days	2.9
TCGA-BC-A112-01A	80 years, male, white, dead, 153 days	2.9
TCGA-ED-A8O5-01A	59 years, female, asian, stage:iiia, alive, 406 days	2.9

TCGA-ED-A82E-01A	60 years, female, asian, stage:iiia, alive, 408 days	2.8
TCGA-2Y-A9H6-01A	68 years, female, white, stage:i, alive, 357 days	2.8
TCGA-G3-AAV7-01A	38 years, male, asian, stage:ii, alive, 361 days	2.8
TCGA-BC-A110-01A	51 years, female, black or african american, dead, 2116 days	2.8
TCGA-DD-A1EL-01A	23 years, male, black or african american, stage:ii, dead, 415 days	2.8
TCGA-BC-A8YO-01A	66 years, female, white, stage:iiic, alive, 562 days	2.8
TCGA-CC-5263-01A	35 years, male, asian, stage:iiia, dead, 129 days	2.7
TCGA-DD-A11C-01A	69 years, male, white, stage:i, alive, 662 days	2.7
TCGA-BC-A10T-01A	76 years, male, white, dead, 837 days	2.7
TCGA-ED-A4XI-01A	58 years, male, asian, stage:ii, alive, 819 days	2.6
TCGA-DD-AADN-01A	59 years, male, asian, stage:i, alive, 898 days	2.6
TCGA-G3-A5SM-01A	58 years, male, white, stage:ii, alive, 520 days	2.6
TCGA-RG-A7D4-01A	69 years, male, black or african american, stage:ii, alive, 1098 days	2.6
TCGA-DD-A1EI-01A	46 years, male, asian, stage:i, alive, 183 days	2.6
TCGA-FV-A2QQ-01A	80 years, male, white, stage:i, alive, 729 days	2.6
TCGA-O8-A75V-01A	54 years, male, stage:i, alive, 538 days	2.6
TCGA-DD-AAE0-01A	45 years, female, asian, stage:iiia, alive, 555 days	2.6
TCGA-DD-AAE3-01A	50 years, male, asian, stage:i, alive, 566 days	2.5
TCGA-CC-A7II-01A	55 years, male, asian, stage:iiia, alive, 399 days	2.5
TCGA-CC-5258-01A	48 years, male, asian, stage:ii, dead, 129 days	2.5
TCGA-DD-AAEH-01A	73 years, male, asian, stage:i, alive, 784 days	2.5
TCGA-DD-A73G-01A	73 years, female, white, stage:i, alive, 3478 days	2.5
TCGA-DD-AACP-01A	65 years, male, asian, stage:i, alive, 415 days	2.5
TCGA-DD-AAC9-01A	51 years, male, asian, stage:i, alive, 347 days	2.5
TCGA-ED-A7PZ-01A	61 years, male, asian, stage:ii, alive, 6 days	2.5
TCGA-DD-A4NJ-01A	54 years, female, white, stage:ii, alive, 928 days	2.5
TCGA-G3-A7M6-01A	60 years, female, white, stage:i, alive, 632 days	2.5
TCGA-DD-AAE2-01A	51 years, male, asian, stage:i, alive, 638 days	2.4
TCGA-G3-A5SJ-01A	59 years, male, white, stage:i, alive, 698 days	2.4
TCGA-RC-A6M5-01A	20 years, female, white, stage:iva, alive, 15 days	2.4
TCGA-K7-A5RF-01A	64 years, male, white, stage:i, alive, 631 days	2.4
TCGA-2Y-A9GT-01A	51 years, male, white, stage:i, dead, 1624 days	2.4
TCGA-DD-A1EK-01A	64 years, female, white, stage:ivb, dead, 558 days	2.4
TCGA-UB-A7MA-01A	62 years, female, white, stage:ii, alive, 848 days	2.4
TCGA-FV-A2QR-01A	male, white, stage:i, dead, 581 days	2.3
TCGA-HP-A5N0-01A	88 years, female, dead, 1147 days	2.3
TCGA-G3-A25V-01A	68 years, male, white, stage:i, alive, 860 days	2.3
TCGA-2Y-A9H8-01A	85 years, female, white, dead, 633 days	2.3
TCGA-KR-A7K7-01A	61 years, female, white, stage:ii, alive, 951 days	2.3
TCGA-BC-A10W-01A	50 years, male, asian, dead, 91 days	2.3
TCGA-2Y-A9H2-01A	64 years, female, white, stage:i, alive, 1731 days	2.3
TCGA-FV-A3I0-01A	76 years, female, white, stage:ii, alive, 848 days	2.2
TCGA-DD-AADO-01A	55 years, male, asian, stage:i, alive, 453 days	2.2

TCGA-DD-A4NV-01A	61 years, male, white, stage:iiia, alive, 2398 days	2.2
TCGA-BC-A10Y-01A	76 years, male, white, dead, 711 days	2.2
TCGA-DD-A4NH-01A	65 years, female, white, stage:iiib, alive, 917 days	2.2
TCGA-DD-AAEI-01A	72 years, male, asian, stage:i, alive, 1531 days	2.2
TCGA-ED-A7XO-01A	29 years, male, asian, stage:iiia, alive, 427 days	2.2
TCGA-DD-A119-01A	40 years, male, asian, stage:iv, dead, 223 days	2.1
TCGA-DD-AAVS-01A	56 years, male, asian, stage:i, alive, 1823 days	2.1
TCGA-ED-A7XP-01A	53 years, female, asian, stage:ii, alive, 400 days	2.1
TCGA-DD-AAD3-01A	43 years, male, asian, stage:i, alive, 1295 days	2.1
TCGA-DD-AACG-01A	52 years, male, asian, stage:ii, dead, 469 days	2.1
TCGA-2Y-A9H1-01A	58 years, male, white, stage:i, dead, 1229 days	2.1
TCGA-DD-AACO-01A	40 years, male, asian, stage:i, alive, 1876 days	2.1
TCGA-5C-A9VG-01A	58 years, male, white, stage:ii, alive, 328 days	2.1
TCGA-DD-A1EF-01A	57 years, female, white, stage:i, dead, 394 days	2.1
TCGA-BD-A3ER-01A	62 years, male, white, stage:ii, alive, 1115 days	2.1
TCGA-EP-A2KA-01A	52 years, female, white, stage:iiia, dead, 627 days	2.1
TCGA-DD-AAD8-01A	73 years, female, asian, stage:i, alive, 1219 days	2.1
TCGA-ZS-A9CD-01A	73 years, male, white, stage:ii, dead, 1386 days	2.1
TCGA-UB-AA0U-01A	60 years, male, white, stage:ii, alive, 327 days	2.1
TCGA-BC-A216-01A	62 years, female, white, stage:iiia, alive, 1351 days	2.0
TCGA-ZP-A9D1-01A	56 years, female, white, alive, 21 days	2.0
TCGA-2Y-A9GV-01A	54 years, female, white, stage:i, dead, 2532 days	2.0
TCGA-GJ-A9DB-01A	68 years, male, white, stage:i, dead, 67 days	2.0
TCGA-DD-AAC8-01A	72 years, male, asian, stage:i, dead, 16 days	2.0
TCGA-DD-AADV-01A	50 years, male, asian, stage:i, alive, 574 days	2.0
TCGA-MI-A75H-01A	77 years, male, white, alive, 747 days	2.0
TCGA-5C-A9VH-01A	70 years, male, white, stage:i, alive, 322 days	2.0
TCGA-CC-A7IG-01A	47 years, male, asian, stage:ii, dead, 299 days	2.0
TCGA-BC-A69I-01A	69 years, male, white, stage:i, alive, 387 days	2.0
TCGA-DD-A1EJ-01A	71 years, female, white, stage:iiic, dead, 1005 days	2.0
TCGA-NI-A8LF-01A	74 years, male, white, stage:i, alive, 799 days	1.9
TCGA-BC-A10X-01A	52 years, female, white, stage:iiia, dead, 770 days	1.9
TCGA-DD-AADK-01A	68 years, female, asian, stage:ii, alive, 1049 days	1.9
TCGA-DD-AADW-01A	48 years, male, asian, stage:i, alive, 587 days	1.9
TCGA-2Y-A9GS-01A	58 years, male, white, dead, 724 days	1.9
TCGA-DD-AACU-01A	59 years, male, asian, stage:i, alive, 1567 days	1.9
TCGA-FV-A23B-01A	70 years, female, white, stage:ii, dead, 1852 days	1.9
TCGA-UB-AA0V-01A	69 years, female, white, stage:i, alive, 314 days	1.9
TCGA-DD-AACT-01A	69 years, female, asian, stage:i, alive, 1562 days	1.9
TCGA-DD-A118-01A	77 years, female, white, stage:ii, alive, 3437 days	1.9
TCGA-CC-A5UD-01A	45 years, male, asian, stage:iiia, dead, 304 days	1.9
TCGA-DD-AACF-01A	68 years, male, asian, stage:i, dead, 365 days	1.9
TCGA-DD-AACN-01A	32 years, male, asian, stage:i, alive, 1302 days	1.9

TCGA-2Y-A9HB-01A	66 years, male, stage:i, alive, 260 days	1.9
TCGA-DD-AAVX-01A	38 years, male, asian, stage:ii, alive, 1570 days	1.8
TCGA-DD-A115-01A	53 years, male, white, stage:iiia, dead, 2542 days	1.8
TCGA-DD-A3A5-01A	66 years, female, white, stage:iii, dead, 3125 days	1.8
TCGA-DD-AADS-01A	63 years, male, asian, stage:i, alive, 474 days	1.8
TCGA-G3-A3CH-01A	53 years, male, asian, stage:iiia, alive, 780 days	1.8
TCGA-BD-A2L6-01A	69 years, male, white, alive, 1363 days	1.8
TCGA-CC-A9FW-01A	68 years, male, asian, stage:iiia, alive, 248 days	1.7
TCGA-DD-A1EA-01A	68 years, male, asian, stage:ii, alive, 2415 days	1.7
TCGA-DD-AAEB-01A	60 years, male, asian, stage:i, alive, 478 days	1.7
TCGA-ZS-A9CF-01A	64 years, male, white, stage:ii, alive, 2412 days	1.7
TCGA-CC-A3MC-01A	54 years, male, asian, stage:iiia, alive, 363 days	1.7
TCGA-DD-A116-01A	68 years, male, asian, stage:iiia, dead, 1622 days	1.7
TCGA-BC-A3KF-01A	66 years, female, white, stage:i, alive, 8 days	1.7
TCGA-DD-AACJ-01A	75 years, male, asian, stage:ii, alive, 2102 days	1.7
TCGA-DD-AADP-01A	45 years, male, asian, stage:i, alive, 458 days	1.7
TCGA-EP-A26S-01A	70 years, male, white, stage:i, alive, 608 days	1.7
TCGA-ZP-A9D2-01A	51 years, male, white, dead, 765 days	1.7
TCGA-ES-A2HT-01A	54 years, male, black or african american, stage:i, dead, 438 days	1.7
TCGA-4R-AA8I-01A	66 years, male, white, stage:ii, dead, 262 days	1.7
TCGA-BC-A10Q-01A	72 years, female, white, dead, 1135 days	1.7
TCGA-DD-AADC-01A	53 years, male, asian, stage:i, dead, 425 days	1.7
TCGA-2Y-A9H4-01A	68 years, male, black or african american, stage:i, alive, 1452 days	1.6
TCGA-BW-A5NO-01A	50 years, male, black or african american, stage:iiia, alive, 20 days	1.6
TCGA-DD-AAVU-01A	46 years, male, asian, stage:ii, alive, 2202 days	1.6
TCGA-DD-A4NI-01A	67 years, male, white, stage:ii, alive, 816 days	1.6
TCGA-WQ-AB4B-01A	62 years, male, white, stage:ii, alive, 395 days	1.6
TCGA-KR-A7K0-01A	65 years, male, white, stage:i, dead, 65 days	1.6
TCGA-MI-A75E-01A	61 years, male, white, stage:iiic, alive, 507 days	1.6
TCGA-CC-5259-01A	60 years, female, asian, stage:iiic, alive, 250 days	1.6
TCGA-ZP-A9CY-01A	66 years, female, white, alive, 782 days	1.6
TCGA-BC-A10R-01A	66 years, female, white, dead, 308 days	1.6
TCGA-G3-AAV4-01A	83 years, female, white, stage:i, dead, 27 days	1.6
TCGA-DD-AAE7-01A	72 years, male, asian, stage:i, alive, 644 days	1.6
TCGA-CC-A3MB-01A	36 years, male, asian, stage:iiia, dead, 315 days	1.6
TCGA-DD-AACK-01A	70 years, male, asian, stage:i, alive, 9 days	1.6
TCGA-2Y-A9H5-01A	59 years, female, white, stage:i, dead, 555 days	1.5
TCGA-K7-A6G5-01A	66 years, male, white, stage:i, alive, 512 days	1.5
TCGA-G3-AAUZ-01A	48 years, male, stage:i, alive, 480 days	1.5
TCGA-BC-A217-01A	75 years, female, white, stage:ii, dead, 1397 days	1.5
TCGA-KR-A7K2-01A	64 years, male, white, stage:i, alive, 829 days	1.5
TCGA-G3-AAV1-01A	51 years, male, asian, stage:iiic, dead, 359 days	1.5
TCGA-DD-A73A-01A	71 years, male, white, stage:i, alive, 728 days	1.5

TCGA-WX-AA44-01A	64 years, female, white, stage:i, alive, 615 days	1.5
TCGA-DD-AADA-01A	66 years, female, asian, stage:i, alive, 1233 days	1.5
TCGA-ZP-A9CV-01A	59 years, male, white, dead, 1088 days	1.5
TCGA-DD-AADD-01A	51 years, male, asian, stage:i, alive, 1231 days	1.5
TCGA-DD-AAW2-01A	69 years, male, asian, stage:i, alive, 1855 days	1.5
TCGA-G3-A7M7-01A	65 years, male, white, stage:i, alive, 361 days	1.5
TCGA-EP-A2KC-01A	62 years, male, black or african american, stage:i, dead, 19 days	1.5
TCGA-CC-A8HS-01A	18 years, male, asian, stage:iiic, dead, 300 days	1.5
TCGA-DD-A4NG-01A	77 years, male, white, stage:iiia, dead, 802 days	1.5
TCGA-DD-A3A7-01A	67 years, male, stage:iiib, dead, 419 days	1.4
TCGA-CC-A123-01A	24 years, female, asian, stage:iiia, alive, 219 days	1.4
TCGA-DD-A3A1-01A	65 years, male, stage:iiia, dead, 233 days	1.4
TCGA-G3-AAV6-01A	53 years, female, white, stage:iiia, dead, 65 days	1.4
TCGA-2Y-A9H7-01A	81 years, female, white, stage:i, alive, 1168 days	1.4
TCGA-DD-A4NN-01A	56 years, female, white, stage:i, dead, 899 days	1.4
TCGA-CC-A7IH-01A	58 years, male, asian, stage:iiia, alive, 365 days	1.4
TCGA-XR-A8TG-01A	59 years, male, white, stage:i, alive, 898 days	1.4
TCGA-G3-A7M8-01A	31 years, male, asian, stage:i, alive, 430 days	1.4
TCGA-DD-A73C-01A	65 years, female, white, stage:iiia, alive, 701 days	1.4
TCGA-G3-A3CK-01A	61 years, male, asian, stage:i, alive, 585 days	1.4
TCGA-FV-A3R2-01A	75 years, male, white, stage:i, dead, 194 days	1.4
TCGA-DD-A1EB-01A	72 years, female, stage:i, alive, 2017 days	1.4
TCGA-G3-A25Z-01A	58 years, male, asian, stage:i, alive, 655 days	1.4
TCGA-DD-AACD-01A	48 years, male, asian, stage:i, dead, 381 days	1.4
TCGA-G3-A3CI-01A	71 years, male, white, stage:i, alive, 180 days	1.3
TCGA-DD-AAEA-01A	65 years, male, asian, stage:i, alive, 575 days	1.3
TCGA-DD-A4NO-01A	66 years, male, white, stage:i, alive, 2245 days	1.3
TCGA-DD-A1ED-01A	68 years, male, white, stage:i, alive, 2301 days	1.3
TCGA-NI-A4U2-01A	71 years, male, white, stage:iiia, dead, 1791 days	1.3
TCGA-DD-AACA-01A	65 years, male, asian, stage:i, alive, 2301 days	1.3
TCGA-2Y-A9H9-01A	70 years, male, white, stage:i, alive, 697 days	1.3
TCGA-DD-AADM-01A	58 years, male, asian, stage:ii, dead, 12 days	1.3
TCGA-DD-A4NK-01A	80 years, female, white, stage:iiia, dead, 1210 days	1.3
TCGA-DD-A1EE-01A	73 years, male, white, stage:iiia, dead, 349 days	1.3
TCGA-RC-A7SH-01A	42 years, male, asian, stage:ii, alive, 468 days	1.3
TCGA-DD-A4NQ-01A	60 years, male, white, stage:ii, dead, 373 days	1.3
TCGA-LG-A9QC-01A	48 years, male, white, stage:i, alive, 425 days	1.3
TCGA-EP-A2KB-01A	46 years, female, white, stage:i, dead, 596 days	1.2
TCGA-DD-AADF-01A	64 years, female, asian, stage:i, dead, 115 days	1.2
TCGA-DD-A1EH-01A	23 years, male, white, stage:iii, alive, 1495 days	1.2
TCGA-3K-AAZ8-01A	65 years, male, black or african american, stage:iiib, alive, 396 days	1.2
TCGA-DD-A73E-01A	66 years, male, white, stage:i, alive, 44 days	1.2
TCGA-CC-5264-01A	71 years, male, asian, stage:iiia, dead, 102 days	1.2

TCGA-WX-AA46-01A	62 years, male, white, stage:ii, alive, 756 days	1.2
TCGA-DD-AAE9-01A	69 years, male, asian, stage:i, alive, 722 days	1.2
TCGA-2Y-A9GZ-01A	82 years, female, white, stage:ii, dead, 848 days	1.2
TCGA-DD-AADY-01A	55 years, female, asian, stage:i, alive, 555 days	1.2
TCGA-G3-AAV2-01A	50 years, male, white, stage:i, alive, 372 days	1.2
TCGA-MI-A75C-01A	64 years, male, white, stage:i, alive, 291 days	1.1
TCGA-G3-AAV5-01A	67 years, male, white, stage:ii, alive, 354 days	1.1
TCGA-G3-A3CJ-01A	52 years, male, american indian or alaska native, stage:ii, alive, 594 days	1.1
TCGA-DD-AADG-01A	70 years, male, asian, stage:iiia, alive, 1145 days	1.1
TCGA-DD-A11D-01A	57 years, female, white, stage:i, dead, 1560 days	1.1
TCGA-G3-A3CG-01A	80 years, male, white, stage:i, alive, 673 days	1.1
TCGA-DD-AAE1-01A	52 years, male, asian, stage:i, alive, 552 days	1.1
TCGA-ES-A2HS-01A	80 years, male, white, stage:i, dead, 688 days	1.1
TCGA-DD-AAVP-01A	48 years, male, asian, stage:i, alive, 2752 days	1.1
TCGA-QA-A7B7-01A	48 years, male, black or african american, stage:ii, alive, 94 days	1.1
TCGA-WQ-A9G7-01A	female, white, alive, 30 days	1.1
TCGA-XR-A8TF-01A	74 years, male, white, stage:i, dead, 693 days	1.1
TCGA-DD-AA3A-01A	81 years, female, white, stage:i, dead, 410 days	1.1
TCGA-DD-A4NL-01A	46 years, male, white, stage:i, alive, 1711 days	1.1
TCGA-DD-AADQ-01A	59 years, male, asian, stage:ii, alive, 436 days	1.1
TCGA-CC-A9FS-01A	55 years, male, asian, stage:ii, alive, 211 days	1.1
TCGA-CC-A7IK-01A	59 years, male, asian, stage:iiia, dead, 262 days	1.1
TCGA-DD-A39W-01A	29 years, female, white, stage:iii, dead, 827 days	1.0
TCGA-ED-A459-01A	47 years, male, asian, stage:ii, alive, 910 days	1.0
TCGA-DD-AACW-01A	43 years, male, asian, stage:i, alive, 1424 days	1.0
TCGA-DD-AACV-01A	53 years, male, asian, stage:i, alive, 1531 days	1.0
TCGA-2Y-A9GU-01A	55 years, female, white, stage:i, alive, 1939 days	1.0
TCGA-DD-A4NF-01A	72 years, male, white, stage:i, alive, 942 days	1.0
TCGA-DD-AACX-01A	66 years, male, asian, stage:ii, alive, 170 days	1.0
TCGA-XR-A8TC-01A	43 years, female, white, stage:i, alive, 1339 days	1.0
TCGA-DD-AAW3-01A	69 years, male, asian, stage:i, alive, 1633 days	1.0
TCGA-RC-A7SF-01A	66 years, male, asian, stage:i, alive, 579 days	1.0
TCGA-DD-AAVZ-01A	38 years, male, asian, stage:i, alive, 1900 days	1.0
TCGA-G3-A25U-01A	63 years, female, asian, stage:i, alive, 1636 days	1.0
TCGA-DD-AAVY-01A	56 years, male, asian, stage:iiia, alive, 1970 days	1.0
TCGA-5R-AA1C-01A	57 years, male, white, stage:ii, alive, 520 days	1.0
TCGA-DD-AAEE-01A	55 years, male, asian, stage:i, alive, 810 days	1.0
TCGA-DD-A4NE-01A	75 years, female, white, stage:iiia, dead, 660 days	1.0
TCGA-CC-A7IF-01A	59 years, male, asian, stage:iiia, dead, 649 days	0.9
TCGA-RC-A7SB-01A	53 years, male, asian, stage:ii, alive, 588 days	0.9
TCGA-DD-AADJ-01A	70 years, female, asian, stage:i, alive, 1066 days	0.9
TCGA-UB-A7MB-01A	24 years, male, white, stage:ii, alive, 601 days	0.9
TCGA-T1-A6J8-01A	68 years, male, white, alive, 23 days	0.9

TCGA-DD-AADL-01A	58 years, male, asian, stage:i, alive, 636 days	0.9
TCGA-MI-A75I-01A	61 years, male, black or african american, alive, 630 days	0.9
TCGA-G3-A5SK-01A	58 years, male, white, stage:i, alive, 744 days	0.9
TCGA-EP-A12J-01A	63 years, male, black or african american, stage:i, alive, 570 days	0.9
TCGA-DD-AACY-01A	61 years, male, asian, stage:i, alive, 1450 days	0.9
TCGA-LG-A9QD-01A	68 years, male, white, stage:iiia, alive, 366 days	0.9
TCGA-MI-A75G-01A	63 years, male, white, stage:ii, alive, 698 days	0.9
TCGA-FV-A496-01A	84 years, female, white, stage:i, alive, 10 days	0.9
TCGA-DD-AAW1-01A	55 years, male, asian, stage:iiia, alive, 1989 days	0.9
TCGA-LG-A6GG-01A	79 years, female, white, stage:ii, alive, 387 days	0.9
TCGA-RC-A7SK-01A	59 years, male, asian, stage:i, alive, 472 days	0.8
TCGA-RC-A6M4-01A	74 years, female, white, stage:iiia, alive, 22 days	0.8
TCGA-G3-AAV0-01A	58 years, male, asian, stage:i, alive, 476 days	0.8
TCGA-DD-A39Y-01A	67 years, male, asian, stage:i, dead, 171 days	0.8
TCGA-DD-AACS-01A	39 years, male, asian, stage:i, alive, 1804 days	0.8
TCGA-BC-A3KG-01A	68 years, female, white, stage:ii, alive, 680 days	0.8
TCGA-DD-A73D-01A	68 years, female, white, stage:ii, alive, 693 days	0.8
TCGA-DD-A73B-01A	72 years, female, white, stage:i, dead, 283 days	0.8
TCGA-DD-A4NP-01A	32 years, male, white, stage:i, alive, 3308 days	0.8
TCGA-DD-A11B-01A	73 years, male, white, stage:i, dead, 14 days	0.8
TCGA-DD-AACE-01A	62 years, male, asian, stage:i, alive, 2184 days	0.8
TCGA-UB-A7MC-01A	59 years, male, white, stage:iiia, alive, 500 days	0.8
TCGA-DD-AAEG-01A	59 years, female, asian, stage:i, alive, 719 days	0.8
TCGA-DD-A39Z-01A	43 years, female, stage:ii, dead, 601 days	0.8
TCGA-ZP-A9D4-01A	64 years, female, white, alive, 395 days	0.8
TCGA-DD-AAED-01A	51 years, male, asian, stage:i, alive, 763 days	0.8
TCGA-CC-A8HU-01A	39 years, female, asian, stage:iiia, dead, 344 days	0.8
TCGA-G3-A5SL-01A	70 years, male, white, stage:ii, alive, 621 days	0.8
TCGA-CC-A8HV-01A	51 years, female, asian, stage:ii, dead, 279 days	0.8
TCGA-MR-A520-01A	58 years, male, white, stage:i, alive, 229 days	0.8
TCGA-ZS-A9CG-01A	55 years, male, white, stage:ii, alive, 341 days	0.8
TCGA-DD-AAD0-01A	73 years, female, asian, stage:i, alive, 137 days	0.7
TCGA-DD-AADU-01A	60 years, male, asian, stage:ii, alive, 554 days	0.7
TCGA-ZP-A9D0-01A	67 years, female, black or african american, alive, 1091 days	0.7
TCGA-BC-A10U-01A	69 years, male, white, dead, 837 days	0.7
TCGA-BC-A10Z-01A	62 years, female, white, stage:i, dead, 34 days	0.7
TCGA-WJ-A86L-01A	68 years, female, white, stage:i, alive, 345 days	0.7
TCGA-DD-AACQ-01A	50 years, male, asian, stage:ii, dead, 432 days	0.7
TCGA-ED-A66Y-01A	51 years, female, asian, stage:iiia, dead, 296 days	0.7
TCGA-G3-A6UC-01A	65 years, male, white, stage:iiib, alive, 671 days	0.6
TCGA-DD-A3A8-01A	75 years, male, white, stage:ii, dead, 11 days	0.6
TCGA-XR-A8TE-01A	16 years, male, white, stage:iiia, alive, 925 days	0.6
TCGA-RC-A7S9-01A	47 years, female, asian, stage:i, alive, 640 days	0.6

TCGA-G3-A5SI-01A	44 years, male, asian, stage:ii, dead, 768 days	0.6
TCGA-ED-A7PY-01A	20 years, female, asian, stage:ii, alive, 390 days	0.6
TCGA-DD-A3A4-01A	37 years, male, white, stage:iiia, dead, 612 days	0.6
TCGA-DD-A3A2-01A	76 years, female, white, stage:i, dead, 2131 days	0.5
TCGA-DD-AAD6-01A	66 years, male, asian, stage:iiia, alive, 672 days	0.5
TCGA-WX-AA47-01A	33 years, female, white, stage:iiia, dead, 556 days	0.4
TCGA-DD-AAE6-01A	59 years, female, asian, stage:i, alive, 141 days	0.4
TCGA-BC-A5W4-01A	69 years, male, white, stage:iiia, dead, 547 days	0.3
TCGA-ZS-A9CE-01A	79 years, female, white, stage:ii, alive, 1241 days	0.3

TRPV3 RNA expression (N=365)

Sample	Description	FPKM
TCGA-DD-AA3A-01A	81 years, female, white, stage:i, dead, 410 days	1.6
TCGA-EP-A3RK-01A	73 years, male, white, stage:iiia, alive, 363 days	0.9
TCGA-ED-A66Y-01A	51 years, female, asian, stage:iiia, dead, 296 days	0.7
TCGA-2Y-A9HA-01A	70 years, male, white, stage:ii, dead, 36 days	0.7
TCGA-DD-AACK-01A	70 years, male, asian, stage:i, alive, 9 days	0.6
TCGA-UB-AA0V-01A	69 years, female, white, stage:i, alive, 314 days	0.6
TCGA-DD-AACO-01A	40 years, male, asian, stage:i, alive, 1876 days	0.6
TCGA-BD-A3ER-01A	62 years, male, white, stage:ii, alive, 1115 days	0.6
TCGA-ZP-A9D2-01A	51 years, male, white, dead, 765 days	0.5
TCGA-WQ-A9G7-01A	female, white, alive, 30 days	0.5
TCGA-WJ-A86L-01A	68 years, female, white, stage:i, alive, 345 days	0.5
TCGA-ED-A7PX-01A	48 years, female, asian, stage:ii, alive, 6 days	0.5
TCGA-DD-AAEI-01A	72 years, male, asian, stage:i, alive, 1531 days	0.5
TCGA-G3-A7M9-01A	70 years, male, white, stage:iiib, dead, 56 days	0.4
TCGA-DD-A1EL-01A	23 years, male, black or african american, stage:ii, dead, 415 days	0.4
TCGA-GJ-A9DB-01A	68 years, male, white, stage:i, dead, 67 days	0.4
TCGA-2Y-A9GW-01A	64 years, male, white, stage:i, dead, 1271 days	0.4
TCGA-2Y-A9GU-01A	55 years, female, white, stage:i, alive, 1939 days	0.4
TCGA-NI-A8LF-01A	74 years, male, white, stage:i, alive, 799 days	0.4
TCGA-5C-AAPD-01A	61 years, male, asian, stage:ii, alive, 20 days	0.4
TCGA-CC-A8HS-01A	18 years, male, asian, stage:iiic, dead, 300 days	0.4
TCGA-DD-A4NO-01A	66 years, male, white, stage:i, alive, 2245 days	0.4
TCGA-2Y-A9H4-01A	68 years, male, black or african american, stage:i, alive, 1452 days	0.4
TCGA-DD-AAD8-01A	73 years, female, asian, stage:i, alive, 1219 days	0.4
TCGA-2Y-A9GS-01A	58 years, male, white, dead, 724 days	0.4
TCGA-DD-A113-01A	55 years, female, white, stage:ii, alive, 2425 days	0.4
TCGA-G3-A3CH-01A	53 years, male, asian, stage:iiia, alive, 780 days	0.3
TCGA-BW-A5NO-01A	50 years, male, black or african american, stage:iiia, alive, 20 days	0.3
TCGA-HP-A5MZ-01A	62 years, male, stage:i, dead, 91 days	0.3
TCGA-DD-AAEH-01A	73 years, male, asian, stage:i, alive, 784 days	0.3

TCGA-RC-A7SK-01A	59 years, male, asian, stage:i, alive, 472 days	0.3
TCGA-WX-AA44-01A	64 years, female, white, stage:i, alive, 615 days	0.3
TCGA-FV-A2QQ-01A	80 years, male, white, stage:i, alive, 729 days	0.3
TCGA-2Y-A9GX-01A	68 years, male, white, stage:i, alive, 2442 days	0.3
TCGA-4R-AA8I-01A	66 years, male, white, stage:ii, dead, 262 days	0.3
TCGA-DD-A3A1-01A	65 years, male, stage:iiia, dead, 233 days	0.3
TCGA-5R-AA1C-01A	57 years, male, white, stage:ii, alive, 520 days	0.3
TCGA-DD-A11A-01A	67 years, male, black or african american, stage:i, alive, 79 days	0.3
TCGA-2Y-A9H6-01A	68 years, female, white, stage:i, alive, 357 days	0.3
TCGA-CC-A3MA-01A	61 years, male, asian, stage:iiia, dead, 303 days	0.3
TCGA-RC-A7SB-01A	53 years, male, asian, stage:ii, alive, 588 days	0.3
TCGA-BC-A10Z-01A	62 years, female, white, stage:i, dead, 34 days	0.3
TCGA-FV-A3I0-01A	76 years, female, white, stage:ii, alive, 848 days	0.3
TCGA-G3-A3CK-01A	61 years, male, asian, stage:i, alive, 585 days	0.2
TCGA-ZS-A9CF-01A	64 years, male, white, stage:ii, alive, 2412 days	0.2
TCGA-DD-AAE0-01A	45 years, female, asian, stage:iiia, alive, 555 days	0.2
TCGA-G3-A5SK-01A	58 years, male, white, stage:i, alive, 744 days	0.2
TCGA-ES-A2HS-01A	80 years, male, white, stage:i, dead, 688 days	0.2
TCGA-KR-A7K0-01A	65 years, male, white, stage:i, dead, 65 days	0.2
TCGA-K7-A5RG-01A	66 years, male, black or african american, stage:i, alive, 519 days	0.2
TCGA-DD-AAVY-01A	56 years, male, asian, stage:iiia, alive, 1970 days	0.2
TCGA-DD-A73E-01A	66 years, male, white, stage:i, alive, 44 days	0.2
TCGA-DD-AAW0-01A	54 years, male, asian, stage:i, alive, 2015 days	0.2
TCGA-DD-AAEA-01A	65 years, male, asian, stage:i, alive, 575 days	0.2
TCGA-UB-A7MD-01A	67 years, male, black or african american, stage:i, dead, 52 days	0.2
TCGA-XR-A8TD-01A	49 years, female, white, stage:iiib, alive, 1030 days	0.2
TCGA-BC-A112-01A	80 years, male, white, dead, 153 days	0.2
TCGA-DD-AADM-01A	58 years, male, asian, stage:ii, dead, 12 days	0.2
TCGA-5R-AAAM-01A	65 years, female, white, stage:ii, dead, 46 days	0.2
TCGA-G3-A25V-01A	68 years, male, white, stage:i, alive, 860 days	0.2
TCGA-BC-A10Y-01A	76 years, male, white, dead, 711 days	0.2
TCGA-CC-A7IE-01A	57 years, male, asian, stage:iiia, dead, 217 days	0.2
TCGA-DD-AACX-01A	66 years, male, asian, stage:ii, alive, 170 days	0.2
TCGA-DD-A4NH-01A	65 years, female, white, stage:iiib, alive, 917 days	0.2
TCGA-DD-AADW-01A	48 years, male, asian, stage:i, alive, 587 days	0.2
TCGA-DD-A39X-01A	78 years, female, white, stage:i, dead, 1694 days	0.2
TCGA-UB-A7MA-01A	62 years, female, white, stage:ii, alive, 848 days	0.2
TCGA-DD-AAE9-01A	69 years, male, asian, stage:i, alive, 722 days	0.2
TCGA-FV-A3R3-01A	38 years, female, white, stage:i, dead, 366 days	0.2
TCGA-CC-A7IH-01A	58 years, male, asian, stage:iiia, alive, 365 days	0.2
TCGA-K7-AAU7-01A	61 years, male, white, stage:ii, alive, 359 days	0.2
TCGA-DD-AAEE-01A	55 years, male, asian, stage:i, alive, 810 days	0.1
TCGA-FV-A3I1-01A	female, white, stage:ii, dead, 247 days	0.1

TCGA-2Y-A9HB-01A	66 years, male, stage:i, alive, 260 days	0.1
TCGA-G3-AAUZ-01A	48 years, male, stage:i, alive, 480 days	0.1
TCGA-G3-AAV4-01A	83 years, female, white, stage:i, dead, 27 days	0.1
TCGA-DD-AACP-01A	65 years, male, asian, stage:i, alive, 415 days	0.1
TCGA-ED-A82E-01A	60 years, female, asian, stage:iiia, alive, 408 days	0.1
TCGA-DD-AAEG-01A	59 years, female, asian, stage:i, alive, 719 days	0.1
TCGA-CC-5261-01A	44 years, male, asian, stage:ii, dead, 97 days	0.1
TCGA-DD-AACJ-01A	75 years, male, asian, stage:ii, alive, 2102 days	0.1
TCGA-BC-4072-01B	74 years, female, white, stage:iiia, dead, 1490 days	0.1
TCGA-BC-A217-01A	75 years, female, white, stage:ii, dead, 1397 days	0.1
TCGA-MI-A75E-01A	61 years, male, white, stage:iiic, alive, 507 days	0.1
TCGA-DD-A4NV-01A	61 years, male, white, stage:iiia, alive, 2398 days	0.1
TCGA-G3-A5SM-01A	58 years, male, white, stage:ii, alive, 520 days	0.1
TCGA-EP-A3JL-01A	76 years, male, white, stage:i, alive, 303 days	0.1
TCGA-G3-A7M8-01A	31 years, male, asian, stage:i, alive, 430 days	0.1
TCGA-HP-A5N0-01A	88 years, female, dead, 1147 days	0.1
TCGA-K7-A5RF-01A	64 years, male, white, stage:i, alive, 631 days	0.1
TCGA-ED-A4XI-01A	58 years, male, asian, stage:ii, alive, 819 days	0.1
TCGA-DD-A118-01A	77 years, female, white, stage:ii, alive, 3437 days	0.1
TCGA-CC-A8HV-01A	51 years, female, asian, stage:ii, dead, 279 days	0.1
TCGA-XR-A8TG-01A	59 years, male, white, stage:i, alive, 898 days	0.1
TCGA-UB-A7MC-01A	59 years, male, white, stage:iiia, alive, 500 days	0.1
TCGA-ED-A627-01A	74 years, male, white, stage:i, alive, 423 days	0.1
TCGA-DD-A39W-01A	29 years, female, white, stage:iii, dead, 827 days	0.1
TCGA-BD-A3EP-01A	76 years, female, black or african american, stage:i, alive, 409 days	0.1
TCGA-ZS-A9CE-01A	79 years, female, white, stage:ii, alive, 1241 days	0.1
TCGA-2Y-A9H2-01A	64 years, female, white, stage:i, alive, 1731 days	0.1
TCGA-DD-A1EA-01A	68 years, male, asian, stage:ii, alive, 2415 days	0.1
TCGA-T1-A6J8-01A	68 years, male, white, alive, 23 days	0.1
TCGA-G3-AAV2-01A	50 years, male, white, stage:i, alive, 372 days	0.1
TCGA-ED-A5KG-01A	60 years, female, asian, stage:ii, alive, 854 days	0.1
TCGA-ED-A459-01A	47 years, male, asian, stage:ii, alive, 910 days	0.1
TCGA-DD-AACT-01A	69 years, female, asian, stage:i, alive, 1562 days	0.1
TCGA-DD-AADV-01A	50 years, male, asian, stage:i, alive, 574 days	0.1
TCGA-G3-A6UC-01A	65 years, male, white, stage:iiib, alive, 671 days	0.1
TCGA-MI-A75I-01A	61 years, male, black or african american, alive, 630 days	0.1
TCGA-2Y-A9H0-01A	49 years, male, white, stage:iiia, alive, 3675 days	0.1
TCGA-WX-AA47-01A	33 years, female, white, stage:iiia, dead, 556 days	0.1
TCGA-PD-A5DF-01A	58 years, female, white, stage:iiib, dead, 639 days	0.1
TCGA-DD-A1EE-01A	73 years, male, white, stage:iiia, dead, 349 days	0.1
TCGA-DD-A73C-01A	65 years, female, white, stage:iiia, alive, 701 days	0.1
TCGA-ZP-A9D1-01A	56 years, female, white, alive, 21 days	0.1
TCGA-DD-A3A2-01A	76 years, female, white, stage:i, dead, 2131 days	0.1

TCGA-BC-A10R-01A	66 years, female, white, dead, 308 days	0.1
TCGA-G3-A3CI-01A	71 years, male, white, stage:i, alive, 180 days	0.1
TCGA-ED-A97K-01A	54 years, male, asian, stage:iiia, alive, 6 days	0.1
TCGA-DD-AACQ-01A	50 years, male, asian, stage:ii, dead, 432 days	0.1
TCGA-G3-A5SL-01A	70 years, male, white, stage:ii, alive, 621 days	0.1
TCGA-DD-A4NI-01A	67 years, male, white, stage:ii, alive, 816 days	0.1
TCGA-BC-A8YO-01A	66 years, female, white, stage:iiic, alive, 562 days	0.1
TCGA-ED-A8O6-01A	50 years, female, asian, stage:iiia, dead, 56 days	0.1
TCGA-YA-A8S7-01A	69 years, male, white, stage:iiia, dead, 412 days	0.1
TCGA-DD-A39V-01A	77 years, male, white, stage:ii, dead, 643 days	0.1
TCGA-DD-AACY-01A	61 years, male, asian, stage:i, alive, 1450 days	0.1
TCGA-DD-A4NE-01A	75 years, female, white, stage:iiia, dead, 660 days	0.1
TCGA-DD-AAED-01A	51 years, male, asian, stage:i, alive, 763 days	0.1
TCGA-RC-A6M6-01A	75 years, male, white, stage:ii, alive, 9 days	0.1
TCGA-DD-AACU-01A	59 years, male, asian, stage:i, alive, 1567 days	0.1
TCGA-3K-AAZ8-01A	65 years, male, black or african american, stage:iiib, alive, 396 days	0.1
TCGA-DD-A4NS-01A	62 years, female, white, stage:i, dead, 2456 days	0.1
TCGA-EP-A2KB-01A	46 years, female, white, stage:i, dead, 596 days	0.1
TCGA-DD-A3A6-01A	72 years, female, white, stage:ii, dead, 3258 days	0.1
TCGA-DD-AADU-01A	60 years, male, asian, stage:ii, alive, 554 days	0.1
TCGA-DD-A4NL-01A	46 years, male, white, stage:i, alive, 1711 days	0.1
TCGA-DD-AADB-01A	51 years, male, asian, stage:i, alive, 1242 days	0.0
TCGA-GJ-A3OU-01A	59 years, male, white, stage:i, alive, 879 days	0.0
TCGA-ED-A66X-01A	35 years, male, asian, stage:iiia, alive, 406 days	0.0
TCGA-BC-A10X-01A	52 years, female, white, stage:iiia, dead, 770 days	0.0
TCGA-2Y-A9GV-01A	54 years, female, white, stage:i, dead, 2532 days	0.0
TCGA-DD-AAD5-01A	54 years, male, asian, stage:i, alive, 1345 days	0.0
TCGA-BC-A10U-01A	69 years, male, white, dead, 837 days	0.0
TCGA-DD-AAVW-01A	35 years, male, asian, stage:i, alive, 2317 days	0.0
TCGA-DD-AAVU-01A	46 years, male, asian, stage:ii, alive, 2202 days	0.0
TCGA-DD-A4NR-01A	85 years, female, white, stage:i, dead, 9 days	0.0
TCGA-CC-5258-01A	48 years, male, asian, stage:ii, dead, 129 days	0.0
TCGA-DD-AAW2-01A	69 years, male, asian, stage:i, alive, 1855 days	0.0
TCGA-O8-A75V-01A	54 years, male, stage:i, alive, 538 days	0.0
TCGA-CC-A7IK-01A	59 years, male, asian, stage:iiia, dead, 262 days	0.0
TCGA-ED-A8O5-01A	59 years, female, asian, stage:iiia, alive, 406 days	0.0
TCGA-UB-A7ME-01A	51 years, male, asian, stage:i, alive, 486 days	0.0
TCGA-CC-A3MC-01A	54 years, male, asian, stage:iiia, alive, 363 days	0.0
TCGA-FV-A4ZP-01A	78 years, male, white, stage:iiia, dead, 2486 days	0.0
TCGA-ED-A7XP-01A	53 years, female, asian, stage:ii, alive, 400 days	0.0
TCGA-2Y-A9H5-01A	59 years, female, white, stage:i, dead, 555 days	0.0
TCGA-BC-A10Q-01A	72 years, female, white, dead, 1135 days	0.0
TCGA-DD-A1EI-01A	46 years, male, asian, stage:i, alive, 183 days	0.0

TCGA-RC-A7SH-01A	42 years, male, asian, stage:ii, alive, 468 days	0.0
TCGA-CC-A8HT-01A	74 years, male, asian, stage:iiia, dead, 140 days	0.0
TCGA-CC-A8HU-01A	39 years, female, asian, stage:iiia, dead, 344 days	0.0
TCGA-DD-AACN-01A	32 years, male, asian, stage:i, alive, 1302 days	0.0
TCGA-ED-A7XO-01A	29 years, male, asian, stage:iiia, alive, 427 days	0.0
TCGA-DD-A3A9-01A	64 years, female, white, stage:ivb, dead, 931 days	0.0
TCGA-CC-A5UE-01A	48 years, male, asian, stage:iiib, dead, 272 days	0.0
TCGA-CC-A3M9-01A	45 years, male, asian, stage:iiia, dead, 300 days	0.0
TCGA-DD-AAEB-01A	60 years, male, asian, stage:i, alive, 478 days	0.0
TCGA-DD-AAVR-01A	44 years, male, asian, stage:i, alive, 2513 days	0.0
TCGA-DD-AAE2-01A	51 years, male, asian, stage:i, alive, 638 days	0.0
TCGA-ZP-A9CY-01A	66 years, female, white, alive, 782 days	0.0
TCGA-DD-A4ND-01A	56 years, female, white, stage:i, alive, 2746 days	0.0
TCGA-DD-AACE-01A	62 years, male, asian, stage:i, alive, 2184 days	0.0
TCGA-DD-AAD2-01A	66 years, male, asian, stage:i, alive, 658 days	0.0
TCGA-BC-A10W-01A	50 years, male, asian, dead, 91 days	0.0
TCGA-EP-A2KA-01A	52 years, female, white, stage:iiia, dead, 627 days	0.0
TCGA-BC-4073-01B	73 years, male, white, stage:iiia, alive, 849 days	0.0
TCGA-DD-A4NF-01A	72 years, male, white, stage:i, alive, 942 days	0.0
TCGA-BC-A216-01A	62 years, female, white, stage:iiia, alive, 1351 days	0.0
TCGA-DD-A73A-01A	71 years, male, white, stage:i, alive, 728 days	0.0
TCGA-DD-AAD1-01A	51 years, female, asian, stage:i, alive, 564 days	0.0
TCGA-DD-A3A3-01A	45 years, male, asian, stage:i, dead, 535 days	0.0
TCGA-BC-A10S-01A	81 years, male, white, dead, 1423 days	0.0
TCGA-DD-AADF-01A	64 years, female, asian, stage:i, dead, 115 days	0.0
TCGA-DD-A4NN-01A	56 years, female, white, stage:i, dead, 899 days	0.0
TCGA-GJ-A6C0-01A	75 years, female, white, stage:ii, dead, 31 days	0.0
TCGA-KR-A7K2-01A	64 years, male, white, stage:i, alive, 829 days	0.0
TCGA-DD-AAVZ-01A	38 years, male, asian, stage:i, alive, 1900 days	0.0
TCGA-BC-A10T-01A	76 years, male, white, dead, 837 days	0.0
TCGA-DD-AADJ-01A	70 years, female, asian, stage:i, alive, 1066 days	0.0
TCGA-G3-AAV6-01A	53 years, female, white, stage:iiia, dead, 65 days	0.0
TCGA-G3-A25Y-01A	52 years, female, asian, stage:i, dead, 452 days	0.0
TCGA-RC-A6M5-01A	20 years, female, white, stage:iva, alive, 15 days	0.0
TCGA-CC-A5UD-01A	45 years, male, asian, stage:iiia, dead, 304 days	0.0
TCGA-5R-AA1D-01A	17 years, female, white, stage:iiia, alive, 449 days	0.0
TCGA-DD-AAEK-01A	51 years, male, asian, stage:ii, alive, 1067 days	0.0
TCGA-EP-A2KC-01A	62 years, male, black or african american, stage:i, dead, 19 days	0.0
TCGA-DD-AADG-01A	70 years, male, asian, stage:iiia, alive, 1145 days	0.0
TCGA-G3-A25T-01A	45 years, female, white, stage:iiia, alive, 1553 days	0.0
TCGA-MR-A8JO-01A	34 years, male, white, stage:i, alive, 330 days	0.0
TCGA-DD-A114-01A	42 years, male, black or african american, stage:ii, dead, 1149 days	0.0
TCGA-CC-A9FS-01A	55 years, male, asian, stage:ii, alive, 211 days	0.0

TCGA-DD-A11B-01A	73 years, male, white, stage:i, dead, 14 days	0.0
TCGA-ED-A7PZ-01A	61 years, male, asian, stage:ii, alive, 6 days	0.0
TCGA-DD-A1ED-01A	68 years, male, white, stage:i, alive, 2301 days	0.0
TCGA-DD-AADK-01A	68 years, female, asian, stage:ii, alive, 1049 days	0.0
TCGA-FV-A4ZQ-01A	52 years, male, white, stage:i, alive, 12 days	0.0
TCGA-DD-A4NJ-01A	54 years, female, white, stage:ii, alive, 928 days	0.0
TCGA-DD-A1EF-01A	57 years, female, white, stage:i, dead, 394 days	0.0
TCGA-DD-AAVP-01A	48 years, male, asian, stage:i, alive, 2752 days	0.0
TCGA-CC-A7IJ-01A	56 years, male, asian, stage:ii, alive, 382 days	0.0
TCGA-DD-AAC9-01A	51 years, male, asian, stage:i, alive, 347 days	0.0
TCGA-DD-A1EK-01A	64 years, female, white, stage:ivb, dead, 558 days	0.0
TCGA-LG-A9QC-01A	48 years, male, white, stage:i, alive, 425 days	0.0
TCGA-CC-A1HT-01A	50 years, male, asian, stage:iiia, dead, 101 days	0.0
TCGA-DD-AACL-01A	66 years, female, asian, stage:i, dead, 107 days	0.0
TCGA-BC-A3KG-01A	68 years, female, white, stage:ii, alive, 680 days	0.0
TCGA-BC-A110-01A	51 years, female, black or african american, dead, 2116 days	0.0
TCGA-ZP-A9CV-01A	59 years, male, white, dead, 1088 days	0.0
TCGA-CC-5263-01A	35 years, male, asian, stage:iiia, dead, 129 days	0.0
TCGA-MI-A75H-01A	77 years, male, white, alive, 747 days	0.0
TCGA-CC-5259-01A	60 years, female, asian, stage:iiic, alive, 250 days	0.0
TCGA-DD-A73D-01A	68 years, female, white, stage:ii, alive, 693 days	0.0
TCGA-RG-A7D4-01A	69 years, male, black or african american, stage:ii, alive, 1098 days	0.0
TCGA-G3-AAV7-01A	38 years, male, asian, stage:ii, alive, 361 days	0.0
TCGA-DD-AACB-01A	74 years, female, asian, stage:i, alive, 2324 days	0.0
TCGA-DD-AAE4-01A	49 years, female, asian, stage:i, alive, 608 days	0.0
TCGA-FV-A2QR-01A	male, white, stage:i, dead, 581 days	0.0
TCGA-DD-AAVQ-01A	38 years, male, asian, stage:i, alive, 2728 days	0.0
TCGA-UB-AA0U-01A	60 years, male, white, stage:ii, alive, 327 days	0.0
TCGA-DD-A4NQ-01A	60 years, male, white, stage:ii, dead, 373 days	0.0
TCGA-ES-A2HT-01A	54 years, male, black or african american, stage:i, dead, 438 days	0.0
TCGA-DD-AAVS-01A	56 years, male, asian, stage:i, alive, 1823 days	0.0
TCGA-DD-AAVV-01A	56 years, male, asian, stage:ii, alive, 2455 days	0.0
TCGA-CC-A5UC-01A	63 years, male, asian, stage:iiia, dead, 347 days	0.0
TCGA-ED-A7PY-01A	20 years, female, asian, stage:ii, alive, 390 days	0.0
TCGA-DD-AACC-01A	61 years, male, asian, stage:i, dead, 1685 days	0.0
TCGA-CC-A3MB-01A	36 years, male, asian, stage:iiia, dead, 315 days	0.0
TCGA-G3-A7M6-01A	60 years, female, white, stage:i, alive, 632 days	0.0
TCGA-DD-A4NA-01A	67 years, female, white, stage:iiic, alive, 1008 days	0.0
TCGA-NI-A4U2-01A	71 years, male, white, stage:iiia, dead, 1791 days	0.0
TCGA-G3-AAV1-01A	51 years, male, asian, stage:iiic, dead, 359 days	0.0
TCGA-DD-AADL-01A	58 years, male, asian, stage:i, alive, 636 days	0.0
TCGA-G3-A7M5-01A	76 years, male, asian, stage:i, alive, 447 days	0.0
TCGA-DD-A3A8-01A	75 years, male, white, stage:ii, dead, 11 days	0.0

TCGA-MR-A520-01A	58 years, male, white, stage:i, alive, 229 days	0.0
TCGA-BC-A3KF-01A	66 years, female, white, stage:i, alive, 8 days	0.0
TCGA-2Y-A9H3-01A	45 years, male, white, stage:ii, alive, 1516 days	0.0
TCGA-DD-AADC-01A	53 years, male, asian, stage:i, dead, 425 days	0.0
TCGA-XR-A8TE-01A	16 years, male, white, stage:iiia, alive, 925 days	0.0
TCGA-2Y-A9GY-01A	64 years, female, white, stage:ii, dead, 757 days	0.0
TCGA-DD-A1EG-01A	77 years, male, white, stage:i, dead, 1372 days	0.0
TCGA-DD-AADP-01A	45 years, male, asian, stage:i, alive, 458 days	0.0
TCGA-DD-AAC8-01A	72 years, male, asian, stage:i, dead, 16 days	0.0
TCGA-DD-A4NB-01A	25 years, male, white, stage:i, alive, 989 days	0.0
TCGA-2Y-A9H8-01A	85 years, female, white, dead, 633 days	0.0
TCGA-CC-A7IF-01A	59 years, male, asian, stage:iiia, dead, 649 days	0.0
TCGA-BC-A5W4-01A	69 years, male, white, stage:iiia, dead, 547 days	0.0
TCGA-LG-A9QD-01A	68 years, male, white, stage:iiia, alive, 366 days	0.0
TCGA-CC-5260-01A	61 years, female, asian, stage:iiic, dead, 87 days	0.0
TCGA-DD-A1EH-01A	23 years, male, white, stage:iii, alive, 1495 days	0.0
TCGA-DD-A73G-01A	73 years, female, white, stage:i, alive, 3478 days	0.0
TCGA-DD-AACI-01A	69 years, male, asian, stage:ii, alive, 1618 days	0.0
TCGA-FV-A23B-01A	70 years, female, white, stage:ii, dead, 1852 days	0.0
TCGA-DD-A116-01A	68 years, male, asian, stage:iiia, dead, 1622 days	0.0
TCGA-DD-A119-01A	40 years, male, asian, stage:iv, dead, 223 days	0.0
TCGA-DD-AACG-01A	52 years, male, asian, stage:ii, dead, 469 days	0.0
TCGA-QA-A7B7-01A	48 years, male, black or african american, stage:ii, alive, 94 days	0.0
TCGA-UB-A7MF-01A	57 years, male, white, stage:iiia, dead, 214 days	0.0
TCGA-G3-AAV3-01A	58 years, female, white, stage:ii, alive, 412 days	0.0
TCGA-2Y-A9GZ-01A	82 years, female, white, stage:ii, dead, 848 days	0.0
TCGA-CC-A9FW-01A	68 years, male, asian, stage:iiia, alive, 248 days	0.0
TCGA-DD-A11D-01A	57 years, female, white, stage:i, dead, 1560 days	0.0
TCGA-5C-A9VH-01A	70 years, male, white, stage:i, alive, 322 days	0.0
TCGA-UB-A7MB-01A	24 years, male, white, stage:ii, alive, 601 days	0.0
TCGA-CC-5264-01A	71 years, male, asian, stage:iiia, dead, 102 days	0.0
TCGA-ZP-A9D0-01A	67 years, female, black or african american, alive, 1091 days	0.0
TCGA-DD-AACW-01A	43 years, male, asian, stage:i, alive, 1424 days	0.0
TCGA-XR-A8TF-01A	74 years, male, white, stage:i, dead, 693 days	0.0
TCGA-DD-A73F-01A	77 years, female, white, stage:i, alive, 1085 days	0.0
TCGA-BC-A69H-01A	64 years, male, white, stage:ii, alive, 444 days	0.0
TCGA-ZP-A9CZ-01A	72 years, male, asian, alive, 706 days	0.0
TCGA-FV-A496-01A	84 years, female, white, stage:i, alive, 10 days	0.0
TCGA-G3-A7M7-01A	65 years, male, white, stage:i, alive, 361 days	0.0
TCGA-G3-A5SJ-01A	59 years, male, white, stage:i, alive, 698 days	0.0
TCGA-5C-A9VG-01A	58 years, male, white, stage:ii, alive, 328 days	0.0
TCGA-DD-AACV-01A	53 years, male, asian, stage:i, alive, 1531 days	0.0
TCGA-DD-A4NG-01A	77 years, male, white, stage:iiia, dead, 802 days	0.0

TCGA-MI-A75C-01A	64 years, male, white, stage:i, alive, 291 days	0.0
TCGA-DD-A1EC-01A	20 years, female, white, stage:i, alive, 602 days	0.0
TCGA-EP-A26S-01A	70 years, male, white, stage:i, alive, 608 days	0.0
TCGA-G3-A25Z-01A	58 years, male, asian, stage:i, alive, 655 days	0.0
TCGA-G3-A25U-01A	63 years, female, asian, stage:i, alive, 1636 days	0.0
TCGA-2Y-A9GT-01A	51 years, male, white, stage:i, dead, 1624 days	0.0
TCGA-DD-A115-01A	53 years, male, white, stage:iiia, dead, 2542 days	0.0
TCGA-DD-A39Z-01A	43 years, female, stage:ii, dead, 601 days	0.0
TCGA-ZS-A9CD-01A	73 years, male, white, stage:ii, dead, 1386 days	0.0
TCGA-FV-A495-01A	51 years, female, white, stage:ii, alive, 1 days	0.0
TCGA-DD-A4NK-01A	80 years, female, white, stage:iiia, dead, 1210 days	0.0
TCGA-DD-AAD0-01A	73 years, female, asian, stage:i, alive, 137 days	0.0
TCGA-CC-5262-01A	67 years, male, asian, stage:iiic, dead, 103 days	0.0
TCGA-DD-AAE7-01A	72 years, male, asian, stage:i, alive, 644 days	0.0
TCGA-DD-AACZ-01A	63 years, female, asian, stage:i, dead, 171 days	0.0
TCGA-DD-A1EJ-01A	71 years, female, white, stage:iiic, dead, 1005 days	0.0
TCGA-DD-A4NP-01A	32 years, male, white, stage:i, alive, 3308 days	0.0
TCGA-EP-A12J-01A	63 years, male, black or african american, stage:i, alive, 570 days	0.0
TCGA-DD-AADA-01A	66 years, female, asian, stage:i, alive, 1233 days	0.0
TCGA-DD-A3A7-01A	67 years, male, stage:iiib, dead, 419 days	0.0
TCGA-K7-A6G5-01A	66 years, male, white, stage:i, alive, 512 days	0.0
TCGA-CC-A7II-01A	55 years, male, asian, stage:iiia, alive, 399 days	0.0
TCGA-G3-A25X-01A	73 years, male, asian, stage:ii, alive, 1779 days	0.0
TCGA-DD-AACA-01A	65 years, male, asian, stage:i, alive, 2301 days	0.0
TCGA-DD-AADR-01A	58 years, male, asian, stage:i, alive, 2028 days	0.0
TCGA-DD-AADI-01A	43 years, female, asian, stage:i, alive, 1085 days	0.0
TCGA-G3-A3CG-01A	80 years, male, white, stage:i, alive, 673 days	0.0
TCGA-RC-A6M4-01A	74 years, female, white, stage:iiia, alive, 22 days	0.0
TCGA-KR-A7K8-01A	57 years, male, stage:i, alive, 906 days	0.0
TCGA-DD-A39Y-01A	67 years, male, asian, stage:i, dead, 171 days	0.0
TCGA-G3-AAV0-01A	58 years, male, asian, stage:i, alive, 476 days	0.0
TCGA-WX-AA46-01A	62 years, male, white, stage:ii, alive, 756 days	0.0
TCGA-CC-A123-01A	24 years, female, asian, stage:iiia, alive, 219 days	0.0
TCGA-CC-A7IL-01A	61 years, male, asian, stage:iiia, dead, 278 days	0.0
TCGA-DD-A3A5-01A	66 years, female, white, stage:iii, dead, 3125 days	0.0
TCGA-G3-A25S-01A	64 years, male, white, stage:i, dead, 416 days	0.0
TCGA-ZS-A9CG-01A	55 years, male, white, stage:ii, alive, 341 days	0.0
TCGA-BC-A69I-01A	69 years, male, white, stage:i, alive, 387 days	0.0
TCGA-DD-AACF-01A	68 years, male, asian, stage:i, dead, 365 days	0.0
TCGA-KR-A7K7-01A	61 years, female, white, stage:ii, alive, 951 days	0.0
TCGA-DD-AAD6-01A	66 years, male, asian, stage:iiia, alive, 672 days	0.0
TCGA-DD-AAD3-01A	43 years, male, asian, stage:i, alive, 1295 days	0.0
TCGA-G3-A3CJ-01A	52 years, male, american indian or alaska native, stage:ii, alive, 594 days	0.0

TCGA-DD-AADN-01A	59 years, male, asian, stage:i, alive, 898 days	0.0
TCGA-XR-A8TC-01A	43 years, female, white, stage:i, alive, 1339 days	0.0
TCGA-DD-AAW1-01A	55 years, male, asian, stage:iiia, alive, 1989 days	0.0
TCGA-RC-A7S9-01A	47 years, female, asian, stage:i, alive, 640 days	0.0
TCGA-DD-AADY-01A	55 years, female, asian, stage:i, alive, 555 days	0.0
TCGA-2Y-A9H9-01A	70 years, male, white, stage:i, alive, 697 days	0.0
TCGA-WQ-AB4B-01A	62 years, male, white, stage:ii, alive, 395 days	0.0
TCGA-DD-AAW3-01A	69 years, male, asian, stage:i, alive, 1633 days	0.0
TCGA-DD-AAE1-01A	52 years, male, asian, stage:i, alive, 552 days	0.0
TCGA-2Y-A9H1-01A	58 years, male, white, stage:i, dead, 1229 days	0.0
TCGA-DD-AADD-01A	51 years, male, asian, stage:i, alive, 1231 days	0.0
TCGA-DD-AACD-01A	48 years, male, asian, stage:i, dead, 381 days	0.0
TCGA-DD-AAE6-01A	59 years, female, asian, stage:i, alive, 141 days	0.0
TCGA-DD-AADO-01A	55 years, male, asian, stage:i, alive, 453 days	0.0
TCGA-DD-A3A4-01A	37 years, male, white, stage:iiia, dead, 612 days	0.0
TCGA-CC-A7IG-01A	47 years, male, asian, stage:ii, dead, 299 days	0.0
TCGA-DD-A1EB-01A	72 years, female, stage:i, alive, 2017 days	0.0
TCGA-LG-A6GG-01A	79 years, female, white, stage:ii, alive, 387 days	0.0
TCGA-DD-A73B-01A	72 years, female, white, stage:i, dead, 283 days	0.0
TCGA-DD-AACH-01A	69 years, male, asian, stage:ii, dead, 195 days	0.0
TCGA-BD-A2L6-01A	69 years, male, white, alive, 1363 days	0.0
TCGA-DD-A11C-01A	69 years, male, white, stage:i, alive, 662 days	0.0
TCGA-2Y-A9H7-01A	81 years, female, white, stage:i, alive, 1168 days	0.0
TCGA-DD-AADQ-01A	59 years, male, asian, stage:ii, alive, 436 days	0.0
TCGA-MI-A75G-01A	63 years, male, white, stage:ii, alive, 698 days	0.0
TCGA-G3-AAV5-01A	67 years, male, white, stage:ii, alive, 354 days	0.0
TCGA-G3-A5SI-01A	44 years, male, asian, stage:ii, dead, 768 days	0.0
TCGA-RC-A7SF-01A	66 years, male, asian, stage:i, alive, 579 days	0.0
TCGA-DD-AADS-01A	63 years, male, asian, stage:i, alive, 474 days	0.0
TCGA-DD-AAVX-01A	38 years, male, asian, stage:ii, alive, 1570 days	0.0
TCGA-DD-AACS-01A	39 years, male, asian, stage:i, alive, 1804 days	0.0
TCGA-FV-A3R2-01A	75 years, male, white, stage:i, dead, 194 days	0.0
TCGA-ZP-A9D4-01A	64 years, female, white, alive, 395 days	0.0
TCGA-DD-AAE3-01A	50 years, male, asian, stage:i, alive, 566 days	0.0

TRPV4 RNA expression (N=365)

Sample	Description	FPKM
TCGA-CC-A3MA-01A	61 years, male, asian, stage:iiia, dead, 303 days	32.7
TCGA-DD-AADW-01A	48 years, male, asian, stage:i, alive, 587 days	25.8
TCGA-DD-A1EH-01A	23 years, male, white, stage:iii, alive, 1495 days	17.8
TCGA-G3-A25Y-01A	52 years, female, asian, stage:i, dead, 452 days	16.7
TCGA-DD-AA3A-01A	81 years, female, white, stage:i, dead, 410 days	15.7

TCGA-ED-A7PX-01A	48 years, female, asian, stage:ii, alive, 6 days	14.4
TCGA-DD-A4NH-01A	65 years, female, white, stage:iiib, alive, 917 days	12.5
TCGA-DD-AAE0-01A	45 years, female, asian, stage:iiia, alive, 555 days	12.0
TCGA-CC-A8HS-01A	18 years, male, asian, stage:iiic, dead, 300 days	12.0
TCGA-BC-A5W4-01A	69 years, male, white, stage:iiia, dead, 547 days	11.7
TCGA-T1-A6J8-01A	68 years, male, white, alive, 23 days	10.7
TCGA-DD-AACN-01A	32 years, male, asian, stage:i, alive, 1302 days	10.7
TCGA-2Y-A9H6-01A	68 years, female, white, stage:i, alive, 357 days	10.5
TCGA-5C-AAPD-01A	61 years, male, asian, stage:ii, alive, 20 days	10.4
TCGA-DD-A4NN-01A	56 years, female, white, stage:i, dead, 899 days	9.0
TCGA-ZP-A9D2-01A	51 years, male, white, dead, 765 days	8.4
TCGA-DD-AADY-01A	55 years, female, asian, stage:i, alive, 555 days	8.2
TCGA-DD-AAD1-01A	51 years, female, asian, stage:i, alive, 564 days	8.1
TCGA-ED-A82E-01A	60 years, female, asian, stage:iiia, alive, 408 days	7.8
TCGA-DD-AADK-01A	68 years, female, asian, stage:ii, alive, 1049 days	7.4
TCGA-K7-A5RF-01A	64 years, male, white, stage:i, alive, 631 days	7.3
TCGA-CC-A8HV-01A	51 years, female, asian, stage:ii, dead, 279 days	7.2
TCGA-FV-A3I0-01A	76 years, female, white, stage:ii, alive, 848 days	7.2
TCGA-DD-AADJ-01A	70 years, female, asian, stage:i, alive, 1066 days	6.9
TCGA-ED-A5KG-01A	60 years, female, asian, stage:ii, alive, 854 days	6.7
TCGA-FV-A3R3-01A	38 years, female, white, stage:i, dead, 366 days	6.6
TCGA-BC-4072-01B	74 years, female, white, stage:iiia, dead, 1490 days	6.5
TCGA-2Y-A9H2-01A	64 years, female, white, stage:i, alive, 1731 days	6.5
TCGA-FV-A23B-01A	70 years, female, white, stage:ii, dead, 1852 days	6.4
TCGA-ED-A66Y-01A	51 years, female, asian, stage:iiia, dead, 296 days	6.4
TCGA-WQ-A9G7-01A	female, white, alive, 30 days	6.4
TCGA-CC-A7IJ-01A	56 years, male, asian, stage:ii, alive, 382 days	6.2
TCGA-ED-A66X-01A	35 years, male, asian, stage:iiia, alive, 406 days	6.2
TCGA-ES-A2HS-01A	80 years, male, white, stage:i, dead, 688 days	6.1
TCGA-DD-AAD3-01A	43 years, male, asian, stage:i, alive, 1295 days	6.1
TCGA-DD-AACC-01A	61 years, male, asian, stage:i, dead, 1685 days	5.9
TCGA-DD-AADB-01A	51 years, male, asian, stage:i, alive, 1242 days	5.8
TCGA-DD-A4NJ-01A	54 years, female, white, stage:ii, alive, 928 days	5.8
TCGA-ED-A627-01A	74 years, male, white, stage:i, alive, 423 days	5.6
TCGA-DD-AAVS-01A	56 years, male, asian, stage:i, alive, 1823 days	5.6
TCGA-ED-A97K-01A	54 years, male, asian, stage:iiia, alive, 6 days	5.6
TCGA-FV-A3R2-01A	75 years, male, white, stage:i, dead, 194 days	5.4
TCGA-DD-AAW0-01A	54 years, male, asian, stage:i, alive, 2015 days	5.3
TCGA-DD-A1EJ-01A	71 years, female, white, stage:iiic, dead, 1005 days	5.1
TCGA-ZS-A9CF-01A	64 years, male, white, stage:ii, alive, 2412 days	4.9
TCGA-DD-A11B-01A	73 years, male, white, stage:i, dead, 14 days	4.8
TCGA-ED-A8O5-01A	59 years, female, asian, stage:iiia, alive, 406 days	4.7
TCGA-FV-A4ZP-01A	78 years, male, white, stage:iiia, dead, 2486 days	4.6

TCGA-2Y-A9GV-01A	54 years, female, white, stage:i, dead, 2532 days	4.6
TCGA-DD-A4NE-01A	75 years, female, white, stage:iiia, dead, 660 days	4.2
TCGA-DD-A114-01A	42 years, male, black or african american, stage:ii, dead, 1149 days	4.2
TCGA-RC-A7SB-01A	53 years, male, asian, stage:ii, alive, 588 days	4.1
TCGA-DD-A1EF-01A	57 years, female, white, stage:i, dead, 394 days	4.1
TCGA-DD-A1EK-01A	64 years, female, white, stage:ivb, dead, 558 days	4.0
TCGA-2Y-A9GX-01A	68 years, male, white, stage:i, alive, 2442 days	3.8
TCGA-2Y-A9H7-01A	81 years, female, white, stage:i, alive, 1168 days	3.8
TCGA-UB-A7MA-01A	62 years, female, white, stage:ii, alive, 848 days	3.8
TCGA-UB-AA0V-01A	69 years, female, white, stage:i, alive, 314 days	3.7
TCGA-G3-A25V-01A	68 years, male, white, stage:i, alive, 860 days	3.7
TCGA-WX-AA44-01A	64 years, female, white, stage:i, alive, 615 days	3.6
TCGA-DD-A3A9-01A	64 years, female, white, stage:ivb, dead, 931 days	3.5
TCGA-ED-A459-01A	47 years, male, asian, stage:ii, alive, 910 days	3.4
TCGA-RC-A7SK-01A	59 years, male, asian, stage:i, alive, 472 days	3.4
TCGA-G3-AAUZ-01A	48 years, male, stage:i, alive, 480 days	3.3
TCGA-CC-A3M9-01A	45 years, male, asian, stage:iiia, dead, 300 days	3.3
TCGA-DD-AACS-01A	39 years, male, asian, stage:i, alive, 1804 days	3.3
TCGA-G3-A7M9-01A	70 years, male, white, stage:iiib, dead, 56 days	3.3
TCGA-ED-A7PY-01A	20 years, female, asian, stage:ii, alive, 390 days	3.3
TCGA-K7-AAU7-01A	61 years, male, white, stage:ii, alive, 359 days	3.3
TCGA-CC-A7II-01A	55 years, male, asian, stage:iiia, alive, 399 days	3.2
TCGA-DD-AADL-01A	58 years, male, asian, stage:i, alive, 636 days	3.2
TCGA-DD-A73G-01A	73 years, female, white, stage:i, alive, 3478 days	3.2
TCGA-G3-A25Z-01A	58 years, male, asian, stage:i, alive, 655 days	3.1
TCGA-DD-AAVQ-01A	38 years, male, asian, stage:i, alive, 2728 days	3.1
TCGA-DD-AAVW-01A	35 years, male, asian, stage:i, alive, 2317 days	3.1
TCGA-DD-AACW-01A	43 years, male, asian, stage:i, alive, 1424 days	3.0
TCGA-DD-A113-01A	55 years, female, white, stage:ii, alive, 2425 days	3.0
TCGA-BC-A10R-01A	66 years, female, white, dead, 308 days	2.9
TCGA-GJ-A3OU-01A	59 years, male, white, stage:i, alive, 879 days	2.9
TCGA-BC-A110-01A	51 years, female, black or african american, dead, 2116 days	2.9
TCGA-DD-AACL-01A	66 years, female, asian, stage:i, dead, 107 days	2.9
TCGA-DD-AADM-01A	58 years, male, asian, stage:ii, dead, 12 days	2.8
TCGA-BD-A3ER-01A	62 years, male, white, stage:ii, alive, 1115 days	2.8
TCGA-DD-AAVU-01A	46 years, male, asian, stage:ii, alive, 2202 days	2.7
TCGA-DD-A1EI-01A	46 years, male, asian, stage:i, alive, 183 days	2.5
TCGA-2Y-A9H5-01A	59 years, female, white, stage:i, dead, 555 days	2.5
TCGA-G3-A5SM-01A	58 years, male, white, stage:ii, alive, 520 days	2.5
TCGA-5R-AAAM-01A	65 years, female, white, stage:ii, dead, 46 days	2.4
TCGA-DD-A4NB-01A	25 years, male, white, stage:i, alive, 989 days	2.4
TCGA-ZS-A9CD-01A	73 years, male, white, stage:ii, dead, 1386 days	2.4
TCGA-DD-AAVZ-01A	38 years, male, asian, stage:i, alive, 1900 days	2.4

TCGA-CC-A8HU-01A	39 years, female, asian, stage:iiia, dead, 344 days	2.3
TCGA-DD-A39Z-01A	43 years, female, stage:ii, dead, 601 days	2.3
TCGA-G3-A3CJ-01A	52 years, male, american indian or alaska native, stage:ii, alive, 594 days	2.3
TCGA-DD-A4NS-01A	62 years, female, white, stage:i, dead, 2456 days	2.3
TCGA-BC-A10X-01A	52 years, female, white, stage:iiia, dead, 770 days	2.3
TCGA-DD-A4ND-01A	56 years, female, white, stage:i, alive, 2746 days	2.2
TCGA-DD-A4NV-01A	61 years, male, white, stage:iiia, alive, 2398 days	2.1
TCGA-DD-A73B-01A	72 years, female, white, stage:i, dead, 283 days	2.1
TCGA-ED-A4XI-01A	58 years, male, asian, stage:ii, alive, 819 days	2.1
TCGA-CC-A5UC-01A	63 years, male, asian, stage:iiia, dead, 347 days	2.0
TCGA-EP-A2KB-01A	46 years, female, white, stage:i, dead, 596 days	2.0
TCGA-DD-A116-01A	68 years, male, asian, stage:iiia, dead, 1622 days	2.0
TCGA-FV-A495-01A	51 years, female, white, stage:ii, alive, 1 days	2.0
TCGA-UB-AA0U-01A	60 years, male, white, stage:ii, alive, 327 days	1.9
TCGA-ZP-A9D1-01A	56 years, female, white, alive, 21 days	1.9
TCGA-DD-AAD2-01A	66 years, male, asian, stage:i, alive, 658 days	1.9
TCGA-XR-A8TG-01A	59 years, male, white, stage:i, alive, 898 days	1.9
TCGA-UB-A7MF-01A	57 years, male, white, stage:iiia, dead, 214 days	1.8
TCGA-CC-A7IE-01A	57 years, male, asian, stage:iiia, dead, 217 days	1.8
TCGA-DD-AACO-01A	40 years, male, asian, stage:i, alive, 1876 days	1.7
TCGA-DD-AADP-01A	45 years, male, asian, stage:i, alive, 458 days	1.7
TCGA-MI-A75E-01A	61 years, male, white, stage:iiic, alive, 507 days	1.7
TCGA-CC-A5UD-01A	45 years, male, asian, stage:iiia, dead, 304 days	1.6
TCGA-HP-A5MZ-01A	62 years, male, stage:i, dead, 91 days	1.6
TCGA-2Y-A9GT-01A	51 years, male, white, stage:i, dead, 1624 days	1.6
TCGA-DD-AAD5-01A	54 years, male, asian, stage:i, alive, 1345 days	1.6
TCGA-2Y-A9H3-01A	45 years, male, white, stage:ii, alive, 1516 days	1.6
TCGA-G3-A3CH-01A	53 years, male, asian, stage:iiia, alive, 780 days	1.5
TCGA-DD-AACP-01A	65 years, male, asian, stage:i, alive, 415 days	1.5
TCGA-DD-AADO-01A	55 years, male, asian, stage:i, alive, 453 days	1.5
TCGA-DD-AAEH-01A	73 years, male, asian, stage:i, alive, 784 days	1.4
TCGA-CC-5260-01A	61 years, female, asian, stage:iiic, dead, 87 days	1.4
TCGA-BC-A112-01A	80 years, male, white, dead, 153 days	1.4
TCGA-DD-AACT-01A	69 years, female, asian, stage:i, alive, 1562 days	1.4
TCGA-DD-A4NL-01A	46 years, male, white, stage:i, alive, 1711 days	1.4
TCGA-G3-A3CI-01A	71 years, male, white, stage:i, alive, 180 days	1.4
TCGA-ZP-A9CY-01A	66 years, female, white, alive, 782 days	1.4
TCGA-ED-A7XO-01A	29 years, male, asian, stage:iiia, alive, 427 days	1.4
TCGA-G3-AAV6-01A	53 years, female, white, stage:iiia, dead, 65 days	1.3
TCGA-DD-AAVR-01A	44 years, male, asian, stage:i, alive, 2513 days	1.3
TCGA-DD-A4NK-01A	80 years, female, white, stage:iiia, dead, 1210 days	1.3
TCGA-DD-A1EG-01A	77 years, male, white, stage:i, dead, 1372 days	1.3
TCGA-FV-A2QQ-01A	80 years, male, white, stage:i, alive, 729 days	1.3

TCGA-DD-A39Y-01A	67 years, male, asian, stage:i, dead, 171 days	1.3
TCGA-FV-A3I1-01A	female, white, stage:ii, dead, 247 days	1.3
TCGA-2Y-A9GY-01A	64 years, female, white, stage:ii, dead, 757 days	1.3
TCGA-YA-A8S7-01A	69 years, male, white, stage:iiia, dead, 412 days	1.3
TCGA-WX-AA46-01A	62 years, male, white, stage:ii, alive, 756 days	1.3
TCGA-ZP-A9CZ-01A	72 years, male, asian, alive, 706 days	1.2
TCGA-CC-A7IF-01A	59 years, male, asian, stage:iiia, dead, 649 days	1.2
TCGA-GJ-A9DB-01A	68 years, male, white, stage:i, dead, 67 days	1.2
TCGA-DD-AACZ-01A	63 years, female, asian, stage:i, dead, 171 days	1.2
TCGA-RC-A7S9-01A	47 years, female, asian, stage:i, alive, 640 days	1.2
TCGA-CC-A1HT-01A	50 years, male, asian, stage:iiia, dead, 101 days	1.2
TCGA-DD-A4NR-01A	85 years, female, white, stage:i, dead, 9 days	1.2
TCGA-DD-A119-01A	40 years, male, asian, stage:iv, dead, 223 days	1.1
TCGA-BC-A10S-01A	81 years, male, white, dead, 1423 days	1.1
TCGA-CC-A3MB-01A	36 years, male, asian, stage:iiia, dead, 315 days	1.1
TCGA-ED-A8O6-01A	50 years, female, asian, stage:iiia, dead, 56 days	1.0
TCGA-G3-A25X-01A	73 years, male, asian, stage:ii, alive, 1779 days	1.0
TCGA-CC-5261-01A	44 years, male, asian, stage:ii, dead, 97 days	1.0
TCGA-G3-A7M6-01A	60 years, female, white, stage:i, alive, 632 days	1.0
TCGA-DD-AADR-01A	58 years, male, asian, stage:i, alive, 2028 days	1.0
TCGA-DD-A3A1-01A	65 years, male, stage:iiia, dead, 233 days	1.0
TCGA-ZP-A9D0-01A	67 years, female, black or african american, alive, 1091 days	1.0
TCGA-5C-A9VG-01A	58 years, male, white, stage:ii, alive, 328 days	0.9
TCGA-CC-A3MC-01A	54 years, male, asian, stage:iiia, alive, 363 days	0.9
TCGA-CC-A123-01A	24 years, female, asian, stage:iiia, alive, 219 days	0.9
TCGA-XR-A8TC-01A	43 years, female, white, stage:i, alive, 1339 days	0.9
TCGA-FV-A4ZQ-01A	52 years, male, white, stage:i, alive, 12 days	0.9
TCGA-K7-A6G5-01A	66 years, male, white, stage:i, alive, 512 days	0.9
TCGA-2Y-A9H4-01A	68 years, male, black or african american, stage:i, alive, 1452 days	0.9
TCGA-GJ-A6C0-01A	75 years, female, white, stage:ii, dead, 31 days	0.9
TCGA-G3-A7M5-01A	76 years, male, asian, stage:i, alive, 447 days	0.8
TCGA-MR-A520-01A	58 years, male, white, stage:i, alive, 229 days	0.8
TCGA-XR-A8TD-01A	49 years, female, white, stage:iiib, alive, 1030 days	0.8
TCGA-CC-A5UE-01A	48 years, male, asian, stage:iiib, dead, 272 days	0.8
TCGA-DD-A1ED-01A	68 years, male, white, stage:i, alive, 2301 days	0.8
TCGA-CC-A9FS-01A	55 years, male, asian, stage:ii, alive, 211 days	0.8
TCGA-DD-AAEG-01A	59 years, female, asian, stage:i, alive, 719 days	0.8
TCGA-LG-A9QD-01A	68 years, male, white, stage:iiia, alive, 366 days	0.8
TCGA-DD-AACG-01A	52 years, male, asian, stage:ii, dead, 469 days	0.8
TCGA-EP-A3RK-01A	73 years, male, white, stage:iiia, alive, 363 days	0.8
TCGA-CC-5258-01A	48 years, male, asian, stage:ii, dead, 129 days	0.7
TCGA-BC-A69I-01A	69 years, male, white, stage:i, alive, 387 days	0.7
TCGA-G3-A25T-01A	45 years, female, white, stage:iiia, alive, 1553 days	0.7

TCGA-G3-AAV0-01A	58 years, male, asian, stage:i, alive, 476 days	0.7
TCGA-2Y-A9GU-01A	55 years, female, white, stage:i, alive, 1939 days	0.7
TCGA-CC-A8HT-01A	74 years, male, asian, stage:iiia, dead, 140 days	0.7
TCGA-5R-AA1D-01A	17 years, female, white, stage:iiia, alive, 449 days	0.6
TCGA-DD-AAC8-01A	72 years, male, asian, stage:i, dead, 16 days	0.6
TCGA-LG-A6GG-01A	79 years, female, white, stage:ii, alive, 387 days	0.6
TCGA-DD-AACH-01A	69 years, male, asian, stage:ii, dead, 195 days	0.6
TCGA-BC-4073-01B	73 years, male, white, stage:iiia, alive, 849 days	0.6
TCGA-2Y-A9GS-01A	58 years, male, white, dead, 724 days	0.6
TCGA-BC-A10W-01A	50 years, male, asian, dead, 91 days	0.6
TCGA-DD-A1EC-01A	20 years, female, white, stage:i, alive, 602 days	0.6
TCGA-DD-A4NO-01A	66 years, male, white, stage:i, alive, 2245 days	0.6
TCGA-CC-5259-01A	60 years, female, asian, stage:iiic, alive, 250 days	0.6
TCGA-CC-5263-01A	35 years, male, asian, stage:iiia, dead, 129 days	0.6
TCGA-G3-A5SK-01A	58 years, male, white, stage:i, alive, 744 days	0.6
TCGA-DD-AACF-01A	68 years, male, asian, stage:i, dead, 365 days	0.6
TCGA-DD-A4NA-01A	67 years, female, white, stage:iiic, alive, 1008 days	0.6
TCGA-K7-A5RG-01A	66 years, male, black or african american, stage:i, alive, 519 days	0.5
TCGA-5C-A9VH-01A	70 years, male, white, stage:i, alive, 322 days	0.5
TCGA-KR-A7K8-01A	57 years, male, stage:i, alive, 906 days	0.5
TCGA-DD-A4NG-01A	77 years, male, white, stage:iiia, dead, 802 days	0.5
TCGA-ED-A7PZ-01A	61 years, male, asian, stage:ii, alive, 6 days	0.5
TCGA-DD-AAVV-01A	56 years, male, asian, stage:ii, alive, 2455 days	0.5
TCGA-DD-A118-01A	77 years, female, white, stage:ii, alive, 3437 days	0.5
TCGA-CC-5262-01A	67 years, male, asian, stage:iiic, dead, 103 days	0.5
TCGA-RC-A6M5-01A	20 years, female, white, stage:iva, alive, 15 days	0.5
TCGA-2Y-A9GW-01A	64 years, male, white, stage:i, dead, 1271 days	0.5
TCGA-DD-AAEK-01A	51 years, male, asian, stage:ii, alive, 1067 days	0.5
TCGA-DD-AADQ-01A	59 years, male, asian, stage:ii, alive, 436 days	0.5
TCGA-BC-A8YO-01A	66 years, female, white, stage:iiic, alive, 562 days	0.5
TCGA-MI-A75H-01A	77 years, male, white, alive, 747 days	0.5
TCGA-G3-AAV2-01A	50 years, male, white, stage:i, alive, 372 days	0.5
TCGA-G3-AAV7-01A	38 years, male, asian, stage:ii, alive, 361 days	0.5
TCGA-DD-A73F-01A	77 years, female, white, stage:i, alive, 1085 days	0.4
TCGA-UB-A7MD-01A	67 years, male, black or african american, stage:i, dead, 52 days	0.4
TCGA-DD-A73A-01A	71 years, male, white, stage:i, alive, 728 days	0.4
TCGA-DD-A3A8-01A	75 years, male, white, stage:ii, dead, 11 days	0.4
TCGA-BC-A10Q-01A	72 years, female, white, dead, 1135 days	0.4
TCGA-DD-A4NP-01A	32 years, male, white, stage:i, alive, 3308 days	0.4
TCGA-G3-A5SI-01A	44 years, male, asian, stage:ii, dead, 768 days	0.4
TCGA-DD-AACA-01A	65 years, male, asian, stage:i, alive, 2301 days	0.4
TCGA-DD-AAE7-01A	72 years, male, asian, stage:i, alive, 644 days	0.4
TCGA-DD-AACU-01A	59 years, male, asian, stage:i, alive, 1567 days	0.4

TCGA-DD-AAVX-01A	38 years, male, asian, stage:ii, alive, 1570 days	0.4
TCGA-DD-A4NI-01A	67 years, male, white, stage:ii, alive, 816 days	0.4
TCGA-DD-AACB-01A	74 years, female, asian, stage:i, alive, 2324 days	0.4
TCGA-UB-A7ME-01A	51 years, male, asian, stage:i, alive, 486 days	0.4
TCGA-EP-A3JL-01A	76 years, male, white, stage:i, alive, 303 days	0.4
TCGA-MR-A8JO-01A	34 years, male, white, stage:i, alive, 330 days	0.4
TCGA-DD-A39V-01A	77 years, male, white, stage:ii, dead, 643 days	0.4
TCGA-4R-AA8I-01A	66 years, male, white, stage:ii, dead, 262 days	0.4
TCGA-DD-A1EA-01A	68 years, male, asian, stage:ii, alive, 2415 days	0.4
TCGA-BC-A69H-01A	64 years, male, white, stage:ii, alive, 444 days	0.4
TCGA-G3-A5SJ-01A	59 years, male, white, stage:i, alive, 698 days	0.4
TCGA-DD-A1EE-01A	73 years, male, white, stage:iiia, dead, 349 days	0.3
TCGA-G3-A7M8-01A	31 years, male, asian, stage:i, alive, 430 days	0.3
TCGA-PD-A5DF-01A	58 years, female, white, stage:iiib, dead, 639 days	0.3
TCGA-RC-A6M4-01A	74 years, female, white, stage:iiia, alive, 22 days	0.3
TCGA-DD-AADD-01A	51 years, male, asian, stage:i, alive, 1231 days	0.3
TCGA-XR-A8TF-01A	74 years, male, white, stage:i, dead, 693 days	0.3
TCGA-DD-A3A6-01A	72 years, female, white, stage:ii, dead, 3258 days	0.3
TCGA-BC-A3KF-01A	66 years, female, white, stage:i, alive, 8 days	0.3
TCGA-BD-A2L6-01A	69 years, male, white, alive, 1363 days	0.3
TCGA-DD-A4NQ-01A	60 years, male, white, stage:ii, dead, 373 days	0.3
TCGA-G3-A25S-01A	64 years, male, white, stage:i, dead, 416 days	0.3
TCGA-DD-A115-01A	53 years, male, white, stage:iiia, dead, 2542 days	0.3
TCGA-MI-A75I-01A	61 years, male, black or african american, alive, 630 days	0.3
TCGA-G3-AAV3-01A	58 years, female, white, stage:ii, alive, 412 days	0.3
TCGA-DD-AACX-01A	66 years, male, asian, stage:ii, alive, 170 days	0.3
TCGA-DD-AADN-01A	59 years, male, asian, stage:i, alive, 898 days	0.3
TCGA-O8-A75V-01A	54 years, male, stage:i, alive, 538 days	0.3
TCGA-DD-AADI-01A	43 years, female, asian, stage:i, alive, 1085 days	0.3
TCGA-EP-A12J-01A	63 years, male, black or african american, stage:i, alive, 570 days	0.3
TCGA-DD-A3A2-01A	76 years, female, white, stage:i, dead, 2131 days	0.3
TCGA-DD-AACI-01A	69 years, male, asian, stage:ii, alive, 1618 days	0.3
TCGA-BD-A3EP-01A	76 years, female, black or african american, stage:i, alive, 409 days	0.3
TCGA-BC-A217-01A	75 years, female, white, stage:ii, dead, 1397 days	0.3
TCGA-BC-A10T-01A	76 years, male, white, dead, 837 days	0.3
TCGA-DD-AACY-01A	61 years, male, asian, stage:i, alive, 1450 days	0.3
TCGA-DD-AAW3-01A	69 years, male, asian, stage:i, alive, 1633 days	0.3
TCGA-EP-A26S-01A	70 years, male, white, stage:i, alive, 608 days	0.3
TCGA-KR-A7K2-01A	64 years, male, white, stage:i, alive, 829 days	0.3
TCGA-ZP-A9CV-01A	59 years, male, white, dead, 1088 days	0.3
TCGA-DD-AADC-01A	53 years, male, asian, stage:i, dead, 425 days	0.3
TCGA-G3-A3CK-01A	61 years, male, asian, stage:i, alive, 585 days	0.2
TCGA-DD-AACE-01A	62 years, male, asian, stage:i, alive, 2184 days	0.2

TCGA-HP-A5N0-01A	88 years, female, dead, 1147 days	0.2
TCGA-KR-A7K0-01A	65 years, male, white, stage:i, dead, 65 days	0.2
TCGA-DD-AADA-01A	66 years, female, asian, stage:i, alive, 1233 days	0.2
TCGA-DD-AAC9-01A	51 years, male, asian, stage:i, alive, 347 days	0.2
TCGA-BC-A10Y-01A	76 years, male, white, dead, 711 days	0.2
TCGA-DD-A39X-01A	78 years, female, white, stage:i, dead, 1694 days	0.2
TCGA-DD-AAVP-01A	48 years, male, asian, stage:i, alive, 2752 days	0.2
TCGA-DD-AACD-01A	48 years, male, asian, stage:i, dead, 381 days	0.2
TCGA-DD-AADV-01A	50 years, male, asian, stage:i, alive, 574 days	0.2
TCGA-DD-AAE4-01A	49 years, female, asian, stage:i, alive, 608 days	0.2
TCGA-KR-A7K7-01A	61 years, female, white, stage:ii, alive, 951 days	0.2
TCGA-ZP-A9D4-01A	64 years, female, white, alive, 395 days	0.2
TCGA-2Y-A9HA-01A	70 years, male, white, stage:ii, dead, 36 days	0.2
TCGA-DD-AAD6-01A	66 years, male, asian, stage:iiia, alive, 672 days	0.2
TCGA-DD-AAE3-01A	50 years, male, asian, stage:i, alive, 566 days	0.2
TCGA-DD-AAE2-01A	51 years, male, asian, stage:i, alive, 638 days	0.2
TCGA-DD-A3A3-01A	45 years, male, asian, stage:i, dead, 535 days	0.2
TCGA-RC-A6M6-01A	75 years, male, white, stage:ii, alive, 9 days	0.2
TCGA-DD-AACK-01A	70 years, male, asian, stage:i, alive, 9 days	0.2
TCGA-G3-AAV1-01A	51 years, male, asian, stage:iiic, dead, 359 days	0.2
TCGA-FV-A2QR-01A	male, white, stage:i, dead, 581 days	0.2
TCGA-WQ-AB4B-01A	62 years, male, white, stage:ii, alive, 395 days	0.2
TCGA-DD-A1EL-01A	23 years, male, black or african american, stage:ii, dead, 415 days	0.2
TCGA-DD-AACQ-01A	50 years, male, asian, stage:ii, dead, 432 days	0.2
TCGA-DD-A4NF-01A	72 years, male, white, stage:i, alive, 942 days	0.2
TCGA-DD-AADF-01A	64 years, female, asian, stage:i, dead, 115 days	0.2
TCGA-ED-A7XP-01A	53 years, female, asian, stage:ii, alive, 400 days	0.2
TCGA-2Y-A9HB-01A	66 years, male, stage:i, alive, 260 days	0.2
TCGA-RG-A7D4-01A	69 years, male, black or african american, stage:ii, alive, 1098 days	0.2
TCGA-2Y-A9H0-01A	49 years, male, white, stage:iiia, alive, 3675 days	0.2
TCGA-CC-A7IG-01A	47 years, male, asian, stage:ii, dead, 299 days	0.2
TCGA-2Y-A9H9-01A	70 years, male, white, stage:i, alive, 697 days	0.2
TCGA-DD-AAW1-01A	55 years, male, asian, stage:iiia, alive, 1989 days	0.2
TCGA-G3-AAV5-01A	67 years, male, white, stage:ii, alive, 354 days	0.2
TCGA-CC-A9FW-01A	68 years, male, asian, stage:iiia, alive, 248 days	0.2
TCGA-EP-A2KA-01A	52 years, female, white, stage:iiia, dead, 627 days	0.2
TCGA-ES-A2HT-01A	54 years, male, black or african american, stage:i, dead, 438 days	0.2
TCGA-BW-A5NO-01A	50 years, male, black or african american, stage:iiia, alive, 20 days	0.1
TCGA-RC-A7SF-01A	66 years, male, asian, stage:i, alive, 579 days	0.1
TCGA-DD-A73C-01A	65 years, female, white, stage:iiia, alive, 701 days	0.1
TCGA-2Y-A9H8-01A	85 years, female, white, dead, 633 days	0.1
TCGA-BC-A216-01A	62 years, female, white, stage:iiia, alive, 1351 days	0.1
TCGA-5R-AA1C-01A	57 years, male, white, stage:ii, alive, 520 days	0.1

TCGA-G3-A6UC-01A	65 years, male, white, stage:iiib, alive, 671 days	0.1
TCGA-G3-AAV4-01A	83 years, female, white, stage:i, dead, 27 days	0.1
TCGA-ZS-A9CE-01A	79 years, female, white, stage:ii, alive, 1241 days	0.1
TCGA-DD-A11C-01A	69 years, male, white, stage:i, alive, 662 days	0.1
TCGA-DD-AAEI-01A	72 years, male, asian, stage:i, alive, 1531 days	0.1
TCGA-DD-AACJ-01A	75 years, male, asian, stage:ii, alive, 2102 days	0.1
TCGA-FV-A496-01A	84 years, female, white, stage:i, alive, 10 days	0.1
TCGA-BC-A10U-01A	69 years, male, white, dead, 837 days	0.1
TCGA-DD-AAD8-01A	73 years, female, asian, stage:i, alive, 1219 days	0.1
TCGA-DD-AACV-01A	53 years, male, asian, stage:i, alive, 1531 days	0.1
TCGA-WJ-A86L-01A	68 years, female, white, stage:i, alive, 345 days	0.1
TCGA-NI-A8LF-01A	74 years, male, white, stage:i, alive, 799 days	0.1
TCGA-DD-A1EB-01A	72 years, female, stage:i, alive, 2017 days	0.1
TCGA-EP-A2KC-01A	62 years, male, black or african american, stage:i, dead, 19 days	0.1
TCGA-2Y-A9GZ-01A	82 years, female, white, stage:ii, dead, 848 days	0.1
TCGA-MI-A75G-01A	63 years, male, white, stage:ii, alive, 698 days	0.1
TCGA-NI-A4U2-01A	71 years, male, white, stage:iiia, dead, 1791 days	0.1
TCGA-DD-AAE9-01A	69 years, male, asian, stage:i, alive, 722 days	0.1
TCGA-DD-AAE1-01A	52 years, male, asian, stage:i, alive, 552 days	0.1
TCGA-DD-AAEA-01A	65 years, male, asian, stage:i, alive, 575 days	0.1
TCGA-MI-A75C-01A	64 years, male, white, stage:i, alive, 291 days	0.1
TCGA-CC-A7IH-01A	58 years, male, asian, stage:iiia, alive, 365 days	0.1
TCGA-LG-A9QC-01A	48 years, male, white, stage:i, alive, 425 days	0.1
TCGA-3K-AAZ8-01A	65 years, male, black or african american, stage:iiib, alive, 396 days	0.1
TCGA-BC-A3KG-01A	68 years, female, white, stage:ii, alive, 680 days	0.1
TCGA-UB-A7MC-01A	59 years, male, white, stage:iiia, alive, 500 days	0.1
TCGA-QA-A7B7-01A	48 years, male, black or african american, stage:ii, alive, 94 days	0.1
TCGA-DD-AAVY-01A	56 years, male, asian, stage:iiia, alive, 1970 days	0.1
TCGA-G3-A3CG-01A	80 years, male, white, stage:i, alive, 673 days	0.1
TCGA-DD-AADS-01A	63 years, male, asian, stage:i, alive, 474 days	0.1
TCGA-G3-A5SL-01A	70 years, male, white, stage:ii, alive, 621 days	0.1
TCGA-CC-5264-01A	71 years, male, asian, stage:iiia, dead, 102 days	0.1
TCGA-XR-A8TE-01A	16 years, male, white, stage:iiia, alive, 925 days	0.1
TCGA-DD-AAEE-01A	55 years, male, asian, stage:i, alive, 810 days	0.1
TCGA-G3-A7M7-01A	65 years, male, white, stage:i, alive, 361 days	0.1
TCGA-DD-A39W-01A	29 years, female, white, stage:iii, dead, 827 days	0.1
TCGA-RC-A7SH-01A	42 years, male, asian, stage:ii, alive, 468 days	0.1
TCGA-DD-AAED-01A	51 years, male, asian, stage:i, alive, 763 days	0.1
TCGA-UB-A7MB-01A	24 years, male, white, stage:ii, alive, 601 days	0.1
TCGA-DD-A11D-01A	57 years, female, white, stage:i, dead, 1560 days	0.1
TCGA-ZS-A9CG-01A	55 years, male, white, stage:ii, alive, 341 days	0.1
TCGA-DD-AAW2-01A	69 years, male, asian, stage:i, alive, 1855 days	0.1
TCGA-2Y-A9H1-01A	58 years, male, white, stage:i, dead, 1229 days	0.1

TCGA-CC-A7IK-01A	59 years, male, asian, stage:iiia, dead, 262 days	0.1
TCGA-G3-A25U-01A	63 years, female, asian, stage:i, alive, 1636 days	0.1
TCGA-DD-A3A7-01A	67 years, male, stage:iiib, dead, 419 days	0.1
TCGA-DD-A73E-01A	66 years, male, white, stage:i, alive, 44 days	0.0
TCGA-DD-A3A5-01A	66 years, female, white, stage:iii, dead, 3125 days	0.0
TCGA-DD-AADG-01A	70 years, male, asian, stage:iiia, alive, 1145 days	0.0
TCGA-WX-AA47-01A	33 years, female, white, stage:iiia, dead, 556 days	0.0
TCGA-DD-AADU-01A	60 years, male, asian, stage:ii, alive, 554 days	0.0
TCGA-DD-AAD0-01A	73 years, female, asian, stage:i, alive, 137 days	0.0
TCGA-DD-A3A4-01A	37 years, male, white, stage:iiia, dead, 612 days	0.0
TCGA-DD-A11A-01A	67 years, male, black or african american, stage:i, alive, 79 days	0.0
TCGA-DD-AAE6-01A	59 years, female, asian, stage:i, alive, 141 days	0.0
TCGA-BC-A10Z-01A	62 years, female, white, stage:i, dead, 34 days	0.0
TCGA-DD-AAEB-01A	60 years, male, asian, stage:i, alive, 478 days	0.0
TCGA-DD-A73D-01A	68 years, female, white, stage:ii, alive, 693 days	0.0
TCGA-CC-A7IL-01A	61 years, male, asian, stage:iiia, dead, 278 days	0.0

TRPV5 RNA expression (N=365)

Sample	Description	FPKM
TCGA-G3-A25X-01A	73 years, male, asian, stage:ii, alive, 1779 days	0.4
TCGA-G3-A3CI-01A	71 years, male, white, stage:i, alive, 180 days	0.3
TCGA-G3-AAV7-01A	38 years, male, asian, stage:ii, alive, 361 days	0.2
TCGA-CC-5263-01A	35 years, male, asian, stage:iiia, dead, 129 days	0.2
TCGA-BC-A112-01A	80 years, male, white, dead, 153 days	0.2
TCGA-DD-A114-01A	42 years, male, black or african american, stage:ii, dead, 1149 days	0.2
TCGA-DD-AADY-01A	55 years, female, asian, stage:i, alive, 555 days	0.1
TCGA-BC-4073-01B	73 years, male, white, stage:iiia, alive, 849 days	0.1
TCGA-BC-A5W4-01A	69 years, male, white, stage:iiia, dead, 547 days	0.1
TCGA-CC-A3MC-01A	54 years, male, asian, stage:iiia, alive, 363 days	0.1
TCGA-CC-5260-01A	61 years, female, asian, stage:iiic, dead, 87 days	0.1
TCGA-DD-AAVV-01A	56 years, male, asian, stage:ii, alive, 2455 days	0.1
TCGA-BC-A10W-01A	50 years, male, asian, dead, 91 days	0.1
TCGA-BC-A8YO-01A	66 years, female, white, stage:iiic, alive, 562 days	0.1
TCGA-ED-A7PX-01A	48 years, female, asian, stage:ii, alive, 6 days	0.1
TCGA-G3-A5SJ-01A	59 years, male, white, stage:i, alive, 698 days	0.1
TCGA-G3-A25T-01A	45 years, female, white, stage:iiia, alive, 1553 days	0.1
TCGA-EP-A2KA-01A	52 years, female, white, stage:iiia, dead, 627 days	0.1
TCGA-DD-AACI-01A	69 years, male, asian, stage:ii, alive, 1618 days	0.1
TCGA-CC-A3MA-01A	61 years, male, asian, stage:iiia, dead, 303 days	0.1
TCGA-CC-A1HT-01A	50 years, male, asian, stage:iiia, dead, 101 days	0.1
TCGA-DD-AAEG-01A	59 years, female, asian, stage:i, alive, 719 days	0.1
TCGA-CC-A5UE-01A	48 years, male, asian, stage:iiib, dead, 272 days	0.1

TCGA-DD-AADK-01A	68 years, female, asian, stage:ii, alive, 1049 days	0.1
TCGA-BC-A10Q-01A	72 years, female, white, dead, 1135 days	0.0
TCGA-UB-A7MA-01A	62 years, female, white, stage:ii, alive, 848 days	0.0
TCGA-ED-A97K-01A	54 years, male, asian, stage:iiia, alive, 6 days	0.0
TCGA-ED-A82E-01A	60 years, female, asian, stage:iiia, alive, 408 days	0.0
TCGA-DD-AACH-01A	69 years, male, asian, stage:ii, dead, 195 days	0.0
TCGA-G3-A7M9-01A	70 years, male, white, stage:iiib, dead, 56 days	0.0
TCGA-GJ-A3OU-01A	59 years, male, white, stage:i, alive, 879 days	0.0
TCGA-2Y-A9H2-01A	64 years, female, white, stage:i, alive, 1731 days	0.0
TCGA-BC-4072-01B	74 years, female, white, stage:iiia, dead, 1490 days	0.0
TCGA-DD-A4NA-01A	67 years, female, white, stage:iiic, alive, 1008 days	0.0
TCGA-CC-A3MB-01A	36 years, male, asian, stage:iiia, dead, 315 days	0.0
TCGA-K7-A5RG-01A	66 years, male, black or african american, stage:i, alive, 519 days	0.0
TCGA-ED-A459-01A	47 years, male, asian, stage:ii, alive, 910 days	0.0
TCGA-5C-AAPD-01A	61 years, male, asian, stage:ii, alive, 20 days	0.0
TCGA-G3-A25S-01A	64 years, male, white, stage:i, dead, 416 days	0.0
TCGA-K7-AAU7-01A	61 years, male, white, stage:ii, alive, 359 days	0.0
TCGA-MR-A520-01A	58 years, male, white, stage:i, alive, 229 days	0.0
TCGA-DD-A1EF-01A	57 years, female, white, stage:i, dead, 394 days	0.0
TCGA-T1-A6J8-01A	68 years, male, white, alive, 23 days	0.0
TCGA-DD-AACL-01A	66 years, female, asian, stage:i, dead, 107 days	0.0
TCGA-DD-A4NQ-01A	60 years, male, white, stage:ii, dead, 373 days	0.0
TCGA-RC-A6M6-01A	75 years, male, white, stage:ii, alive, 9 days	0.0
TCGA-ED-A66X-01A	35 years, male, asian, stage:iiia, alive, 406 days	0.0
TCGA-ZP-A9D2-01A	51 years, male, white, dead, 765 days	0.0
TCGA-BC-A69H-01A	64 years, male, white, stage:ii, alive, 444 days	0.0
TCGA-ZP-A9D1-01A	56 years, female, white, alive, 21 days	0.0
TCGA-DD-A4NH-01A	65 years, female, white, stage:iiib, alive, 917 days	0.0
TCGA-DD-AADW-01A	48 years, male, asian, stage:i, alive, 587 days	0.0
TCGA-DD-A1EC-01A	20 years, female, white, stage:i, alive, 602 days	0.0
TCGA-DD-AA3A-01A	81 years, female, white, stage:i, dead, 410 days	0.0
TCGA-GJ-A6C0-01A	75 years, female, white, stage:ii, dead, 31 days	0.0
TCGA-G3-A5SK-01A	58 years, male, white, stage:i, alive, 744 days	0.0
TCGA-DD-A1EI-01A	46 years, male, asian, stage:i, alive, 183 days	0.0
TCGA-FV-A3I0-01A	76 years, female, white, stage:ii, alive, 848 days	0.0
TCGA-CC-5264-01A	71 years, male, asian, stage:iiia, dead, 102 days	0.0
TCGA-ED-A66Y-01A	51 years, female, asian, stage:iiia, dead, 296 days	0.0
TCGA-G3-A3CH-01A	53 years, male, asian, stage:iiia, alive, 780 days	0.0
TCGA-FV-A3I1-01A	female, white, stage:ii, dead, 247 days	0.0
TCGA-MI-A75E-01A	61 years, male, white, stage:iiic, alive, 507 days	0.0
TCGA-K7-A6G5-01A	66 years, male, white, stage:i, alive, 512 days	0.0
TCGA-DD-A1EJ-01A	71 years, female, white, stage:iiic, dead, 1005 days	0.0
TCGA-DD-AACO-01A	40 years, male, asian, stage:i, alive, 1876 days	0.0

TCGA-CC-A7IJ-01A	56 years, male, asian, stage:ii, alive, 382 days	0.0
TCGA-DD-AADL-01A	58 years, male, asian, stage:i, alive, 636 days	0.0
TCGA-DD-AAW1-01A	55 years, male, asian, stage:iiia, alive, 1989 days	0.0
TCGA-RC-A7S9-01A	47 years, female, asian, stage:i, alive, 640 days	0.0
TCGA-WQ-A9G7-01A	female, white, alive, 30 days	0.0
TCGA-G3-A25Y-01A	52 years, female, asian, stage:i, dead, 452 days	0.0
TCGA-CC-5261-01A	44 years, male, asian, stage:ii, dead, 97 days	0.0
TCGA-2Y-A9GV-01A	54 years, female, white, stage:i, dead, 2532 days	0.0
TCGA-DD-AACE-01A	62 years, male, asian, stage:i, alive, 2184 days	0.0
TCGA-DD-AAD2-01A	66 years, male, asian, stage:i, alive, 658 days	0.0
TCGA-DD-AAD1-01A	51 years, female, asian, stage:i, alive, 564 days	0.0
TCGA-DD-AADD-01A	51 years, male, asian, stage:i, alive, 1231 days	0.0
TCGA-5C-A9VH-01A	70 years, male, white, stage:i, alive, 322 days	0.0
TCGA-ED-A5KG-01A	60 years, female, asian, stage:ii, alive, 854 days	0.0
TCGA-XR-A8TD-01A	49 years, female, white, stage:iiib, alive, 1030 days	0.0
TCGA-KR-A7K8-01A	57 years, male, stage:i, alive, 906 days	0.0
TCGA-DD-AACW-01A	43 years, male, asian, stage:i, alive, 1424 days	0.0
TCGA-G3-AAUZ-01A	48 years, male, stage:i, alive, 480 days	0.0
TCGA-2Y-A9GW-01A	64 years, male, white, stage:i, dead, 1271 days	0.0
TCGA-CC-A8HV-01A	51 years, female, asian, stage:ii, dead, 279 days	0.0
TCGA-ZP-A9CV-01A	59 years, male, white, dead, 1088 days	0.0
TCGA-MR-A8JO-01A	34 years, male, white, stage:i, alive, 330 days	0.0
TCGA-DD-A1EA-01A	68 years, male, asian, stage:ii, alive, 2415 days	0.0
TCGA-CC-A8HS-01A	18 years, male, asian, stage:iiic, dead, 300 days	0.0
TCGA-2Y-A9GX-01A	68 years, male, white, stage:i, alive, 2442 days	0.0
TCGA-KR-A7K0-01A	65 years, male, white, stage:i, dead, 65 days	0.0
TCGA-YA-A8S7-01A	69 years, male, white, stage:iiia, dead, 412 days	0.0
TCGA-CC-A7IF-01A	59 years, male, asian, stage:iiia, dead, 649 days	0.0
TCGA-DD-AADB-01A	51 years, male, asian, stage:i, alive, 1242 days	0.0
TCGA-DD-A3A1-01A	65 years, male, stage:iiia, dead, 233 days	0.0
TCGA-EP-A12J-01A	63 years, male, black or african american, stage:i, alive, 570 days	0.0
TCGA-UB-A7MD-01A	67 years, male, black or african american, stage:i, dead, 52 days	0.0
TCGA-2Y-A9GZ-01A	82 years, female, white, stage:ii, dead, 848 days	0.0
TCGA-ED-A7XP-01A	53 years, female, asian, stage:ii, alive, 400 days	0.0
TCGA-DD-AACF-01A	68 years, male, asian, stage:i, dead, 365 days	0.0
TCGA-DD-AAEI-01A	72 years, male, asian, stage:i, alive, 1531 days	0.0
TCGA-CC-A9FS-01A	55 years, male, asian, stage:ii, alive, 211 days	0.0
TCGA-2Y-A9H0-01A	49 years, male, white, stage:iiia, alive, 3675 days	0.0
TCGA-MI-A75I-01A	61 years, male, black or african american, alive, 630 days	0.0
TCGA-DD-A4ND-01A	56 years, female, white, stage:i, alive, 2746 days	0.0
TCGA-DD-A4NR-01A	85 years, female, white, stage:i, dead, 9 days	0.0
TCGA-UB-A7MB-01A	24 years, male, white, stage:ii, alive, 601 days	0.0
TCGA-CC-A7II-01A	55 years, male, asian, stage:iiia, alive, 399 days	0.0

TCGA-CC-A3M9-01A	45 years, male, asian, stage:iiia, dead, 300 days	0.0
TCGA-G3-A7M6-01A	60 years, female, white, stage:i, alive, 632 days	0.0
TCGA-DD-AAVQ-01A	38 years, male, asian, stage:i, alive, 2728 days	0.0
TCGA-FV-A3R3-01A	38 years, female, white, stage:i, dead, 366 days	0.0
TCGA-DD-A73C-01A	65 years, female, white, stage:iiia, alive, 701 days	0.0
TCGA-BC-A216-01A	62 years, female, white, stage:iiia, alive, 1351 days	0.0
TCGA-ED-A7PZ-01A	61 years, male, asian, stage:ii, alive, 6 days	0.0
TCGA-DD-A73B-01A	72 years, female, white, stage:i, dead, 283 days	0.0
TCGA-BC-A3KG-01A	68 years, female, white, stage:ii, alive, 680 days	0.0
TCGA-DD-A39Y-01A	67 years, male, asian, stage:i, dead, 171 days	0.0
TCGA-BC-A10R-01A	66 years, female, white, dead, 308 days	0.0
TCGA-EP-A2KB-01A	46 years, female, white, stage:i, dead, 596 days	0.0
TCGA-WX-AA46-01A	62 years, male, white, stage:ii, alive, 756 days	0.0
TCGA-QA-A7B7-01A	48 years, male, black or african american, stage:ii, alive, 94 days	0.0
TCGA-DD-A4NI-01A	67 years, male, white, stage:ii, alive, 816 days	0.0
TCGA-UB-A7MC-01A	59 years, male, white, stage:iiia, alive, 500 days	0.0
TCGA-DD-A11C-01A	69 years, male, white, stage:i, alive, 662 days	0.0
TCGA-DD-A1ED-01A	68 years, male, white, stage:i, alive, 2301 days	0.0
TCGA-G3-A3CK-01A	61 years, male, asian, stage:i, alive, 585 days	0.0
TCGA-BD-A3EP-01A	76 years, female, black or african american, stage:i, alive, 409 days	0.0
TCGA-ZP-A9D4-01A	64 years, female, white, alive, 395 days	0.0
TCGA-G3-AAV3-01A	58 years, female, white, stage:ii, alive, 412 days	0.0
TCGA-ED-A7PY-01A	20 years, female, asian, stage:ii, alive, 390 days	0.0
TCGA-DD-AACP-01A	65 years, male, asian, stage:i, alive, 415 days	0.0
TCGA-DD-A1EL-01A	23 years, male, black or african american, stage:ii, dead, 415 days	0.0
TCGA-LG-A6GG-01A	79 years, female, white, stage:ii, alive, 387 days	0.0
TCGA-DD-A3A7-01A	67 years, male, stage:iiib, dead, 419 days	0.0
TCGA-ED-A627-01A	74 years, male, white, stage:i, alive, 423 days	0.0
TCGA-DD-AADC-01A	53 years, male, asian, stage:i, dead, 425 days	0.0
TCGA-LG-A9QC-01A	48 years, male, white, stage:i, alive, 425 days	0.0
TCGA-G3-A7M8-01A	31 years, male, asian, stage:i, alive, 430 days	0.0
TCGA-DD-AACQ-01A	50 years, male, asian, stage:ii, dead, 432 days	0.0
TCGA-DD-AADS-01A	63 years, male, asian, stage:i, alive, 474 days	0.0
TCGA-DD-AADQ-01A	59 years, male, asian, stage:ii, alive, 436 days	0.0
TCGA-ES-A2HT-01A	54 years, male, black or african american, stage:i, dead, 438 days	0.0
TCGA-BC-A69I-01A	69 years, male, white, stage:i, alive, 387 days	0.0
TCGA-G3-A7M5-01A	76 years, male, asian, stage:i, alive, 447 days	0.0
TCGA-5R-AA1D-01A	17 years, female, white, stage:iiia, alive, 449 days	0.0
TCGA-DD-AACD-01A	48 years, male, asian, stage:i, dead, 381 days	0.0
TCGA-DD-AADO-01A	55 years, male, asian, stage:i, alive, 453 days	0.0
TCGA-DD-AADP-01A	45 years, male, asian, stage:i, alive, 458 days	0.0
TCGA-RC-A7SH-01A	42 years, male, asian, stage:ii, alive, 468 days	0.0
TCGA-DD-AACG-01A	52 years, male, asian, stage:ii, dead, 469 days	0.0

TCGA-RC-A7SK-01A	59 years, male, asian, stage:i, alive, 472 days	0.0
TCGA-G3-A3CJ-01A	52 years, male, american indian or alaska native, stage:ii, alive, 594 days	0.0
TCGA-WQ-AB4B-01A	62 years, male, white, stage:ii, alive, 395 days	0.0
TCGA-DD-AAC9-01A	51 years, male, asian, stage:i, alive, 347 days	0.0
TCGA-ED-A7XO-01A	29 years, male, asian, stage:iiia, alive, 427 days	0.0
TCGA-DD-AAEB-01A	60 years, male, asian, stage:i, alive, 478 days	0.0
TCGA-LG-A9QD-01A	68 years, male, white, stage:iiia, alive, 366 days	0.0
TCGA-CC-A7IH-01A	58 years, male, asian, stage:iiia, alive, 365 days	0.0
TCGA-EP-A3RK-01A	73 years, male, white, stage:iiia, alive, 363 days	0.0
TCGA-G3-A5SM-01A	58 years, male, white, stage:ii, alive, 520 days	0.0
TCGA-5R-AA1C-01A	57 years, male, white, stage:ii, alive, 520 days	0.0
TCGA-DD-A3A3-01A	45 years, male, asian, stage:i, dead, 535 days	0.0
TCGA-O8-A75V-01A	54 years, male, stage:i, alive, 538 days	0.0
TCGA-G3-A7M7-01A	65 years, male, white, stage:i, alive, 361 days	0.0
TCGA-DD-AAE1-01A	52 years, male, asian, stage:i, alive, 552 days	0.0
TCGA-DD-AADU-01A	60 years, male, asian, stage:ii, alive, 554 days	0.0
TCGA-G3-AAV1-01A	51 years, male, asian, stage:iiic, dead, 359 days	0.0
TCGA-DD-AAE0-01A	45 years, female, asian, stage:iiia, alive, 555 days	0.0
TCGA-WX-AA47-01A	33 years, female, white, stage:iiia, dead, 556 days	0.0
TCGA-DD-A39Z-01A	43 years, female, stage:ii, dead, 601 days	0.0
TCGA-DD-A1EK-01A	64 years, female, white, stage:ivb, dead, 558 days	0.0
TCGA-2Y-A9H6-01A	68 years, female, white, stage:i, alive, 357 days	0.0
TCGA-G3-AAV5-01A	67 years, male, white, stage:ii, alive, 354 days	0.0
TCGA-DD-AAE3-01A	50 years, male, asian, stage:i, alive, 566 days	0.0
TCGA-DD-A1EE-01A	73 years, male, white, stage:iiia, dead, 349 days	0.0
TCGA-DD-AADV-01A	50 years, male, asian, stage:i, alive, 574 days	0.0
TCGA-DD-AAEA-01A	65 years, male, asian, stage:i, alive, 575 days	0.0
TCGA-RC-A7SF-01A	66 years, male, asian, stage:i, alive, 579 days	0.0
TCGA-FV-A2QR-01A	male, white, stage:i, dead, 581 days	0.0
TCGA-CC-A5UC-01A	63 years, male, asian, stage:iiia, dead, 347 days	0.0
TCGA-RC-A7SB-01A	53 years, male, asian, stage:ii, alive, 588 days	0.0
TCGA-G3-AAV2-01A	50 years, male, white, stage:i, alive, 372 days	0.0
TCGA-G3-AAV0-01A	58 years, male, asian, stage:i, alive, 476 days	0.0
TCGA-MI-A75G-01A	63 years, male, white, stage:ii, alive, 698 days	0.0
TCGA-BC-A10T-01A	76 years, male, white, dead, 837 days	0.0
TCGA-PD-A5DF-01A	58 years, female, white, stage:iiib, dead, 639 days	0.0
TCGA-CC-A8HU-01A	39 years, female, asian, stage:iiia, dead, 344 days	0.0
TCGA-DD-AAE4-01A	49 years, female, asian, stage:i, alive, 608 days	0.0
TCGA-EP-A26S-01A	70 years, male, white, stage:i, alive, 608 days	0.0
TCGA-DD-A3A4-01A	37 years, male, white, stage:iiia, dead, 612 days	0.0
TCGA-WX-AA44-01A	64 years, female, white, stage:i, alive, 615 days	0.0
TCGA-G3-A5SL-01A	70 years, male, white, stage:ii, alive, 621 days	0.0
TCGA-ZS-A9CG-01A	55 years, male, white, stage:ii, alive, 341 days	0.0

TCGA-5C-A9VG-01A	58 years, male, white, stage:ii, alive, 328 days	0.0
TCGA-K7-A5RF-01A	64 years, male, white, stage:i, alive, 631 days	0.0
TCGA-UB-AA0U-01A	60 years, male, white, stage:ii, alive, 327 days	0.0
TCGA-2Y-A9H8-01A	85 years, female, white, dead, 633 days	0.0
TCGA-UB-AA0V-01A	69 years, female, white, stage:i, alive, 314 days	0.0
TCGA-XR-A8TF-01A	74 years, male, white, stage:i, dead, 693 days	0.0
TCGA-WJ-A86L-01A	68 years, female, white, stage:i, alive, 345 days	0.0
TCGA-CC-A5UD-01A	45 years, male, asian, stage:iiia, dead, 304 days	0.0
TCGA-DD-A39V-01A	77 years, male, white, stage:ii, dead, 643 days	0.0
TCGA-DD-AAE7-01A	72 years, male, asian, stage:i, alive, 644 days	0.0
TCGA-EP-A3JL-01A	76 years, male, white, stage:i, alive, 303 days	0.0
TCGA-G3-A25Z-01A	58 years, male, asian, stage:i, alive, 655 days	0.0
TCGA-CC-A7IG-01A	47 years, male, asian, stage:ii, dead, 299 days	0.0
TCGA-DD-A4NE-01A	75 years, female, white, stage:iiia, dead, 660 days	0.0
TCGA-G3-A6UC-01A	65 years, male, white, stage:iiib, alive, 671 days	0.0
TCGA-DD-AAD6-01A	66 years, male, asian, stage:iiia, alive, 672 days	0.0
TCGA-G3-A3CG-01A	80 years, male, white, stage:i, alive, 673 days	0.0
TCGA-MI-A75C-01A	64 years, male, white, stage:i, alive, 291 days	0.0
TCGA-ES-A2HS-01A	80 years, male, white, stage:i, dead, 688 days	0.0
TCGA-DD-AAE2-01A	51 years, male, asian, stage:i, alive, 638 days	0.0
TCGA-DD-A73D-01A	68 years, female, white, stage:ii, alive, 693 days	0.0
TCGA-DD-AAED-01A	51 years, male, asian, stage:i, alive, 763 days	0.0
TCGA-CC-5259-01A	60 years, female, asian, stage:iiic, alive, 250 days	0.0
TCGA-4R-AA8I-01A	66 years, male, white, stage:ii, dead, 262 days	0.0
TCGA-2Y-A9H5-01A	59 years, female, white, stage:i, dead, 555 days	0.0
TCGA-ZP-A9CZ-01A	72 years, male, asian, alive, 706 days	0.0
TCGA-BC-A10Y-01A	76 years, male, white, dead, 711 days	0.0
TCGA-CC-A7IK-01A	59 years, male, asian, stage:iiia, dead, 262 days	0.0
TCGA-DD-AAE9-01A	69 years, male, asian, stage:i, alive, 722 days	0.0
TCGA-2Y-A9GS-01A	58 years, male, white, dead, 724 days	0.0
TCGA-DD-A73A-01A	71 years, male, white, stage:i, alive, 728 days	0.0
TCGA-FV-A2QQ-01A	80 years, male, white, stage:i, alive, 729 days	0.0
TCGA-2Y-A9HB-01A	66 years, male, stage:i, alive, 260 days	0.0
TCGA-MI-A75H-01A	77 years, male, white, alive, 747 days	0.0
TCGA-2Y-A9GY-01A	64 years, female, white, stage:ii, dead, 757 days	0.0
TCGA-2Y-A9H9-01A	70 years, male, white, stage:i, alive, 697 days	0.0
TCGA-CC-A7IL-01A	61 years, male, asian, stage:iiia, dead, 278 days	0.0
TCGA-G3-A5SI-01A	44 years, male, asian, stage:ii, dead, 768 days	0.0
TCGA-BC-A10X-01A	52 years, female, white, stage:iiia, dead, 770 days	0.0
TCGA-CC-A9FW-01A	68 years, male, asian, stage:iiia, alive, 248 days	0.0
TCGA-ZP-A9CY-01A	66 years, female, white, alive, 782 days	0.0
TCGA-DD-AAEH-01A	73 years, male, asian, stage:i, alive, 784 days	0.0
TCGA-NI-A8LF-01A	74 years, male, white, stage:i, alive, 799 days	0.0

TCGA-DD-A4NG-01A	77 years, male, white, stage:iiia, dead, 802 days	0.0
TCGA-DD-AAEE-01A	55 years, male, asian, stage:i, alive, 810 days	0.0
TCGA-ED-A4XI-01A	58 years, male, asian, stage:ii, alive, 819 days	0.0
TCGA-DD-A39W-01A	29 years, female, white, stage:iii, dead, 827 days	0.0
TCGA-KR-A7K2-01A	64 years, male, white, stage:i, alive, 829 days	0.0
TCGA-ED-A8O5-01A	59 years, female, asian, stage:iiia, alive, 406 days	0.0
TCGA-UB-A7ME-01A	51 years, male, asian, stage:i, alive, 486 days	0.0
TCGA-DD-A1EH-01A	23 years, male, white, stage:iii, alive, 1495 days	0.0
TCGA-DD-A119-01A	40 years, male, asian, stage:iv, dead, 223 days	0.0
TCGA-BD-A3ER-01A	62 years, male, white, stage:ii, alive, 1115 days	0.0
TCGA-FV-A3R2-01A	75 years, male, white, stage:i, dead, 194 days	0.0
TCGA-G3-A25V-01A	68 years, male, white, stage:i, alive, 860 days	0.0
TCGA-DD-AACZ-01A	63 years, female, asian, stage:i, dead, 171 days	0.0
TCGA-XR-A8TG-01A	59 years, male, white, stage:i, alive, 898 days	0.0
TCGA-DD-AADN-01A	59 years, male, asian, stage:i, alive, 898 days	0.0
TCGA-DD-A4NN-01A	56 years, female, white, stage:i, dead, 899 days	0.0
TCGA-DD-AACX-01A	66 years, male, asian, stage:ii, alive, 170 days	0.0
TCGA-DD-AAE6-01A	59 years, female, asian, stage:i, alive, 141 days	0.0
TCGA-CC-A8HT-01A	74 years, male, asian, stage:iiia, dead, 140 days	0.0
TCGA-XR-A8TE-01A	16 years, male, white, stage:iiia, alive, 925 days	0.0
TCGA-DD-A4NJ-01A	54 years, female, white, stage:ii, alive, 928 days	0.0
TCGA-DD-A4NF-01A	72 years, male, white, stage:i, alive, 942 days	0.0
TCGA-KR-A7K7-01A	61 years, female, white, stage:ii, alive, 951 days	0.0
TCGA-DD-A3A9-01A	64 years, female, white, stage:ivb, dead, 931 days	0.0
TCGA-DD-A4NB-01A	25 years, male, white, stage:i, alive, 989 days	0.0
TCGA-DD-AAD0-01A	73 years, female, asian, stage:i, alive, 137 days	0.0
TCGA-CC-5258-01A	48 years, male, asian, stage:ii, dead, 129 days	0.0
TCGA-DD-AADF-01A	64 years, female, asian, stage:i, dead, 115 days	0.0
TCGA-CC-5262-01A	67 years, male, asian, stage:iiic, dead, 103 days	0.0
TCGA-DD-AADJ-01A	70 years, female, asian, stage:i, alive, 1066 days	0.0
TCGA-DD-AAEK-01A	51 years, male, asian, stage:ii, alive, 1067 days	0.0
TCGA-DD-A73F-01A	77 years, female, white, stage:i, alive, 1085 days	0.0
TCGA-DD-AADI-01A	43 years, female, asian, stage:i, alive, 1085 days	0.0
TCGA-ZP-A9D0-01A	67 years, female, black or african american, alive, 1091 days	0.0
TCGA-2Y-A9HA-01A	70 years, male, white, stage:ii, dead, 36 days	0.0
TCGA-BC-A10Z-01A	62 years, female, white, stage:i, dead, 34 days	0.0
TCGA-UB-A7MF-01A	57 years, male, white, stage:iiia, dead, 214 days	0.0
TCGA-HP-A5MZ-01A	62 years, male, stage:i, dead, 91 days	0.0
TCGA-DD-A3A8-01A	75 years, male, white, stage:ii, dead, 11 days	0.0
TCGA-ED-A8O6-01A	50 years, female, asian, stage:iiia, dead, 56 days	0.0
TCGA-3K-AAZ8-01A	65 years, male, black or african american, stage:iiib, alive, 396 days	0.0
TCGA-ZS-A9CE-01A	79 years, female, white, stage:ii, alive, 1241 days	0.0
TCGA-DD-AADA-01A	66 years, female, asian, stage:i, alive, 1233 days	0.0

TCGA-GJ-A9DB-01A	68 years, male, white, stage:i, dead, 67 days	0.0
TCGA-DD-AAD8-01A	73 years, female, asian, stage:i, alive, 1219 days	0.0
TCGA-2Y-A9H1-01A	58 years, male, white, stage:i, dead, 1229 days	0.0
TCGA-DD-A4NK-01A	80 years, female, white, stage:iiia, dead, 1210 days	0.0
TCGA-2Y-A9H7-01A	81 years, female, white, stage:i, alive, 1168 days	0.0
TCGA-DD-A11A-01A	67 years, male, black or african american, stage:i, alive, 79 days	0.0
TCGA-HP-A5N0-01A	88 years, female, dead, 1147 days	0.0
TCGA-G3-AAV6-01A	53 years, female, white, stage:iiia, dead, 65 days	0.0
TCGA-DD-AAD3-01A	43 years, male, asian, stage:i, alive, 1295 days	0.0
TCGA-DD-AACN-01A	32 years, male, asian, stage:i, alive, 1302 days	0.0
TCGA-RG-A7D4-01A	69 years, male, black or african american, stage:ii, alive, 1098 days	0.0
TCGA-XR-A8TC-01A	43 years, female, white, stage:i, alive, 1339 days	0.0
TCGA-DD-AAD5-01A	54 years, male, asian, stage:i, alive, 1345 days	0.0
TCGA-5R-AAAM-01A	65 years, female, white, stage:ii, dead, 46 days	0.0
TCGA-BD-A2L6-01A	69 years, male, white, alive, 1363 days	0.0
TCGA-DD-A1EG-01A	77 years, male, white, stage:i, dead, 1372 days	0.0
TCGA-ZS-A9CD-01A	73 years, male, white, stage:ii, dead, 1386 days	0.0
TCGA-BC-A217-01A	75 years, female, white, stage:ii, dead, 1397 days	0.0
TCGA-BC-A10S-01A	81 years, male, white, dead, 1423 days	0.0
TCGA-DD-A73E-01A	66 years, male, white, stage:i, alive, 44 days	0.0
TCGA-DD-AACY-01A	61 years, male, asian, stage:i, alive, 1450 days	0.0
TCGA-2Y-A9H4-01A	68 years, male, black or african american, stage:i, alive, 1452 days	0.0
TCGA-DD-AADG-01A	70 years, male, asian, stage:iiia, alive, 1145 days	0.0
TCGA-2Y-A9H3-01A	45 years, male, white, stage:ii, alive, 1516 days	0.0
TCGA-DD-A3A2-01A	76 years, female, white, stage:i, dead, 2131 days	0.0
TCGA-BC-A10U-01A	69 years, male, white, dead, 837 days	0.0
TCGA-BW-A5NO-01A	50 years, male, black or african american, stage:iiia, alive, 20 days	0.0
TCGA-DD-A11D-01A	57 years, female, white, stage:i, dead, 1560 days	0.0
TCGA-DD-AACT-01A	69 years, female, asian, stage:i, alive, 1562 days	0.0
TCGA-DD-AACU-01A	59 years, male, asian, stage:i, alive, 1567 days	0.0
TCGA-DD-AAVX-01A	38 years, male, asian, stage:ii, alive, 1570 days	0.0
TCGA-RC-A6M4-01A	74 years, female, white, stage:iiia, alive, 22 days	0.0
TCGA-DD-A116-01A	68 years, male, asian, stage:iiia, dead, 1622 days	0.0
TCGA-2Y-A9GT-01A	51 years, male, white, stage:i, dead, 1624 days	0.0
TCGA-DD-AAW3-01A	69 years, male, asian, stage:i, alive, 1633 days	0.0
TCGA-G3-A25U-01A	63 years, female, asian, stage:i, alive, 1636 days	0.0
TCGA-DD-AACC-01A	61 years, male, asian, stage:i, dead, 1685 days	0.0
TCGA-DD-A4NL-01A	46 years, male, white, stage:i, alive, 1711 days	0.0
TCGA-DD-A1EB-01A	72 years, female, stage:i, alive, 2017 days	0.0
TCGA-EP-A2KC-01A	62 years, male, black or african american, stage:i, dead, 19 days	0.0
TCGA-DD-AACV-01A	53 years, male, asian, stage:i, alive, 1531 days	0.0
TCGA-NI-A4U2-01A	71 years, male, white, stage:iiia, dead, 1791 days	0.0
TCGA-DD-AACS-01A	39 years, male, asian, stage:i, alive, 1804 days	0.0

TCGA-DD-AAVS-01A	56 years, male, asian, stage:i, alive, 1823 days	0.0
TCGA-FV-A23B-01A	70 years, female, white, stage:ii, dead, 1852 days	0.0
TCGA-DD-AAW2-01A	69 years, male, asian, stage:i, alive, 1855 days	0.0
TCGA-DD-AAC8-01A	72 years, male, asian, stage:i, dead, 16 days	0.0
TCGA-DD-AAVZ-01A	38 years, male, asian, stage:i, alive, 1900 days	0.0
TCGA-2Y-A9GU-01A	55 years, female, white, stage:i, alive, 1939 days	0.0
TCGA-DD-AAVY-01A	56 years, male, asian, stage:iiia, alive, 1970 days	0.0
TCGA-RC-A6M5-01A	20 years, female, white, stage:iva, alive, 15 days	0.0
TCGA-DD-AAW0-01A	54 years, male, asian, stage:i, alive, 2015 days	0.0
TCGA-CC-A7IE-01A	57 years, male, asian, stage:iiia, dead, 217 days	0.0
TCGA-G3-AAV4-01A	83 years, female, white, stage:i, dead, 27 days	0.0
TCGA-DD-A39X-01A	78 years, female, white, stage:i, dead, 1694 days	0.0
TCGA-DD-A11B-01A	73 years, male, white, stage:i, dead, 14 days	0.0
TCGA-FV-A4ZP-01A	78 years, male, white, stage:iiia, dead, 2486 days	0.0
TCGA-DD-AAVU-01A	46 years, male, asian, stage:ii, alive, 2202 days	0.0
TCGA-DD-A4NO-01A	66 years, male, white, stage:i, alive, 2245 days	0.0
TCGA-DD-AACA-01A	65 years, male, asian, stage:i, alive, 2301 days	0.0
TCGA-DD-AAVW-01A	35 years, male, asian, stage:i, alive, 2317 days	0.0
TCGA-DD-AACB-01A	74 years, female, asian, stage:i, alive, 2324 days	0.0
TCGA-DD-A4NV-01A	61 years, male, white, stage:iiia, alive, 2398 days	0.0
TCGA-ZS-A9CF-01A	64 years, male, white, stage:ii, alive, 2412 days	0.0
TCGA-DD-AADM-01A	58 years, male, asian, stage:ii, dead, 12 days	0.0
TCGA-DD-A113-01A	55 years, female, white, stage:ii, alive, 2425 days	0.0
TCGA-FV-A4ZQ-01A	52 years, male, white, stage:i, alive, 12 days	0.0
TCGA-DD-A4NS-01A	62 years, female, white, stage:i, dead, 2456 days	0.0
TCGA-DD-AACJ-01A	75 years, male, asian, stage:ii, alive, 2102 days	0.0
TCGA-DD-AAVR-01A	44 years, male, asian, stage:i, alive, 2513 days	0.0
TCGA-CC-A123-01A	24 years, female, asian, stage:iiia, alive, 219 days	0.0
TCGA-FV-A496-01A	84 years, female, white, stage:i, alive, 10 days	0.0
TCGA-DD-A115-01A	53 years, male, white, stage:iiia, dead, 2542 days	0.0
TCGA-DD-AACK-01A	70 years, male, asian, stage:i, alive, 9 days	0.0
TCGA-BC-A3KF-01A	66 years, female, white, stage:i, alive, 8 days	0.0
TCGA-DD-AAVP-01A	48 years, male, asian, stage:i, alive, 2752 days	0.0
TCGA-DD-A3A5-01A	66 years, female, white, stage:iii, dead, 3125 days	0.0
TCGA-DD-A3A6-01A	72 years, female, white, stage:ii, dead, 3258 days	0.0
TCGA-DD-A4NP-01A	32 years, male, white, stage:i, alive, 3308 days	0.0
TCGA-DD-A118-01A	77 years, female, white, stage:ii, alive, 3437 days	0.0
TCGA-DD-A73G-01A	73 years, female, white, stage:i, alive, 3478 days	0.0
TCGA-BC-A110-01A	51 years, female, black or african american, dead, 2116 days	0.0
TCGA-DD-AADR-01A	58 years, male, asian, stage:i, alive, 2028 days	0.0
TCGA-FV-A495-01A	51 years, female, white, stage:ii, alive, 1 days	0.0

TRPV6 RNA expression (N=365)

Sample	Description	FPKM
TCGA-DD-A114-01A	42 years, male, black or african american, stage:ii, dead, 1149 days	8.1
TCGA-G3-AAV7-01A	38 years, male, asian, stage:ii, alive, 361 days	5.8
TCGA-G3-A25X-01A	73 years, male, asian, stage:ii, alive, 1779 days	5.6
TCGA-CC-5263-01A	35 years, male, asian, stage:iiia, dead, 129 days	4.7
TCGA-CC-5260-01A	61 years, female, asian, stage:iiic, dead, 87 days	3.1
TCGA-CC-A3MA-01A	61 years, male, asian, stage:iiia, dead, 303 days	2.8
TCGA-CC-A5UE-01A	48 years, male, asian, stage:iiib, dead, 272 days	2.6
TCGA-DD-AADY-01A	55 years, female, asian, stage:i, alive, 555 days	2.6
TCGA-2Y-A9H2-01A	64 years, female, white, stage:i, alive, 1731 days	2.3
TCGA-BC-A5W4-01A	69 years, male, white, stage:iiia, dead, 547 days	1.9
TCGA-ED-A97K-01A	54 years, male, asian, stage:iiia, alive, 6 days	1.9
TCGA-FV-A3I0-01A	76 years, female, white, stage:ii, alive, 848 days	1.6
TCGA-DD-A4NS-01A	62 years, female, white, stage:i, dead, 2456 days	1.3
TCGA-DD-AADK-01A	68 years, female, asian, stage:ii, alive, 1049 days	1.1
TCGA-WQ-A9G7-01A	female, white, alive, 30 days	1.0
TCGA-DD-AAD1-01A	51 years, female, asian, stage:i, alive, 564 days	0.9
TCGA-G3-A3CI-01A	71 years, male, white, stage:i, alive, 180 days	0.9
TCGA-DD-A4NA-01A	67 years, female, white, stage:iiic, alive, 1008 days	0.9
TCGA-ED-A627-01A	74 years, male, white, stage:i, alive, 423 days	0.9
TCGA-ED-A82E-01A	60 years, female, asian, stage:iiia, alive, 408 days	0.8
TCGA-G3-A25T-01A	45 years, female, white, stage:iiia, alive, 1553 days	0.8
TCGA-DD-AACO-01A	40 years, male, asian, stage:i, alive, 1876 days	0.7
TCGA-BC-A10Q-01A	72 years, female, white, dead, 1135 days	0.7
TCGA-CC-A3MC-01A	54 years, male, asian, stage:iiia, alive, 363 days	0.7
TCGA-K7-AAU7-01A	61 years, male, white, stage:ii, alive, 359 days	0.6
TCGA-DD-AACI-01A	69 years, male, asian, stage:ii, alive, 1618 days	0.5
TCGA-UB-AA0V-01A	69 years, female, white, stage:i, alive, 314 days	0.5
TCGA-5C-AAPD-01A	61 years, male, asian, stage:ii, alive, 20 days	0.5
TCGA-K7-A5RF-01A	64 years, male, white, stage:i, alive, 631 days	0.5
TCGA-EP-A2KA-01A	52 years, female, white, stage:iiia, dead, 627 days	0.5
TCGA-ED-A7PX-01A	48 years, female, asian, stage:ii, alive, 6 days	0.4
TCGA-BC-A8YO-01A	66 years, female, white, stage:iiic, alive, 562 days	0.4
TCGA-DD-AACH-01A	69 years, male, asian, stage:ii, dead, 195 days	0.4
TCGA-BC-A10W-01A	50 years, male, asian, dead, 91 days	0.3
TCGA-DD-A4ND-01A	56 years, female, white, stage:i, alive, 2746 days	0.3
TCGA-UB-A7MA-01A	62 years, female, white, stage:ii, alive, 848 days	0.2
TCGA-BC-A112-01A	80 years, male, white, dead, 153 days	0.2
TCGA-DD-A4NH-01A	65 years, female, white, stage:iiib, alive, 917 days	0.2
TCGA-DD-AAVV-01A	56 years, male, asian, stage:ii, alive, 2455 days	0.2
TCGA-MI-A75E-01A	61 years, male, white, stage:iiic, alive, 507 days	0.2

TCGA-2Y-A9H5-01A	59 years, female, white, stage:i, dead, 555 days	0.2
TCGA-DD-AA3A-01A	81 years, female, white, stage:i, dead, 410 days	0.2
TCGA-DD-AAEG-01A	59 years, female, asian, stage:i, alive, 719 days	0.2
TCGA-GJ-A3OU-01A	59 years, male, white, stage:i, alive, 879 days	0.2
TCGA-ZP-A9D2-01A	51 years, male, white, dead, 765 days	0.2
TCGA-FV-A495-01A	51 years, female, white, stage:ii, alive, 1 days	0.2
TCGA-BC-4072-01B	74 years, female, white, stage:iiia, dead, 1490 days	0.1
TCGA-CC-A8HS-01A	18 years, male, asian, stage:iiic, dead, 300 days	0.1
TCGA-CC-A5UC-01A	63 years, male, asian, stage:iiia, dead, 347 days	0.1
TCGA-ED-A4XI-01A	58 years, male, asian, stage:ii, alive, 819 days	0.1
TCGA-DD-AADP-01A	45 years, male, asian, stage:i, alive, 458 days	0.1
TCGA-G3-A7M9-01A	70 years, male, white, stage:iiib, dead, 56 days	0.1
TCGA-BC-4073-01B	73 years, male, white, stage:iiia, alive, 849 days	0.1
TCGA-DD-AACL-01A	66 years, female, asian, stage:i, dead, 107 days	0.1
TCGA-G3-A5SJ-01A	59 years, male, white, stage:i, alive, 698 days	0.1
TCGA-DD-AAED-01A	51 years, male, asian, stage:i, alive, 763 days	0.1
TCGA-G3-A7M6-01A	60 years, female, white, stage:i, alive, 632 days	0.1
TCGA-DD-A4NE-01A	75 years, female, white, stage:iiia, dead, 660 days	0.1
TCGA-2Y-A9GX-01A	68 years, male, white, stage:i, alive, 2442 days	0.1
TCGA-DD-A1EI-01A	46 years, male, asian, stage:i, alive, 183 days	0.1
TCGA-CC-A7IG-01A	47 years, male, asian, stage:ii, dead, 299 days	0.1
TCGA-ED-A7XO-01A	29 years, male, asian, stage:iiia, alive, 427 days	0.1
TCGA-DD-AACQ-01A	50 years, male, asian, stage:ii, dead, 432 days	0.1
TCGA-DD-A39X-01A	78 years, female, white, stage:i, dead, 1694 days	0.1
TCGA-BC-A10X-01A	52 years, female, white, stage:iiia, dead, 770 days	0.1
TCGA-T1-A6J8-01A	68 years, male, white, alive, 23 days	0.1
TCGA-ED-A459-01A	47 years, male, asian, stage:ii, alive, 910 days	0.1
TCGA-BC-A110-01A	51 years, female, black or african american, dead, 2116 days	0.1
TCGA-UB-AA0U-01A	60 years, male, white, stage:ii, alive, 327 days	0.1
TCGA-DD-AADC-01A	53 years, male, asian, stage:i, dead, 425 days	0.1
TCGA-XR-A8TD-01A	49 years, female, white, stage:iiib, alive, 1030 days	0.1
TCGA-G3-A25Y-01A	52 years, female, asian, stage:i, dead, 452 days	0.1
TCGA-DD-AAD2-01A	66 years, male, asian, stage:i, alive, 658 days	0.1
TCGA-ED-A66Y-01A	51 years, female, asian, stage:iiia, dead, 296 days	0.1
TCGA-CC-A1HT-01A	50 years, male, asian, stage:iiia, dead, 101 days	0.1
TCGA-ZP-A9CY-01A	66 years, female, white, alive, 782 days	0.1
TCGA-DD-AACC-01A	61 years, male, asian, stage:i, dead, 1685 days	0.1
TCGA-MR-A520-01A	58 years, male, white, stage:i, alive, 229 days	0.1
TCGA-DD-AAE0-01A	45 years, female, asian, stage:iiia, alive, 555 days	0.1
TCGA-2Y-A9GS-01A	58 years, male, white, dead, 724 days	0.1
TCGA-WX-AA44-01A	64 years, female, white, stage:i, alive, 615 days	0.0
TCGA-G3-A25V-01A	68 years, male, white, stage:i, alive, 860 days	0.0
TCGA-DD-AAVR-01A	44 years, male, asian, stage:i, alive, 2513 days	0.0

TCGA-DD-AADL-01A	58 years, male, asian, stage:i, alive, 636 days	0.0
TCGA-KR-A7K0-01A	65 years, male, white, stage:i, dead, 65 days	0.0
TCGA-CC-A3MB-01A	36 years, male, asian, stage:iiia, dead, 315 days	0.0
TCGA-DD-A1ED-01A	68 years, male, white, stage:i, alive, 2301 days	0.0
TCGA-DD-AAE7-01A	72 years, male, asian, stage:i, alive, 644 days	0.0
TCGA-DD-AADW-01A	48 years, male, asian, stage:i, alive, 587 days	0.0
TCGA-G3-A5SM-01A	58 years, male, white, stage:ii, alive, 520 days	0.0
TCGA-KR-A7K2-01A	64 years, male, white, stage:i, alive, 829 days	0.0
TCGA-CC-A7II-01A	55 years, male, asian, stage:iiia, alive, 399 days	0.0
TCGA-UB-A7MD-01A	67 years, male, black or african american, stage:i, dead, 52 days	0.0
TCGA-DD-AACU-01A	59 years, male, asian, stage:i, alive, 1567 days	0.0
TCGA-RC-A6M6-01A	75 years, male, white, stage:ii, alive, 9 days	0.0
TCGA-MI-A75G-01A	63 years, male, white, stage:ii, alive, 698 days	0.0
TCGA-DD-AAW0-01A	54 years, male, asian, stage:i, alive, 2015 days	0.0
TCGA-RC-A7SB-01A	53 years, male, asian, stage:ii, alive, 588 days	0.0
TCGA-CC-5261-01A	44 years, male, asian, stage:ii, dead, 97 days	0.0
TCGA-DD-A1EC-01A	20 years, female, white, stage:i, alive, 602 days	0.0
TCGA-CC-A7IJ-01A	56 years, male, asian, stage:ii, alive, 382 days	0.0
TCGA-DD-AADI-01A	43 years, female, asian, stage:i, alive, 1085 days	0.0
TCGA-G3-A7M8-01A	31 years, male, asian, stage:i, alive, 430 days	0.0
TCGA-UB-A7ME-01A	51 years, male, asian, stage:i, alive, 486 days	0.0
TCGA-BC-A10R-01A	66 years, female, white, dead, 308 days	0.0
TCGA-GJ-A6C0-01A	75 years, female, white, stage:ii, dead, 31 days	0.0
TCGA-BD-A3ER-01A	62 years, male, white, stage:ii, alive, 1115 days	0.0
TCGA-CC-5264-01A	71 years, male, asian, stage:iiia, dead, 102 days	0.0
TCGA-DD-A4NV-01A	61 years, male, white, stage:iiia, alive, 2398 days	0.0
TCGA-ED-A8O5-01A	59 years, female, asian, stage:iiia, alive, 406 days	0.0
TCGA-DD-AACN-01A	32 years, male, asian, stage:i, alive, 1302 days	0.0
TCGA-K7-A5RG-01A	66 years, male, black or african american, stage:i, alive, 519 days	0.0
TCGA-ED-A8O6-01A	50 years, female, asian, stage:iiia, dead, 56 days	0.0
TCGA-CC-A8HT-01A	74 years, male, asian, stage:iiia, dead, 140 days	0.0
TCGA-BC-A10T-01A	76 years, male, white, dead, 837 days	0.0
TCGA-G3-A3CH-01A	53 years, male, asian, stage:iiia, alive, 780 days	0.0
TCGA-DD-AAC9-01A	51 years, male, asian, stage:i, alive, 347 days	0.0
TCGA-CC-5259-01A	60 years, female, asian, stage:iiic, alive, 250 days	0.0
TCGA-DD-AACG-01A	52 years, male, asian, stage:ii, dead, 469 days	0.0
TCGA-ED-A7PZ-01A	61 years, male, asian, stage:ii, alive, 6 days	0.0
TCGA-BD-A3EP-01A	76 years, female, black or african american, stage:i, alive, 409 days	0.0
TCGA-ED-A5KG-01A	60 years, female, asian, stage:ii, alive, 854 days	0.0
TCGA-ZP-A9D1-01A	56 years, female, white, alive, 21 days	0.0
TCGA-2Y-A9H0-01A	49 years, male, white, stage:iiia, alive, 3675 days	0.0
TCGA-XR-A8TC-01A	43 years, female, white, stage:i, alive, 1339 days	0.0
TCGA-K7-A6G5-01A	66 years, male, white, stage:i, alive, 512 days	0.0

TCGA-DD-A4NQ-01A	60 years, male, white, stage:ii, dead, 373 days	0.0
TCGA-4R-AA8I-01A	66 years, male, white, stage:ii, dead, 262 days	0.0
TCGA-CC-5262-01A	67 years, male, asian, stage:iiic, dead, 103 days	0.0
TCGA-DD-AAVQ-01A	38 years, male, asian, stage:i, alive, 2728 days	0.0
TCGA-FV-A3R3-01A	38 years, female, white, stage:i, dead, 366 days	0.0
TCGA-DD-A39Y-01A	67 years, male, asian, stage:i, dead, 171 days	0.0
TCGA-DD-AACW-01A	43 years, male, asian, stage:i, alive, 1424 days	0.0
TCGA-XR-A8TG-01A	59 years, male, white, stage:i, alive, 898 days	0.0
TCGA-HP-A5MZ-01A	62 years, male, stage:i, dead, 91 days	0.0
TCGA-2Y-A9GT-01A	51 years, male, white, stage:i, dead, 1624 days	0.0
TCGA-ED-A66X-01A	35 years, male, asian, stage:iiia, alive, 406 days	0.0
TCGA-2Y-A9GW-01A	64 years, male, white, stage:i, dead, 1271 days	0.0
TCGA-DD-A3A6-01A	72 years, female, white, stage:ii, dead, 3258 days	0.0
TCGA-BC-A69H-01A	64 years, male, white, stage:ii, alive, 444 days	0.0
TCGA-GJ-A9DB-01A	68 years, male, white, stage:i, dead, 67 days	0.0
TCGA-RC-A7S9-01A	47 years, female, asian, stage:i, alive, 640 days	0.0
TCGA-MI-A75C-01A	64 years, male, white, stage:i, alive, 291 days	0.0
TCGA-RC-A6M5-01A	20 years, female, white, stage:iva, alive, 15 days	0.0
TCGA-DD-A73F-01A	77 years, female, white, stage:i, alive, 1085 days	0.0
TCGA-MI-A75I-01A	61 years, male, black or african american, alive, 630 days	0.0
TCGA-DD-AACS-01A	39 years, male, asian, stage:i, alive, 1804 days	0.0
TCGA-CC-A3M9-01A	45 years, male, asian, stage:iiia, dead, 300 days	0.0
TCGA-YA-A8S7-01A	69 years, male, white, stage:iiia, dead, 412 days	0.0
TCGA-BC-A10U-01A	69 years, male, white, dead, 837 days	0.0
TCGA-CC-A7IE-01A	57 years, male, asian, stage:iiia, dead, 217 days	0.0
TCGA-LG-A9QC-01A	48 years, male, white, stage:i, alive, 425 days	0.0
TCGA-DD-A116-01A	68 years, male, asian, stage:iiia, dead, 1622 days	0.0
TCGA-DD-A73E-01A	66 years, male, white, stage:i, alive, 44 days	0.0
TCGA-2Y-A9H6-01A	68 years, female, white, stage:i, alive, 357 days	0.0
TCGA-BC-A3KG-01A	68 years, female, white, stage:ii, alive, 680 days	0.0
TCGA-DD-AAD3-01A	43 years, male, asian, stage:i, alive, 1295 days	0.0
TCGA-DD-A3A9-01A	64 years, female, white, stage:ivb, dead, 931 days	0.0
TCGA-DD-AADO-01A	55 years, male, asian, stage:i, alive, 453 days	0.0
TCGA-DD-AACD-01A	48 years, male, asian, stage:i, dead, 381 days	0.0
TCGA-G3-AAV3-01A	58 years, female, white, stage:ii, alive, 412 days	0.0
TCGA-NI-A8LF-01A	74 years, male, white, stage:i, alive, 799 days	0.0
TCGA-DD-AAEI-01A	72 years, male, asian, stage:i, alive, 1531 days	0.0
TCGA-FV-A2QQ-01A	80 years, male, white, stage:i, alive, 729 days	0.0
TCGA-FV-A3I1-01A	female, white, stage:ii, dead, 247 days	0.0
TCGA-DD-A11A-01A	67 years, male, black or african american, stage:i, alive, 79 days	0.0
TCGA-ZP-A9CV-01A	59 years, male, white, dead, 1088 days	0.0
TCGA-5R-AA1D-01A	17 years, female, white, stage:iiia, alive, 449 days	0.0
TCGA-CC-A7IF-01A	59 years, male, asian, stage:iiia, dead, 649 days	0.0

TCGA-DD-A73D-01A	68 years, female, white, stage:ii, alive, 693 days	0.0
TCGA-RG-A7D4-01A	69 years, male, black or african american, stage:ii, alive, 1098 days	0.0
TCGA-RC-A7SK-01A	59 years, male, asian, stage:i, alive, 472 days	0.0
TCGA-DD-A1EF-01A	57 years, female, white, stage:i, dead, 394 days	0.0
TCGA-DD-A73A-01A	71 years, male, white, stage:i, alive, 728 days	0.0
TCGA-2Y-A9GU-01A	55 years, female, white, stage:i, alive, 1939 days	0.0
TCGA-WQ-AB4B-01A	62 years, male, white, stage:ii, alive, 395 days	0.0
TCGA-CC-A5UD-01A	45 years, male, asian, stage:iiia, dead, 304 days	0.0
TCGA-DD-A3A3-01A	45 years, male, asian, stage:i, dead, 535 days	0.0
TCGA-WJ-A86L-01A	68 years, female, white, stage:i, alive, 345 days	0.0
TCGA-ZS-A9CE-01A	79 years, female, white, stage:ii, alive, 1241 days	0.0
TCGA-2Y-A9H4-01A	68 years, male, black or african american, stage:i, alive, 1452 days	0.0
TCGA-DD-AAEA-01A	65 years, male, asian, stage:i, alive, 575 days	0.0
TCGA-EP-A3JL-01A	76 years, male, white, stage:i, alive, 303 days	0.0
TCGA-DD-A73G-01A	73 years, female, white, stage:i, alive, 3478 days	0.0
TCGA-DD-AADA-01A	66 years, female, asian, stage:i, alive, 1233 days	0.0
TCGA-G3-AAV2-01A	50 years, male, white, stage:i, alive, 372 days	0.0
TCGA-QA-A7B7-01A	48 years, male, black or african american, stage:ii, alive, 94 days	0.0
TCGA-DD-AACT-01A	69 years, female, asian, stage:i, alive, 1562 days	0.0
TCGA-G3-AAV1-01A	51 years, male, asian, stage:iiic, dead, 359 days	0.0
TCGA-DD-A39Z-01A	43 years, female, stage:ii, dead, 601 days	0.0
TCGA-DD-A1EK-01A	64 years, female, white, stage:ivb, dead, 558 days	0.0
TCGA-BC-A216-01A	62 years, female, white, stage:iiia, alive, 1351 days	0.0
TCGA-LG-A6GG-01A	79 years, female, white, stage:ii, alive, 387 days	0.0
TCGA-DD-AAVW-01A	35 years, male, asian, stage:i, alive, 2317 days	0.0
TCGA-BC-A3KF-01A	66 years, female, white, stage:i, alive, 8 days	0.0
TCGA-CC-A8HV-01A	51 years, female, asian, stage:ii, dead, 279 days	0.0
TCGA-O8-A75V-01A	54 years, male, stage:i, alive, 538 days	0.0
TCGA-G3-A7M7-01A	65 years, male, white, stage:i, alive, 361 days	0.0
TCGA-PD-A5DF-01A	58 years, female, white, stage:iiib, dead, 639 days	0.0
TCGA-DD-AACZ-01A	63 years, female, asian, stage:i, dead, 171 days	0.0
TCGA-5C-A9VG-01A	58 years, male, white, stage:ii, alive, 328 days	0.0
TCGA-2Y-A9HA-01A	70 years, male, white, stage:ii, dead, 36 days	0.0
TCGA-BD-A2L6-01A	69 years, male, white, alive, 1363 days	0.0
TCGA-DD-AAD5-01A	54 years, male, asian, stage:i, alive, 1345 days	0.0
TCGA-DD-AACF-01A	68 years, male, asian, stage:i, dead, 365 days	0.0
TCGA-DD-A4NR-01A	85 years, female, white, stage:i, dead, 9 days	0.0
TCGA-UB-A7MB-01A	24 years, male, white, stage:ii, alive, 601 days	0.0
TCGA-2Y-A9GV-01A	54 years, female, white, stage:i, dead, 2532 days	0.0
TCGA-BC-A217-01A	75 years, female, white, stage:ii, dead, 1397 days	0.0
TCGA-EP-A2KC-01A	62 years, male, black or african american, stage:i, dead, 19 days	0.0
TCGA-DD-A73B-01A	72 years, female, white, stage:i, dead, 283 days	0.0
TCGA-RC-A7SH-01A	42 years, male, asian, stage:ii, alive, 468 days	0.0

TCGA-DD-A1EJ-01A	71 years, female, white, stage:iiic, dead, 1005 days	0.0
TCGA-G3-A3CG-01A	80 years, male, white, stage:i, alive, 673 days	0.0
TCGA-DD-A115-01A	53 years, male, white, stage:iiia, dead, 2542 days	0.0
TCGA-KR-A7K8-01A	57 years, male, stage:i, alive, 906 days	0.0
TCGA-DD-AAEH-01A	73 years, male, asian, stage:i, alive, 784 days	0.0
TCGA-DD-A1EE-01A	73 years, male, white, stage:iiia, dead, 349 days	0.0
TCGA-5R-AAAM-01A	65 years, female, white, stage:ii, dead, 46 days	0.0
TCGA-WX-AA46-01A	62 years, male, white, stage:ii, alive, 756 days	0.0
TCGA-DD-A3A5-01A	66 years, female, white, stage:iii, dead, 3125 days	0.0
TCGA-MR-A8JO-01A	34 years, male, white, stage:i, alive, 330 days	0.0
TCGA-G3-A25S-01A	64 years, male, white, stage:i, dead, 416 days	0.0
TCGA-BC-A69I-01A	69 years, male, white, stage:i, alive, 387 days	0.0
TCGA-DD-AADB-01A	51 years, male, asian, stage:i, alive, 1242 days	0.0
TCGA-DD-A3A4-01A	37 years, male, white, stage:iiia, dead, 612 days	0.0
TCGA-EP-A12J-01A	63 years, male, black or african american, stage:i, alive, 570 days	0.0
TCGA-FV-A4ZQ-01A	52 years, male, white, stage:i, alive, 12 days	0.0
TCGA-G3-A7M5-01A	76 years, male, asian, stage:i, alive, 447 days	0.0
TCGA-CC-A9FS-01A	55 years, male, asian, stage:ii, alive, 211 days	0.0
TCGA-DD-AAE9-01A	69 years, male, asian, stage:i, alive, 722 days	0.0
TCGA-ZP-A9D4-01A	64 years, female, white, alive, 395 days	0.0
TCGA-ZP-A9D0-01A	67 years, female, black or african american, alive, 1091 days	0.0
TCGA-DD-A39W-01A	29 years, female, white, stage:iii, dead, 827 days	0.0
TCGA-DD-AAEK-01A	51 years, male, asian, stage:ii, alive, 1067 days	0.0
TCGA-DD-AAVS-01A	56 years, male, asian, stage:i, alive, 1823 days	0.0
TCGA-CC-A7IH-01A	58 years, male, asian, stage:iiia, alive, 365 days	0.0
TCGA-DD-AACK-01A	70 years, male, asian, stage:i, alive, 9 days	0.0
TCGA-2Y-A9H7-01A	81 years, female, white, stage:i, alive, 1168 days	0.0
TCGA-BC-A10Y-01A	76 years, male, white, dead, 711 days	0.0
TCGA-DD-AAW2-01A	69 years, male, asian, stage:i, alive, 1855 days	0.0
TCGA-RC-A6M4-01A	74 years, female, white, stage:iiia, alive, 22 days	0.0
TCGA-DD-A4NJ-01A	54 years, female, white, stage:ii, alive, 928 days	0.0
TCGA-ED-A7PY-01A	20 years, female, asian, stage:ii, alive, 390 days	0.0
TCGA-CC-A7IL-01A	61 years, male, asian, stage:iiia, dead, 278 days	0.0
TCGA-UB-A7MF-01A	57 years, male, white, stage:iiia, dead, 214 days	0.0
TCGA-EP-A26S-01A	70 years, male, white, stage:i, alive, 608 days	0.0
TCGA-DD-AADJ-01A	70 years, female, asian, stage:i, alive, 1066 days	0.0
TCGA-DD-AAW3-01A	69 years, male, asian, stage:i, alive, 1633 days	0.0
TCGA-DD-AAE1-01A	52 years, male, asian, stage:i, alive, 552 days	0.0
TCGA-DD-AAE4-01A	49 years, female, asian, stage:i, alive, 608 days	0.0
TCGA-DD-AACV-01A	53 years, male, asian, stage:i, alive, 1531 days	0.0
TCGA-DD-AADS-01A	63 years, male, asian, stage:i, alive, 474 days	0.0
TCGA-DD-A39V-01A	77 years, male, white, stage:ii, dead, 643 days	0.0
TCGA-DD-A4NP-01A	32 years, male, white, stage:i, alive, 3308 days	0.0

TCGA-DD-AADD-01A	51 years, male, asian, stage:i, alive, 1231 days	0.0
TCGA-G3-A5SI-01A	44 years, male, asian, stage:ii, dead, 768 days	0.0
TCGA-WX-AA47-01A	33 years, female, white, stage:iiia, dead, 556 days	0.0
TCGA-RC-A7SF-01A	66 years, male, asian, stage:i, alive, 579 days	0.0
TCGA-G3-AAV0-01A	58 years, male, asian, stage:i, alive, 476 days	0.0
TCGA-G3-A5SK-01A	58 years, male, white, stage:i, alive, 744 days	0.0
TCGA-G3-AAUZ-01A	48 years, male, stage:i, alive, 480 days	0.0
TCGA-CC-A8HU-01A	39 years, female, asian, stage:iiia, dead, 344 days	0.0
TCGA-DD-AADU-01A	60 years, male, asian, stage:ii, alive, 554 days	0.0
TCGA-DD-AAVZ-01A	38 years, male, asian, stage:i, alive, 1900 days	0.0
TCGA-DD-A4NL-01A	46 years, male, white, stage:i, alive, 1711 days	0.0
TCGA-DD-A4NK-01A	80 years, female, white, stage:iiia, dead, 1210 days	0.0
TCGA-XR-A8TF-01A	74 years, male, white, stage:i, dead, 693 days	0.0
TCGA-DD-AAE6-01A	59 years, female, asian, stage:i, alive, 141 days	0.0
TCGA-2Y-A9GY-01A	64 years, female, white, stage:ii, dead, 757 days	0.0
TCGA-ZP-A9CZ-01A	72 years, male, asian, alive, 706 days	0.0
TCGA-HP-A5N0-01A	88 years, female, dead, 1147 days	0.0
TCGA-DD-AACX-01A	66 years, male, asian, stage:ii, alive, 170 days	0.0
TCGA-2Y-A9H8-01A	85 years, female, white, dead, 633 days	0.0
TCGA-ZS-A9CG-01A	55 years, male, white, stage:ii, alive, 341 days	0.0
TCGA-FV-A2QR-01A	male, white, stage:i, dead, 581 days	0.0
TCGA-DD-A3A1-01A	65 years, male, stage:iiia, dead, 233 days	0.0
TCGA-DD-AADG-01A	70 years, male, asian, stage:iiia, alive, 1145 days	0.0
TCGA-G3-AAV6-01A	53 years, female, white, stage:iiia, dead, 65 days	0.0
TCGA-DD-A3A8-01A	75 years, male, white, stage:ii, dead, 11 days	0.0
TCGA-KR-A7K7-01A	61 years, female, white, stage:ii, alive, 951 days	0.0
TCGA-XR-A8TE-01A	16 years, male, white, stage:iiia, alive, 925 days	0.0
TCGA-CC-A7IK-01A	59 years, male, asian, stage:iiia, dead, 262 days	0.0
TCGA-DD-A3A2-01A	76 years, female, white, stage:i, dead, 2131 days	0.0
TCGA-DD-AAEE-01A	55 years, male, asian, stage:i, alive, 810 days	0.0
TCGA-LG-A9QD-01A	68 years, male, white, stage:iiia, alive, 366 days	0.0
TCGA-CC-A9FW-01A	68 years, male, asian, stage:iiia, alive, 248 days	0.0
TCGA-DD-AAE2-01A	51 years, male, asian, stage:i, alive, 638 days	0.0
TCGA-G3-AAV5-01A	67 years, male, white, stage:ii, alive, 354 days	0.0
TCGA-G3-A5SL-01A	70 years, male, white, stage:ii, alive, 621 days	0.0
TCGA-BC-A10S-01A	81 years, male, white, dead, 1423 days	0.0
TCGA-ES-A2HT-01A	54 years, male, black or african american, stage:i, dead, 438 days	0.0
TCGA-2Y-A9H9-01A	70 years, male, white, stage:i, alive, 697 days	0.0
TCGA-DD-A1EG-01A	77 years, male, white, stage:i, dead, 1372 days	0.0
TCGA-EP-A2KB-01A	46 years, female, white, stage:i, dead, 596 days	0.0
TCGA-CC-5258-01A	48 years, male, asian, stage:ii, dead, 129 days	0.0
TCGA-DD-A1EH-01A	23 years, male, white, stage:iii, alive, 1495 days	0.0
TCGA-FV-A23B-01A	70 years, female, white, stage:ii, dead, 1852 days	0.0

TCGA-G3-A25Z-01A	58 years, male, asian, stage:i, alive, 655 days	0.0
TCGA-NI-A4U2-01A	71 years, male, white, stage:iiia, dead, 1791 days	0.0
TCGA-DD-AAD0-01A	73 years, female, asian, stage:i, alive, 137 days	0.0
TCGA-DD-AAVX-01A	38 years, male, asian, stage:ii, alive, 1570 days	0.0
TCGA-DD-AADN-01A	59 years, male, asian, stage:i, alive, 898 days	0.0
TCGA-DD-A4NN-01A	56 years, female, white, stage:i, dead, 899 days	0.0
TCGA-DD-A3A7-01A	67 years, male, stage:iiib, dead, 419 days	0.0
TCGA-DD-A11D-01A	57 years, female, white, stage:i, dead, 1560 days	0.0
TCGA-2Y-A9H3-01A	45 years, male, white, stage:ii, alive, 1516 days	0.0
TCGA-DD-A4NF-01A	72 years, male, white, stage:i, alive, 942 days	0.0
TCGA-DD-A4NB-01A	25 years, male, white, stage:i, alive, 989 days	0.0
TCGA-DD-AADF-01A	64 years, female, asian, stage:i, dead, 115 days	0.0
TCGA-DD-AACY-01A	61 years, male, asian, stage:i, alive, 1450 days	0.0
TCGA-3K-AAZ8-01A	65 years, male, black or african american, stage:iiib, alive, 396 days	0.0
TCGA-DD-AADQ-01A	59 years, male, asian, stage:ii, alive, 436 days	0.0
TCGA-BC-A10Z-01A	62 years, female, white, stage:i, dead, 34 days	0.0
TCGA-DD-AAC8-01A	72 years, male, asian, stage:i, dead, 16 days	0.0
TCGA-DD-AAW1-01A	55 years, male, asian, stage:iiia, alive, 1989 days	0.0
TCGA-2Y-A9GZ-01A	82 years, female, white, stage:ii, dead, 848 days	0.0
TCGA-G3-A25U-01A	63 years, female, asian, stage:i, alive, 1636 days	0.0
TCGA-G3-AAV4-01A	83 years, female, white, stage:i, dead, 27 days	0.0
TCGA-ED-A7XP-01A	53 years, female, asian, stage:ii, alive, 400 days	0.0
TCGA-CC-A123-01A	24 years, female, asian, stage:iiia, alive, 219 days	0.0
TCGA-BW-A5NO-01A	50 years, male, black or african american, stage:iiia, alive, 20 days	0.0
TCGA-DD-A119-01A	40 years, male, asian, stage:iv, dead, 223 days	0.0
TCGA-DD-A4NI-01A	67 years, male, white, stage:ii, alive, 816 days	0.0
TCGA-DD-A4NG-01A	77 years, male, white, stage:iiia, dead, 802 days	0.0
TCGA-2Y-A9HB-01A	66 years, male, stage:i, alive, 260 days	0.0
TCGA-FV-A3R2-01A	75 years, male, white, stage:i, dead, 194 days	0.0
TCGA-MI-A75H-01A	77 years, male, white, alive, 747 days	0.0
TCGA-DD-A73C-01A	65 years, female, white, stage:iiia, alive, 701 days	0.0
TCGA-ZS-A9CD-01A	73 years, male, white, stage:ii, dead, 1386 days	0.0
TCGA-DD-AAD8-01A	73 years, female, asian, stage:i, alive, 1219 days	0.0
TCGA-G3-A3CJ-01A	52 years, male, american indian or alaska native, stage:ii, alive, 594 days	0.0
TCGA-DD-A1EL-01A	23 years, male, black or african american, stage:ii, dead, 415 days	0.0
TCGA-DD-A11C-01A	69 years, male, white, stage:i, alive, 662 days	0.0
TCGA-2Y-A9H1-01A	58 years, male, white, stage:i, dead, 1229 days	0.0
TCGA-G3-A6UC-01A	65 years, male, white, stage:iiib, alive, 671 days	0.0
TCGA-DD-AACB-01A	74 years, female, asian, stage:i, alive, 2324 days	0.0
TCGA-DD-AAD6-01A	66 years, male, asian, stage:iiia, alive, 672 days	0.0
TCGA-DD-AACA-01A	65 years, male, asian, stage:i, alive, 2301 days	0.0
TCGA-DD-AADM-01A	58 years, male, asian, stage:ii, dead, 12 days	0.0
TCGA-DD-AAVU-01A	46 years, male, asian, stage:ii, alive, 2202 days	0.0

TCGA-DD-A4NO-01A	66 years, male, white, stage:i, alive, 2245 days	0.0
TCGA-DD-A1EA-01A	68 years, male, asian, stage:ii, alive, 2415 days	0.0
TCGA-DD-AACE-01A	62 years, male, asian, stage:i, alive, 2184 days	0.0
TCGA-ES-A2HS-01A	80 years, male, white, stage:i, dead, 688 days	0.0
TCGA-DD-A11B-01A	73 years, male, white, stage:i, dead, 14 days	0.0
TCGA-DD-AACJ-01A	75 years, male, asian, stage:ii, alive, 2102 days	0.0
TCGA-ZS-A9CF-01A	64 years, male, white, stage:ii, alive, 2412 days	0.0
TCGA-DD-A113-01A	55 years, female, white, stage:ii, alive, 2425 days	0.0
TCGA-DD-AAVY-01A	56 years, male, asian, stage:iiia, alive, 1970 days	0.0
TCGA-DD-A1EB-01A	72 years, female, stage:i, alive, 2017 days	0.0
TCGA-FV-A496-01A	84 years, female, white, stage:i, alive, 10 days	0.0
TCGA-FV-A4ZP-01A	78 years, male, white, stage:iiia, dead, 2486 days	0.0
TCGA-5C-A9VH-01A	70 years, male, white, stage:i, alive, 322 days	0.0
TCGA-G3-A3CK-01A	61 years, male, asian, stage:i, alive, 585 days	0.0
TCGA-DD-AACP-01A	65 years, male, asian, stage:i, alive, 415 days	0.0
TCGA-DD-AADV-01A	50 years, male, asian, stage:i, alive, 574 days	0.0
TCGA-DD-AAVP-01A	48 years, male, asian, stage:i, alive, 2752 days	0.0
TCGA-DD-AAE3-01A	50 years, male, asian, stage:i, alive, 566 days	0.0
TCGA-EP-A3RK-01A	73 years, male, white, stage:iiia, alive, 363 days	0.0
TCGA-5R-AA1C-01A	57 years, male, white, stage:ii, alive, 520 days	0.0
TCGA-DD-A118-01A	77 years, female, white, stage:ii, alive, 3437 days	0.0
TCGA-UB-A7MC-01A	59 years, male, white, stage:iiia, alive, 500 days	0.0
TCGA-DD-AADR-01A	58 years, male, asian, stage:i, alive, 2028 days	0.0
TCGA-DD-AAEB-01A	60 years, male, asian, stage:i, alive, 478 days	0.0

Supplementary Table 2 Clinical traits of hepatocellular carcinoma patients who express *TRPV2* or *TRPV4*.

Index	TRPV2 (people)	TRPV4 (people)
Age	Below 50: 68 ;<br>50-60: 97 ;<br>60-70: 118 ;<br>Above 70: 79 ;	Below 50: 68 ;<br>50-60: 97 ;<br>60-70: 118 ;<br>Above 70: 79 ;
Sex	Female: 119 ;<br>Male: 246 ;	Female: 117 ;<br>Male: 248 ;
Area	Asian: 155 ;<br>White: 182 ;<br>Black: 17 ;	Asian: 155 ;<br>White: 182 ;<br>Black: 17 ;
Clinical stages	Stage I: 170<br>Stage II: 84<br>Stage IIIa: 63<br>Stage IIIb: 8<br>Stage IIIc: 9<br>Stage IV: 1<br>Stage IVa: 1<br>Stage IVb: 2	Stage I: 170<br>Stage II: 84<br>Stage IIIa: 63<br>Stage IIIb: 8<br>Stage IIIc: 9<br>Stage IV: 1<br>Stage IVa: 1<br>Stage IVb: 2
Survival	Dead: 130 ;<br>Alive: 235 ;	Dead: 130 ;<br>Alive: 235 ;
Prior Malignancy	Yes: a few<br>No: majority	Yes: a few<br>No: majority
Progress/recurrence	Not reported	Not reported
Family history	Not reported	Not reported
Alcohol/smoking	Not reported	Not reported

Supplementary Table 3 Non-stage I HCC cases with *TRPV2* or *TRPV4* RNA low and high expression from TCGA databases

Non-stage I HCC cases with *TRPV2* RNA low and high expression (n=195)

Sample	Description	FPKM
TCGA-RC-A6M6-01A	75 years, male, white, stage:ii, alive, 9 days	37.8
TCGA-2Y-A9GY-01A	64 years, female, white, stage:ii, dead, 757 days	29.9
TCGA-FV-A4ZP-01A	78 years, male, white, stage:iiia, dead, 2486 days	21.9
TCGA-DD-A3A6-01A	72 years, female, white, stage:ii, dead, 3258 days	21.8
TCGA-DD-AACH-01A	69 years, male, asian, stage:ii, dead, 195 days	21.2
TCGA-CC-A7IL-01A	61 years, male, asian, stage:iiia, dead, 278 days	20.8
TCGA-BC-4073-01B	73 years, male, white, stage:iiia, alive, 849 days	19.0
TCGA-DD-A113-01A	55 years, female, white, stage:ii, alive, 2425 days	16.1
TCGA-CC-A7IE-01A	57 years, male, asian, stage:iiia, dead, 217 days	15.7
TCGA-CC-A3M9-01A	45 years, male, asian, stage:iiia, dead, 300 days	15.4
TCGA-CC-A5UE-01A	48 years, male, asian, stage:iiib, dead, 272 days	11.4
TCGA-ED-A5KG-01A	60 years, female, asian, stage:ii, alive, 854 days	11.2
TCGA-CC-A8HT-01A	74 years, male, asian, stage:iiia, dead, 140 days	10.7
TCGA-5C-AAPD-01A	61 years, male, asian, stage:ii, alive, 20 days	8.2
TCGA-DD-A114-01A	42 years, male, black or african american, stage:ii, dead, 1149 days	7.9
TCGA-CC-A1HT-01A	50 years, male, asian, stage:iiia, dead, 101 days	7.7
TCGA-EP-A3RK-01A	73 years, male, white, stage:iiia, alive, 363 days	7.5
TCGA-CC-A3MA-01A	61 years, male, asian, stage:iiia, dead, 303 days	6.7
TCGA-DD-AACI-01A	69 years, male, asian, stage:ii, alive, 1618 days	6.6
TCGA-XR-A8TD-01A	49 years, female, white, stage:iiib, alive, 1030 days	6.0
TCGA-G3-A7M9-01A	70 years, male, white, stage:iiib, dead, 56 days	6.0
TCGA-CC-5260-01A	61 years, female, asian, stage:iiic, dead, 87 days	5.7
TCGA-YA-A8S7-01A	69 years, male, white, stage:iiia, dead, 412 days	5.5
TCGA-CC-A7IJ-01A	56 years, male, asian, stage:ii, alive, 382 days	5.4
TCGA-G3-A25X-01A	73 years, male, asian, stage:ii, alive, 1779 days	5.1
TCGA-2Y-A9H3-01A	45 years, male, white, stage:ii, alive, 1516 days	5.0
TCGA-CC-5261-01A	44 years, male, asian, stage:ii, dead, 97 days	4.8
TCGA-ED-A66X-01A	35 years, male, asian, stage:iiia, alive, 406 days	4.6
TCGA-FV-A495-01A	51 years, female, white, stage:ii, alive, 1 days	4.5
TCGA-GJ-A6C0-01A	75 years, female, white, stage:ii, dead, 31 days	4.3
TCGA-2Y-A9HA-01A	70 years, male, white, stage:ii, dead, 36 days	4.3
TCGA-ED-A7PX-01A	48 years, female, asian, stage:ii, alive, 6 days	4.1
TCGA-2Y-A9H0-01A	49 years, male, white, stage:iiia, alive, 3675 days	4.1
TCGA-DD-A4NA-01A	67 years, female, white, stage:iiic, alive, 1008 days	4.1
TCGA-5R-AA1D-01A	17 years, female, white, stage:iiia, alive, 449 days	4.0
TCGA-DD-AAVV-01A	56 years, male, asian, stage:ii, alive, 2455 days	3.9
TCGA-PD-A5DF-01A	58 years, female, white, stage:iiib, dead, 639 days	3.8

TCGA-G3-AAV3-01A	58 years, female, white, stage:ii, alive, 412 days	3.8
TCGA-DD-A3A9-01A	64 years, female, white, stage:ivb, dead, 931 days	3.7
TCGA-CC-5262-01A	67 years, male, asian, stage:iiic, dead, 103 days	3.6
TCGA-UB-A7MF-01A	57 years, male, white, stage:iiia, dead, 214 days	3.6
TCGA-ED-A8O6-01A	50 years, female, asian, stage:iiia, dead, 56 days	3.5
TCGA-ED-A97K-01A	54 years, male, asian, stage:iiia, alive, 6 days	3.5
TCGA-BC-A10S-01A	81 years, male, white, dead, 1423 days	3.4
TCGA-BC-4072-01B	74 years, female, white, stage:iiia, dead, 1490 days	3.4
TCGA-DD-A39V-01A	77 years, male, white, stage:ii, dead, 643 days	3.4
TCGA-CC-A5UC-01A	63 years, male, asian, stage:iiia, dead, 347 days	3.2
TCGA-BC-A69H-01A	64 years, male, white, stage:ii, alive, 444 days	3.1
TCGA-DD-AAEK-01A	51 years, male, asian, stage:ii, alive, 1067 days	3.1
TCGA-FV-A3I1-01A	female, white, stage:ii, dead, 247 days	3.0
TCGA-G3-A25T-01A	45 years, female, white, stage:iiia, alive, 1553 days	3.0
TCGA-5R-AAAM-01A	65 years, female, white, stage:ii, dead, 46 days	3.0
TCGA-ZP-A9CZ-01A	72 years, male, asian, alive, 706 days	3.0
TCGA-K7-AAU7-01A	61 years, male, white, stage:ii, alive, 359 days	2.9
TCGA-BC-A112-01A	80 years, male, white, dead, 153 days	2.9
TCGA-ED-A8O5-01A	59 years, female, asian, stage:iiia, alive, 406 days	2.9
TCGA-ED-A82E-01A	60 years, female, asian, stage:iiia, alive, 408 days	2.8
TCGA-G3-AAV7-01A	38 years, male, asian, stage:ii, alive, 361 days	2.8
TCGA-BC-A110-01A	51 years, female, black or african american, dead, 2116 days	2.8
TCGA-DD-A1EL-01A	23 years, male, black or african american, stage:ii, dead, 415 days	2.8
TCGA-BC-A8YO-01A	66 years, female, white, stage:iiic, alive, 562 days	2.8
TCGA-CC-5263-01A	35 years, male, asian, stage:iiia, dead, 129 days	2.7
TCGA-BC-A10T-01A	76 years, male, white, dead, 837 days	2.7
TCGA-ED-A4XI-01A	58 years, male, asian, stage:ii, alive, 819 days	2.6
TCGA-G3-A5SM-01A	58 years, male, white, stage:ii, alive, 520 days	2.6
TCGA-RG-A7D4-01A	69 years, male, black or african american, stage:ii, alive, 1098 days	2.6
TCGA-DD-AAE0-01A	45 years, female, asian, stage:iiia, alive, 555 days	2.6
TCGA-CC-A7II-01A	55 years, male, asian, stage:iiia, alive, 399 days	2.5
TCGA-CC-5258-01A	48 years, male, asian, stage:ii, dead, 129 days	2.5
TCGA-ED-A7PZ-01A	61 years, male, asian, stage:ii, alive, 6 days	2.5
TCGA-DD-A4NJ-01A	54 years, female, white, stage:ii, alive, 928 days	2.5
TCGA-RC-A6M5-01A	20 years, female, white, stage:iva, alive, 15 days	2.4
TCGA-DD-A1EK-01A	64 years, female, white, stage:ivb, dead, 558 days	2.4
TCGA-UB-A7MA-01A	62 years, female, white, stage:ii, alive, 848 days	2.4
TCGA-HP-A5N0-01A	88 years, female, dead, 1147 days	2.3
TCGA-2Y-A9H8-01A	85 years, female, white, dead, 633 days	2.3
TCGA-KR-A7K7-01A	61 years, female, white, stage:ii, alive, 951 days	2.3
TCGA-BC-A10W-01A	50 years, male, asian, dead, 91 days	2.3
TCGA-FV-A3I0-01A	76 years, female, white, stage:ii, alive, 848 days	2.2
TCGA-DD-A4NV-01A	61 years, male, white, stage:iiia, alive, 2398 days	2.2

TCGA-BC-A10Y-01A	76 years, male, white, dead, 711 days	2.2
TCGA-DD-A4NH-01A	65 years, female, white, stage:iiib, alive, 917 days	2.2
TCGA-ED-A7XO-01A	29 years, male, asian, stage:iiia, alive, 427 days	2.2
TCGA-DD-A119-01A	40 years, male, asian, stage:iv, dead, 223 days	2.1
TCGA-ED-A7XP-01A	53 years, female, asian, stage:ii, alive, 400 days	2.1
TCGA-DD-AACG-01A	52 years, male, asian, stage:ii, dead, 469 days	2.1
TCGA-5C-A9VG-01A	58 years, male, white, stage:ii, alive, 328 days	2.1
TCGA-BD-A3ER-01A	62 years, male, white, stage:ii, alive, 1115 days	2.1
TCGA-EP-A2KA-01A	52 years, female, white, stage:iiia, dead, 627 days	2.1
TCGA-ZS-A9CD-01A	73 years, male, white, stage:ii, dead, 1386 days	2.1
TCGA-UB-AA0U-01A	60 years, male, white, stage:ii, alive, 327 days	2.1
TCGA-BC-A216-01A	62 years, female, white, stage:iiia, alive, 1351 days	2.0
TCGA-ZP-A9D1-01A	56 years, female, white, alive, 21 days	2.0
TCGA-MI-A75H-01A	77 years, male, white, alive, 747 days	2.0
TCGA-CC-A7IG-01A	47 years, male, asian, stage:ii, dead, 299 days	2.0
TCGA-DD-A1EJ-01A	71 years, female, white, stage:iiic, dead, 1005 days	2.0
TCGA-BC-A10X-01A	52 years, female, white, stage:iiia, dead, 770 days	1.9
TCGA-DD-AADK-01A	68 years, female, asian, stage:ii, alive, 1049 days	1.9
TCGA-2Y-A9GS-01A	58 years, male, white, dead, 724 days	1.9
TCGA-FV-A23B-01A	70 years, female, white, stage:ii, dead, 1852 days	1.9
TCGA-DD-A118-01A	77 years, female, white, stage:ii, alive, 3437 days	1.9
TCGA-CC-A5UD-01A	45 years, male, asian, stage:iiia, dead, 304 days	1.9
TCGA-DD-AAVX-01A	38 years, male, asian, stage:ii, alive, 1570 days	1.8
TCGA-DD-A115-01A	53 years, male, white, stage:iiia, dead, 2542 days	1.8
TCGA-DD-A3A5-01A	66 years, female, white, stage:iii, dead, 3125 days	1.8
TCGA-G3-A3CH-01A	53 years, male, asian, stage:iiia, alive, 780 days	1.8
TCGA-BD-A2L6-01A	69 years, male, white, alive, 1363 days	1.8
TCGA-CC-A9FW-01A	68 years, male, asian, stage:iiia, alive, 248 days	1.7
TCGA-DD-A1EA-01A	68 years, male, asian, stage:ii, alive, 2415 days	1.7
TCGA-ZS-A9CF-01A	64 years, male, white, stage:ii, alive, 2412 days	1.7
TCGA-CC-A3MC-01A	54 years, male, asian, stage:iiia, alive, 363 days	1.7
TCGA-DD-A116-01A	68 years, male, asian, stage:iiia, dead, 1622 days	1.7
TCGA-DD-AACJ-01A	75 years, male, asian, stage:ii, alive, 2102 days	1.7
TCGA-ZP-A9D2-01A	51 years, male, white, dead, 765 days	1.7
TCGA-4R-AA8I-01A	66 years, male, white, stage:ii, dead, 262 days	1.7
TCGA-BC-A10Q-01A	72 years, female, white, dead, 1135 days	1.7
TCGA-BW-A5NO-01A	50 years, male, black or african american, stage:iiia, alive, 20 days	1.6
TCGA-DD-AAVU-01A	46 years, male, asian, stage:ii, alive, 2202 days	1.6
TCGA-DD-A4NI-01A	67 years, male, white, stage:ii, alive, 816 days	1.6
TCGA-WQ-AB4B-01A	62 years, male, white, stage:ii, alive, 395 days	1.6
TCGA-MI-A75E-01A	61 years, male, white, stage:iiic, alive, 507 days	1.6
TCGA-CC-5259-01A	60 years, female, asian, stage:iiic, alive, 250 days	1.6
TCGA-ZP-A9CY-01A	66 years, female, white, alive, 782 days	1.6

TCGA-BC-A10R-01A	66 years, female, white, dead, 308 days	1.6
TCGA-CC-A3MB-01A	36 years, male, asian, stage:iiia, dead, 315 days	1.6
TCGA-BC-A217-01A	75 years, female, white, stage:ii, dead, 1397 days	1.5
TCGA-G3-AAV1-01A	51 years, male, asian, stage:iiic, dead, 359 days	1.5
TCGA-ZP-A9CV-01A	59 years, male, white, dead, 1088 days	1.5
TCGA-CC-A8HS-01A	18 years, male, asian, stage:iiic, dead, 300 days	1.5
TCGA-DD-A4NG-01A	77 years, male, white, stage:iiia, dead, 802 days	1.5
TCGA-DD-A3A7-01A	67 years, male, stage:iiib, dead, 419 days	1.4
TCGA-CC-A123-01A	24 years, female, asian, stage:iiia, alive, 219 days	1.4
TCGA-DD-A3A1-01A	65 years, male, stage:iiia, dead, 233 days	1.4
TCGA-G3-AAV6-01A	53 years, female, white, stage:iiia, dead, 65 days	1.4
TCGA-CC-A7IH-01A	58 years, male, asian, stage:iiia, alive, 365 days	1.4
TCGA-DD-A73C-01A	65 years, female, white, stage:iiia, alive, 701 days	1.4
TCGA-NI-A4U2-01A	71 years, male, white, stage:iiia, dead, 1791 days	1.3
TCGA-DD-AADM-01A	58 years, male, asian, stage:ii, dead, 12 days	1.3
TCGA-DD-A4NK-01A	80 years, female, white, stage:iiia, dead, 1210 days	1.3
TCGA-DD-A1EE-01A	73 years, male, white, stage:iiia, dead, 349 days	1.3
TCGA-RC-A7SH-01A	42 years, male, asian, stage:ii, alive, 468 days	1.3
TCGA-DD-A4NQ-01A	60 years, male, white, stage:ii, dead, 373 days	1.3
TCGA-DD-A1EH-01A	23 years, male, white, stage:iii, alive, 1495 days	1.2
TCGA-3K-AAZ8-01A	65 years, male, black or african american, stage:iiib, alive, 396 days	1.2
TCGA-CC-5264-01A	71 years, male, asian, stage:iiia, dead, 102 days	1.2
TCGA-WX-AA46-01A	62 years, male, white, stage:ii, alive, 756 days	1.2
TCGA-2Y-A9GZ-01A	82 years, female, white, stage:ii, dead, 848 days	1.2
TCGA-G3-AAV5-01A	67 years, male, white, stage:ii, alive, 354 days	1.1
TCGA-G3-A3CJ-01A	52 years, male, american indian or alaska native, stage:ii, alive, 594 days	1.1
TCGA-DD-AADG-01A	70 years, male, asian, stage:iiia, alive, 1145 days	1.1
TCGA-QA-A7B7-01A	48 years, male, black or african american, stage:ii, alive, 94 days	1.1
TCGA-WQ-A9G7-01A	female, white, alive, 30 days	1.1
TCGA-DD-AADQ-01A	59 years, male, asian, stage:ii, alive, 436 days	1.1
TCGA-CC-A9FS-01A	55 years, male, asian, stage:ii, alive, 211 days	1.1
TCGA-CC-A7IK-01A	59 years, male, asian, stage:iiia, dead, 262 days	1.1
TCGA-DD-A39W-01A	29 years, female, white, stage:iii, dead, 827 days	1.0
TCGA-ED-A459-01A	47 years, male, asian, stage:ii, alive, 910 days	1.0
TCGA-DD-AACX-01A	66 years, male, asian, stage:ii, alive, 170 days	1.0
TCGA-DD-AAVY-01A	56 years, male, asian, stage:iiia, alive, 1970 days	1.0
TCGA-5R-AA1C-01A	57 years, male, white, stage:ii, alive, 520 days	1.0
TCGA-DD-A4NE-01A	75 years, female, white, stage:iiia, dead, 660 days	1.0
TCGA-CC-A7IF-01A	59 years, male, asian, stage:iiia, dead, 649 days	0.9
TCGA-RC-A7SB-01A	53 years, male, asian, stage:ii, alive, 588 days	0.9
TCGA-UB-A7MB-01A	24 years, male, white, stage:ii, alive, 601 days	0.9
TCGA-T1-A6J8-01A	68 years, male, white, alive, 23 days	0.9

TCGA-MI-A75I-01A	61 years, male, black or african american, alive, 630 days	0.9
TCGA-LG-A9QD-01A	68 years, male, white, stage:iiia, alive, 366 days	0.9
TCGA-MI-A75G-01A	63 years, male, white, stage:ii, alive, 698 days	0.9
TCGA-DD-AAW1-01A	55 years, male, asian, stage:iiia, alive, 1989 days	0.9
TCGA-LG-A6GG-01A	79 years, female, white, stage:ii, alive, 387 days	0.9
TCGA-RC-A6M4-01A	74 years, female, white, stage:iiia, alive, 22 days	0.8
TCGA-BC-A3KG-01A	68 years, female, white, stage:ii, alive, 680 days	0.8
TCGA-DD-A73D-01A	68 years, female, white, stage:ii, alive, 693 days	0.8
TCGA-UB-A7MC-01A	59 years, male, white, stage:iiia, alive, 500 days	0.8
TCGA-DD-A39Z-01A	43 years, female, stage:ii, dead, 601 days	0.8
TCGA-ZP-A9D4-01A	64 years, female, white, alive, 395 days	0.8
TCGA-CC-A8HU-01A	39 years, female, asian, stage:iiia, dead, 344 days	0.8
TCGA-G3-A5SL-01A	70 years, male, white, stage:ii, alive, 621 days	0.8
TCGA-CC-A8HV-01A	51 years, female, asian, stage:ii, dead, 279 days	0.8
TCGA-ZS-A9CG-01A	55 years, male, white, stage:ii, alive, 341 days	0.8
TCGA-DD-AADU-01A	60 years, male, asian, stage:ii, alive, 554 days	0.7
TCGA-ZP-A9D0-01A	67 years, female, black or african american, alive, 1091 days	0.7
TCGA-BC-A10U-01A	69 years, male, white, dead, 837 days	0.7
TCGA-DD-AACQ-01A	50 years, male, asian, stage:ii, dead, 432 days	0.7
TCGA-ED-A66Y-01A	51 years, female, asian, stage:iiia, dead, 296 days	0.7
TCGA-G3-A6UC-01A	65 years, male, white, stage:iiib, alive, 671 days	0.6
TCGA-DD-A3A8-01A	75 years, male, white, stage:ii, dead, 11 days	0.6
TCGA-XR-A8TE-01A	16 years, male, white, stage:iiia, alive, 925 days	0.6
TCGA-G3-A5SI-01A	44 years, male, asian, stage:ii, dead, 768 days	0.6
TCGA-ED-A7PY-01A	20 years, female, asian, stage:ii, alive, 390 days	0.6
TCGA-DD-A3A4-01A	37 years, male, white, stage:iiia, dead, 612 days	0.6
TCGA-DD-AAD6-01A	66 years, male, asian, stage:iiia, alive, 672 days	0.5
TCGA-WX-AA47-01A	33 years, female, white, stage:iiia, dead, 556 days	0.4
TCGA-BC-A5W4-01A	69 years, male, white, stage:iiia, dead, 547 days	0.3
TCGA-ZS-A9CE-01A	79 years, female, white, stage:ii, alive, 1241 days	0.3

Non-stage I HCC cases with *TRPV4* RNA low and high expression (N=171)

Sample	Description	FPKM
TCGA-CC-A3MA-01A	61 years, male, asian, stage:iiia, dead, 303 days	32.7
TCGA-DD-A1EH-01A	23 years, male, white, stage:iii, alive, 1495 days	17.8
TCGA-ED-A7PX-01A	48 years, female, asian, stage:ii, alive, 6 days	14.4
TCGA-DD-A4NH-01A	65 years, female, white, stage:iiib, alive, 917 days	12.5
TCGA-DD-AAE0-01A	45 years, female, asian, stage:iiia, alive, 555 days	12.0
TCGA-CC-A8HS-01A	18 years, male, asian, stage:iiic, dead, 300 days	12.0
TCGA-BC-A5W4-01A	69 years, male, white, stage:iiia, dead, 547 days	11.7
TCGA-5C-AAPD-01A	61 years, male, asian, stage:ii, alive, 20 days	10.4
TCGA-ED-A82E-01A	60 years, female, asian, stage:iiia, alive, 408 days	7.8

TCGA-DD-AADK-01A	68 years, female, asian, stage:ii, alive, 1049 days	7.4
TCGA-CC-A8HV-01A	51 years, female, asian, stage:ii, dead, 279 days	7.2
TCGA-FV-A3I0-01A	76 years, female, white, stage:ii, alive, 848 days	7.2
TCGA-ED-A5KG-01A	60 years, female, asian, stage:ii, alive, 854 days	6.7
TCGA-BC-4072-01B	74 years, female, white, stage:iiia, dead, 1490 days	6.5
TCGA-FV-A23B-01A	70 years, female, white, stage:ii, dead, 1852 days	6.4
TCGA-ED-A66Y-01A	51 years, female, asian, stage:iiia, dead, 296 days	6.4
TCGA-CC-A7IJ-01A	56 years, male, asian, stage:ii, alive, 382 days	6.2
TCGA-ED-A66X-01A	35 years, male, asian, stage:iiia, alive, 406 days	6.2
TCGA-DD-A4NJ-01A	54 years, female, white, stage:ii, alive, 928 days	5.8
TCGA-ED-A97K-01A	54 years, male, asian, stage:iiia, alive, 6 days	5.6
TCGA-DD-A1EJ-01A	71 years, female, white, stage:iiic, dead, 1005 days	5.1
TCGA-ZS-A9CF-01A	64 years, male, white, stage:ii, alive, 2412 days	4.9
TCGA-ED-A8O5-01A	59 years, female, asian, stage:iiia, alive, 406 days	4.7
TCGA-FV-A4ZP-01A	78 years, male, white, stage:iiia, dead, 2486 days	4.6
TCGA-DD-A4NE-01A	75 years, female, white, stage:iiia, dead, 660 days	4.2
TCGA-DD-A114-01A	42 years, male, black or african american, stage:ii, dead, 1149 days	4.2
TCGA-RC-A7SB-01A	53 years, male, asian, stage:ii, alive, 588 days	4.1
TCGA-DD-A1EK-01A	64 years, female, white, stage:ivb, dead, 558 days	4.0
TCGA-UB-A7MA-01A	62 years, female, white, stage:ii, alive, 848 days	3.8
TCGA-DD-A3A9-01A	64 years, female, white, stage:ivb, dead, 931 days	3.5
TCGA-ED-A459-01A	47 years, male, asian, stage:ii, alive, 910 days	3.4
TCGA-CC-A3M9-01A	45 years, male, asian, stage:iiia, dead, 300 days	3.3
TCGA-G3-A7M9-01A	70 years, male, white, stage:iiib, dead, 56 days	3.3
TCGA-ED-A7PY-01A	20 years, female, asian, stage:ii, alive, 390 days	3.3
TCGA-K7-AAU7-01A	61 years, male, white, stage:ii, alive, 359 days	3.3
TCGA-CC-A7II-01A	55 years, male, asian, stage:iiia, alive, 399 days	3.2
TCGA-DD-A113-01A	55 years, female, white, stage:ii, alive, 2425 days	3.0
TCGA-DD-AADM-01A	58 years, male, asian, stage:ii, dead, 12 days	2.8
TCGA-BD-A3ER-01A	62 years, male, white, stage:ii, alive, 1115 days	2.8
TCGA-DD-AAVU-01A	46 years, male, asian, stage:ii, alive, 2202 days	2.7
TCGA-G3-A5SM-01A	58 years, male, white, stage:ii, alive, 520 days	2.5
TCGA-5R-AAAM-01A	65 years, female, white, stage:ii, dead, 46 days	2.4
TCGA-ZS-A9CD-01A	73 years, male, white, stage:ii, dead, 1386 days	2.4
TCGA-CC-A8HU-01A	39 years, female, asian, stage:iiia, dead, 344 days	2.3
TCGA-DD-A39Z-01A	43 years, female, stage:ii, dead, 601 days	2.3
TCGA-G3-A3CJ-01A	52 years, male, american indian or alaska native, stage:ii, alive, 594 days	2.3
TCGA-BC-A10X-01A	52 years, female, white, stage:iiia, dead, 770 days	2.3
TCGA-DD-A4NV-01A	61 years, male, white, stage:iiia, alive, 2398 days	2.1
TCGA-ED-A4XI-01A	58 years, male, asian, stage:ii, alive, 819 days	2.1
TCGA-CC-A5UC-01A	63 years, male, asian, stage:iiia, dead, 347 days	2.0
TCGA-DD-A116-01A	68 years, male, asian, stage:iiia, dead, 1622 days	2.0

TCGA-FV-A495-01A	51 years, female, white, stage:ii, alive, 1 days	2.0
TCGA-UB-AA0U-01A	60 years, male, white, stage:ii, alive, 327 days	1.9
TCGA-UB-A7MF-01A	57 years, male, white, stage:iiia, dead, 214 days	1.8
TCGA-CC-A7IE-01A	57 years, male, asian, stage:iiia, dead, 217 days	1.8
TCGA-MI-A75E-01A	61 years, male, white, stage:iiic, alive, 507 days	1.7
TCGA-CC-A5UD-01A	45 years, male, asian, stage:iiia, dead, 304 days	1.6
TCGA-2Y-A9H3-01A	45 years, male, white, stage:ii, alive, 1516 days	1.6
TCGA-G3-A3CH-01A	53 years, male, asian, stage:iiia, alive, 780 days	1.5
TCGA-CC-5260-01A	61 years, female, asian, stage:iiic, dead, 87 days	1.4
TCGA-ED-A7XO-01A	29 years, male, asian, stage:iiia, alive, 427 days	1.4
TCGA-G3-AAV6-01A	53 years, female, white, stage:iiia, dead, 65 days	1.3
TCGA-DD-A4NK-01A	80 years, female, white, stage:iiia, dead, 1210 days	1.3
TCGA-FV-A3I1-01A	female, white, stage:ii, dead, 247 days	1.3
TCGA-2Y-A9GY-01A	64 years, female, white, stage:ii, dead, 757 days	1.3
TCGA-YA-A8S7-01A	69 years, male, white, stage:iiia, dead, 412 days	1.3
TCGA-WX-AA46-01A	62 years, male, white, stage:ii, alive, 756 days	1.3
TCGA-CC-A7IF-01A	59 years, male, asian, stage:iiia, dead, 649 days	1.2
TCGA-CC-A1HT-01A	50 years, male, asian, stage:iiia, dead, 101 days	1.2
TCGA-DD-A119-01A	40 years, male, asian, stage:iv, dead, 223 days	1.1
TCGA-CC-A3MB-01A	36 years, male, asian, stage:iiia, dead, 315 days	1.1
TCGA-ED-A8O6-01A	50 years, female, asian, stage:iiia, dead, 56 days	1.0
TCGA-G3-A25X-01A	73 years, male, asian, stage:ii, alive, 1779 days	1.0
TCGA-CC-5261-01A	44 years, male, asian, stage:ii, dead, 97 days	1.0
TCGA-DD-A3A1-01A	65 years, male, stage:iiia, dead, 233 days	1.0
TCGA-5C-A9VG-01A	58 years, male, white, stage:ii, alive, 328 days	0.9
TCGA-CC-A3MC-01A	54 years, male, asian, stage:iiia, alive, 363 days	0.9
TCGA-CC-A123-01A	24 years, female, asian, stage:iiia, alive, 219 days	0.9
TCGA-GJ-A6C0-01A	75 years, female, white, stage:ii, dead, 31 days	0.9
TCGA-XR-A8TD-01A	49 years, female, white, stage:iiib, alive, 1030 days	0.8
TCGA-CC-A5UE-01A	48 years, male, asian, stage:iiib, dead, 272 days	0.8
TCGA-CC-A9FS-01A	55 years, male, asian, stage:ii, alive, 211 days	0.8
TCGA-LG-A9QD-01A	68 years, male, white, stage:iiia, alive, 366 days	0.8
TCGA-DD-AACG-01A	52 years, male, asian, stage:ii, dead, 469 days	0.8
TCGA-EP-A3RK-01A	73 years, male, white, stage:iiia, alive, 363 days	0.8
TCGA-CC-5258-01A	48 years, male, asian, stage:ii, dead, 129 days	0.7
TCGA-G3-A25T-01A	45 years, female, white, stage:iiia, alive, 1553 days	0.7
TCGA-CC-A8HT-01A	74 years, male, asian, stage:iiia, dead, 140 days	0.7
TCGA-5R-AA1D-01A	17 years, female, white, stage:iiia, alive, 449 days	0.6
TCGA-LG-A6GG-01A	79 years, female, white, stage:ii, alive, 387 days	0.6
TCGA-DD-AACH-01A	69 years, male, asian, stage:ii, dead, 195 days	0.6
TCGA-BC-4073-01B	73 years, male, white, stage:iiia, alive, 849 days	0.6
TCGA-CC-5259-01A	60 years, female, asian, stage:iiic, alive, 250 days	0.6
TCGA-CC-5263-01A	35 years, male, asian, stage:iiia, dead, 129 days	0.6

TCGA-DD-A4NA-01A	67 years, female, white, stage:iiic, alive, 1008 days	0.6
TCGA-DD-A4NG-01A	77 years, male, white, stage:iiia, dead, 802 days	0.5
TCGA-ED-A7PZ-01A	61 years, male, asian, stage:ii, alive, 6 days	0.5
TCGA-DD-AAVV-01A	56 years, male, asian, stage:ii, alive, 2455 days	0.5
TCGA-DD-A118-01A	77 years, female, white, stage:ii, alive, 3437 days	0.5
TCGA-CC-5262-01A	67 years, male, asian, stage:iiic, dead, 103 days	0.5
TCGA-RC-A6M5-01A	20 years, female, white, stage:iva, alive, 15 days	0.5
TCGA-DD-AAEK-01A	51 years, male, asian, stage:ii, alive, 1067 days	0.5
TCGA-DD-AADQ-01A	59 years, male, asian, stage:ii, alive, 436 days	0.5
TCGA-BC-A8YO-01A	66 years, female, white, stage:iiic, alive, 562 days	0.5
TCGA-G3-AAV7-01A	38 years, male, asian, stage:ii, alive, 361 days	0.5
TCGA-DD-A3A8-01A	75 years, male, white, stage:ii, dead, 11 days	0.4
TCGA-G3-A5SI-01A	44 years, male, asian, stage:ii, dead, 768 days	0.4
TCGA-DD-AAVX-01A	38 years, male, asian, stage:ii, alive, 1570 days	0.4
TCGA-DD-A4NI-01A	67 years, male, white, stage:ii, alive, 816 days	0.4
TCGA-DD-A39V-01A	77 years, male, white, stage:ii, dead, 643 days	0.4
TCGA-4R-AA8I-01A	66 years, male, white, stage:ii, dead, 262 days	0.4
TCGA-DD-A1EA-01A	68 years, male, asian, stage:ii, alive, 2415 days	0.4
TCGA-BC-A69H-01A	64 years, male, white, stage:ii, alive, 444 days	0.4
TCGA-DD-A1EE-01A	73 years, male, white, stage:iiia, dead, 349 days	0.3
TCGA-PD-A5DF-01A	58 years, female, white, stage:iiib, dead, 639 days	0.3
TCGA-RC-A6M4-01A	74 years, female, white, stage:iiia, alive, 22 days	0.3
TCGA-DD-A3A6-01A	72 years, female, white, stage:ii, dead, 3258 days	0.3
TCGA-DD-A4NQ-01A	60 years, male, white, stage:ii, dead, 373 days	0.3
TCGA-DD-A115-01A	53 years, male, white, stage:iiia, dead, 2542 days	0.3
TCGA-G3-AAV3-01A	58 years, female, white, stage:ii, alive, 412 days	0.3
TCGA-DD-AACX-01A	66 years, male, asian, stage:ii, alive, 170 days	0.3
TCGA-DD-AACI-01A	69 years, male, asian, stage:ii, alive, 1618 days	0.3
TCGA-BC-A217-01A	75 years, female, white, stage:ii, dead, 1397 days	0.3
TCGA-KR-A7K7-01A	61 years, female, white, stage:ii, alive, 951 days	0.2
TCGA-2Y-A9HA-01A	70 years, male, white, stage:ii, dead, 36 days	0.2
TCGA-DD-AAD6-01A	66 years, male, asian, stage:iiia, alive, 672 days	0.2
TCGA-RC-A6M6-01A	75 years, male, white, stage:ii, alive, 9 days	0.2
TCGA-G3-AAV1-01A	51 years, male, asian, stage:iiic, dead, 359 days	0.2
TCGA-WQ-AB4B-01A	62 years, male, white, stage:ii, alive, 395 days	0.2
TCGA-DD-A1EL-01A	23 years, male, black or african american, stage:ii, dead, 415 days	0.2
TCGA-DD-AACQ-01A	50 years, male, asian, stage:ii, dead, 432 days	0.2
TCGA-ED-A7XP-01A	53 years, female, asian, stage:ii, alive, 400 days	0.2
TCGA-RG-A7D4-01A	69 years, male, black or african american, stage:ii, alive, 1098 days	0.2
TCGA-2Y-A9H0-01A	49 years, male, white, stage:iiia, alive, 3675 days	0.2
TCGA-CC-A7IG-01A	47 years, male, asian, stage:ii, dead, 299 days	0.2
TCGA-DD-AAW1-01A	55 years, male, asian, stage:iiia, alive, 1989 days	0.2
TCGA-G3-AAV5-01A	67 years, male, white, stage:ii, alive, 354 days	0.2

TCGA-CC-A9FW-01A	68 years, male, asian, stage:iiia, alive, 248 days	0.2
TCGA-EP-A2KA-01A	52 years, female, white, stage:iiia, dead, 627 days	0.2
TCGA-BW-A5NO-01A	50 years, male, black or african american, stage:iiia, alive, 20 days	0.1
TCGA-DD-A73C-01A	65 years, female, white, stage:iiia, alive, 701 days	0.1
TCGA-BC-A216-01A	62 years, female, white, stage:iiia, alive, 1351 days	0.1
TCGA-5R-AA1C-01A	57 years, male, white, stage:ii, alive, 520 days	0.1
TCGA-G3-A6UC-01A	65 years, male, white, stage:iiib, alive, 671 days	0.1
TCGA-ZS-A9CE-01A	79 years, female, white, stage:ii, alive, 1241 days	0.1
TCGA-DD-AACJ-01A	75 years, male, asian, stage:ii, alive, 2102 days	0.1
TCGA-2Y-A9GZ-01A	82 years, female, white, stage:ii, dead, 848 days	0.1
TCGA-MI-A75G-01A	63 years, male, white, stage:ii, alive, 698 days	0.1
TCGA-NI-A4U2-01A	71 years, male, white, stage:iiia, dead, 1791 days	0.1
TCGA-CC-A7IH-01A	58 years, male, asian, stage:iiia, alive, 365 days	0.1
TCGA-3K-AAZ8-01A	65 years, male, black or african american, stage:iiib, alive, 396 days	0.1
TCGA-BC-A3KG-01A	68 years, female, white, stage:ii, alive, 680 days	0.1
TCGA-UB-A7MC-01A	59 years, male, white, stage:iiia, alive, 500 days	0.1
TCGA-QA-A7B7-01A	48 years, male, black or african american, stage:ii, alive, 94 days	0.1
TCGA-DD-AAVY-01A	56 years, male, asian, stage:iiia, alive, 1970 days	0.1
TCGA-G3-A5SL-01A	70 years, male, white, stage:ii, alive, 621 days	0.1
TCGA-CC-5264-01A	71 years, male, asian, stage:iiia, dead, 102 days	0.1
TCGA-XR-A8TE-01A	16 years, male, white, stage:iiia, alive, 925 days	0.1
TCGA-DD-A39W-01A	29 years, female, white, stage:iii, dead, 827 days	0.1
TCGA-RC-A7SH-01A	42 years, male, asian, stage:ii, alive, 468 days	0.1
TCGA-UB-A7MB-01A	24 years, male, white, stage:ii, alive, 601 days	0.1
TCGA-ZS-A9CG-01A	55 years, male, white, stage:ii, alive, 341 days	0.1
TCGA-CC-A7IK-01A	59 years, male, asian, stage:iiia, dead, 262 days	0.1
TCGA-DD-A3A7-01A	67 years, male, stage:iiib, dead, 419 days	0.1
TCGA-DD-A3A5-01A	66 years, female, white, stage:iii, dead, 3125 days	0.0
TCGA-DD-AADG-01A	70 years, male, asian, stage:iiia, alive, 1145 days	0.0
TCGA-WX-AA47-01A	33 years, female, white, stage:iiia, dead, 556 days	0.0
TCGA-DD-AADU-01A	60 years, male, asian, stage:ii, alive, 554 days	0.0
TCGA-DD-A3A4-01A	37 years, male, white, stage:iiia, dead, 612 days	0.0
TCGA-DD-A73D-01A	68 years, female, white, stage:ii, alive, 693 days	0.0
TCGA-CC-A7IL-01A	61 years, male, asian, stage:iiia, dead, 278 days	0.0
