# Supplementary material for: Inhibition of TRPV4 remodels single cell polarity and suppresses the metastasis of hepatocellular carcinoma
Source: Cell Death Dis. 2023 Jun 28;14(6):379. doi: 10.1038/s41419-023-05903-z (PMC10300155; doi:10.1038/s41419-023-05903-z)
Supplement: Supplementary file 3 — Supplementary Materials and Methods [file 41419_2023_5903_MOESM3_ESM.pdf]

Supplementary Materials and Methods

1. Wound healing assay

The digested cells were cultured in six-well plate. When the confluence reached more than 90%, drew a vertical trace with a 100 μL sterile pipette tip. Then we used PBS to wash floating cells three times and treated cells with compounds. After treatment, the inverted microscope photographed the same position. The pictures were quantitatively processed with Imagine J. Three different fields of view were selected in each group, and the area of the wound scratch was measured by ImageJ. Scratch healing rate is calculated as below:

Scratch healing rate (%) = (Scratch area $_{0\ h}$ – Scratch area $_{48\ h}$) / Scratch area $_{0\ h}$ ×100%.

2. Transwell migration and transwell invasion assay

For transwell migration assay, we resuspend the cells in serum-free medium and seed them in the upper chamber (Corning Costar, Cambridge, MA, USA) at a density of 30,000 cells per well (100 uL). The upper compartment was then filled with the same volume of serum-free medium with or without compound. Then 15% (MHCC-97H) or 20% (HCC-LM3) serum concentration medium was added to the lower chamber, and the upper and lower chambers were incubated together for 24 h. The obtained transwell chamber was first gently wiped off the remaining cells in the upper chamber, then fixed with 4% paraformaldehyde, stained with 0.1% crystal violet, and photographed with an inverted microscope.

For transwell invasion assay, 6 mg/mL Matrigel (80 uL) was coated in the transwell chamber in advance, and place it at 37°C to solidify for 1 h. The subsequent operation was the same as the transwell migration experiment. Three pictures were taken in each chamber and the experiment was repeated three times. Finally, Imajine J was used to quantify the acquired data.

3. Cell proliferation assay

The viability of the adherent cells was measured using 3-[4, 5-di-methylthiazol-2-yl]−2, 5-diphenyltetrazolium bromide (MTT) assay. Cells were cultured in 96-well plate and treated with different concentrations of GL-V9 (0-128 μM). After treatment for 24 or 48 h, each well added 20 μLof MTT solution (5 mg/mL). The plate was incubated at 37 °C for 4 h in a humidified atmosphere with 5% CO2. The supernatants were removed carefully and the formazan was dissolved in 100 μL DMSO. The absorbance was measured spectrophotometrically at 570 nm by the Universal Microplate Reader ELx800 (BIO-TEK instruments, Inc., Vermont, MA). The inhibition ratio was calculated as the

following formula: $(A_{control} - A_{treated}) / A_{control} \times 100\%$. The IC_{50} was taken as the concentration that caused 50% inhibition of cell proliferation. We fit the data using an equation for a sigmoidal dose-response provided by GraphPad™ Prism® 4.0 to determine the IC_{50} value.

The suspended MHCC-97H cell viability in poly-HEMA coated 96-well plate was measured by CCK-8 Cell Counting Kit (Vazyme, Nanjing, China). The absorbance value of 96-well plate was measured at 450 nm wavelength. The cell inhibition rate and IC_{50} value acquisition were the same as MTT experiment.

4. Annexin V/PI staining assay

Cells were treated and harvested. Apoptotic cells were identified by Annexin V/PI Cell Apoptosis Detection Kit (Vazyme Biotec, Nanjing, China), according to the protocols. Flow cytometric analysis was used immediately to detect the apoptosis rate. Data acquisition and analysis were performed in a Becton–Dickinson FACSCalibur flow cytometer.

5. Quantitative real-time-polymerase chain reaction (RT-qPCR) assay

Total RNA was extracted from the cells using an RNA isolator (Vazyme, Nanjing, China) according to the manufacturer's instructions. One microgram of total RNA was used to synthesize first-strand cDNA using HiScript II First-strand cDNA Synthesis Kit (Vazyme, Nanjing, China). Real-time PCR was performed using SYBR Green PCR Master Mix (Vazyme, Nanjing, China) in ABI PRISM Sequence Detector 7500 (PerkinElmer, Branchburg, NJ).

The RT-qPCR primer sequence was as follows:

TRPV4 forward, 5’-GACGGGGACCTATAGCATCA-3’;

TRPV4 reverse, 5’-AACAGGTCCAGGAGGAAGGT-3’;

GAPDH forward, 5’- AACAGCGACACCCACTCCTC-3’;

GAPDH reverse,5’- GGAGGGGAGATTCAGTGTGGT-3’;

The relative quantification method($2^{-\triangle\triangle Ct}$)was used to calculate the fold change of mRNA.

6. Immunofluorescence

The suspension-grown cells were collected and smeared on glass coverslips. Then the coverslips were fixed with 4% paraformaldehyde for 15 min and permeabilized with 0.5% Triton X-100 on ice for 30 minutes. After permeabilization, the cells were blocked with PBS containing 3% BSA for 1 h, and incubated with ERM (1:200, Abbkine) and p-ERM (1:100, abcam) antibodies overnight. After washing

twice with cold PBS, the cells were stained with FITC-conjugated goat anti-rabbit IgG secondary antibody (1:200, abcam) for 1 h. Then the coverslips were washed for two times with cold PBS and incubated with rhodamine-labeled phalloidin (1:200, YEASEN, Shanghai, China) for 30 min. Ezrin (1:100, abcam) and F-actin (1:100, abcam) antibodies were used following the same method as p-ERM. Images were captured using a confocal microscope (Olympus FV1000, Olympus Corp., Tokyo, Japan).

7. Immunohistochemistry

The liver and lung were fixed in 4% paraformaldehyde, then sectioned with paraffin. The tissue sections were heated, fixed, deparaffinized and rehydrated in a gradient of decreasing amounts of alcohol and, finally, distilled water. The slides were heated in citric acid buffer for antigen repair. Then, tissue section permeabilization was performed with 0.5% Triton-X/PBS. The slides were treated with 5% goat serum and 0.5% BSA at room temperature for 1 h and incubated overnight with primary antibodies at 4 °C. The primary antibodies included antibodies against Ki67 (Proteintech, 27309-1-AP; 1:2000), P-ERM (abcam, ab76247; 1:200), P-MLC (Cell Signaling Technology, 3671T; 1:400), and P-AMPK (ABclonal, AP1002; 1:200). Finally, the sections were stained with DAB dye, sealed with neutral resin, observed under a microscope and imaged.

8. Cellular Thermal Shift Assay (CETSA)

MHCC-97H cells were treated with 70 μM GL-V9 for 1 h, then harvested and resuspended in PBS containing protease inhibitors, and aliquoted into 200 μL tubes. The cells were heat shocked in a T960 thermal cycler (Heal Force, Hangzhou, China) at 37.0 to 64.0 °C for 3 min to denature the proteins, and then, all the samples were subjected to three freeze-thaw cycles with liquid nitrogen to lyse cells. The samples were centrifuged at 14,000 rpm for 20 min at 4 °C. Loading buffer was added, and the proteins were analyzed by Western blotting assay.

9. Cell motility measurement through live cell workstation

After 24 h of treatment with or without GL-V9, the suspended MHCC-97H cells were seeded in an anti-adherent culture dish at a cell density of 12,500/mL, and the administration of GL-V9 group continued. The petri dish was placed in a small incubator at 37°C and 5% CO_2 conditions and a live cell workstation (Nikon-Eclipse-Ti, Japan) was used to take a picture every 5 minutes with the shooting time 24 h. The obtained data was processed with Imagine J to obtain the cell movement distance and movement speed parameters. The experiment was repeated three times.
